# Evaluating the Significance of Paleophylogeographic Species Distribution Models in Reconstructing Quaternary Range-Shifts of Nearctic Chelonians

**DOI:** 10.1371/journal.pone.0072855

**Published:** 2013-10-09

**Authors:** Dennis Rödder, A. Michelle Lawing, Morris Flecks, Faraham Ahmadzadeh, Johannes Dambach, Jan O. Engler, Jan Christian Habel, Timo Hartmann, David Hörnes, Flora Ihlow, Kathrin Schidelko, Darius Stiels, P. David Polly

**Affiliations:** 1 Zoologisches Forschungsmuseum Alexander Koenig, Bonn, Germany; 2 National Institute for Mathematical and Biological Synthesis (NIMBioS), University of Tennessee, Knoxville, Tennessee, United States of America; 3 Department of Biogeography, Trier University, Trier, Germany; 4 Department of Geological Sciences, Indiana University, Bloomington, Indiana, United States of America; University of York, United Kingdom

## Abstract

The climatic cycles of the Quaternary, during which global mean annual temperatures have regularly changed by 5–10°C, provide a special opportunity for studying the rate, magnitude, and effects of geographic responses to changing climates. During the Quaternary, high- and mid-latitude species were extirpated from regions that were covered by ice or otherwise became unsuitable, persisting in refugial retreats where the environment was compatible with their tolerances. In this study we combine modern geographic range data, phylogeny, Pleistocene paleoclimatic models, and isotopic records of changes in global mean annual temperature, to produce a temporally continuous model of geographic changes in potential habitat for 59 species of North American turtles over the past 320 Ka (three full glacial-interglacial cycles). These paleophylogeographic models indicate the areas where past climates were compatible with the modern ranges of the species and serve as hypotheses for how their geographic ranges would have changed in response to Quaternary climate cycles. We test these hypotheses against physiological, genetic, taxonomic and fossil evidence, and we then use them to measure the effects of Quaternary climate cycles on species distributions. Patterns of range expansion, contraction, and fragmentation in the models are strongly congruent with (i) phylogeographic differentiation; (ii) morphological variation; (iii) physiological tolerances; and (iv) intraspecific genetic variability. Modern species with significant interspecific differentiation have geographic ranges that strongly fluctuated and repeatedly fragmented throughout the Quaternary. Modern species with low genetic diversity have geographic distributions that were highly variable and at times exceedingly small in the past. Our results reveal the potential for paleophylogeographic models to (i) reconstruct past geographic range modifications, (ii) identify geographic processes that result in genetic bottlenecks; and (iii) predict threats due to anthropogenic climate change in the future.

## Introduction

Quaternary glacial-interglacial cycles have caused dramatic range shifts of species' distributions [1]–[5]. Many species in high- and mid-latitudes have been extirpated from northern and high altitude areas, often accompanied by range fragmentation and persistence in so-called glacial refugia [6]–[9]. These geographic processes have led to phylogeographic differentiation at the population, subspecies and species levels in both plants and animals [10]–[11]. Range fragmentation has led to allopatric genetic differentiation, and range expansions have led to the formation of tension hybrid zones, processes that lead to genetic diversity [8], [12]–[15]. Large-scale geographic range contractions have also resulted in the loss of genetic diversity through repeated founder effects and population bottlenecks [16]. Species can respond to changing climate in two non-exclusive ways: (i) adapting its climatic niche to fit the new conditions or (ii) changing its geographic range to track the conditions that are compatible with its climatic niche [17]–[19]. One of the biggest gaps in our understanding of biotic responses to climate change is the relative balance between the two. Many studies have addressed geographic range tracking in relation to past and future climates [20]–[25], but only a few have attempted to evaluate the rates and magnitudes by which species respond by adapting to climate change (e.g. [26]–[29]). This area is in urgent need of investigation because it is highly relevant to assessing the impact of rapidly changing climate.

This study integrates the Quaternary fossil record, the oxygen isotopic record of global temperature history, paleoclimatic models of the geographic distribution of past climates, phylogenetic comparative analysis, and species distribution modeling to better understand the tempo and mode of how turtle species have responded to past climate changes. These integrated data are used to produce testable, geographically explicit, temporally continuous models of species ranges through the full range of climate variability of the last three Quaternary climate cycles. We test our models against fossil occurrence data, which are the only positive source of information about where species occurred in the past. The fossil occurrences test whether the past geographic ranges of these turtle species can accurately be reconstructed from paleoclimate, which is one of the fundamental assumptions of our paleophylogeographic modeling approach, indeed to all ecological niche modeling. The models are then used to evaluate the roles that past changes in climate and geography have played in determining physiological, genetic, and phylogeographic patterns. Finally, our results are used to partition the responses of turtles to climate change into phylogenetic (adaptive) and purely geographic components to assess the relative contribution of each mode.

We call our approach paleophylogeographic modeling. It is derived from species distribution modeling (SDM), uses georeferenced occurrences to characterize the range of climate that a species actually inhabits (the realized climate niche) and which has become the method of choice for projecting modern climatic niches onto paleoclimate reconstructions [30]–[32]. By assuming that the realized niche actually represents its true climatic niche (the fundamental climate niche) [24], the expected geographic range of a species can be determined for any future and past climate model. Our paleophylogeographic approach expands on the SDM approach in several important ways: (i) it uses a temporally continuous model of changing paleoclimate with which to model changes in turtle ranges through the past 350 Ka; (ii) it uses phylogenetic comparative methods to account for evolutionary changes that happened in the climate niche that occurred over the period being modeled; (iii) it uses fossil occurrences to test whether the reconstructed ranges are accurate; (iv) it also uses the fossil occurrences to test the core assumption that the modern realized niche is a good proxy for the fundamental climate niche; and (v) it partitions past responses to climate change into adaptive and purely geographic components so that the rates of climatic adaptation and geographic range tracking can be compared.

**Figure 1:**
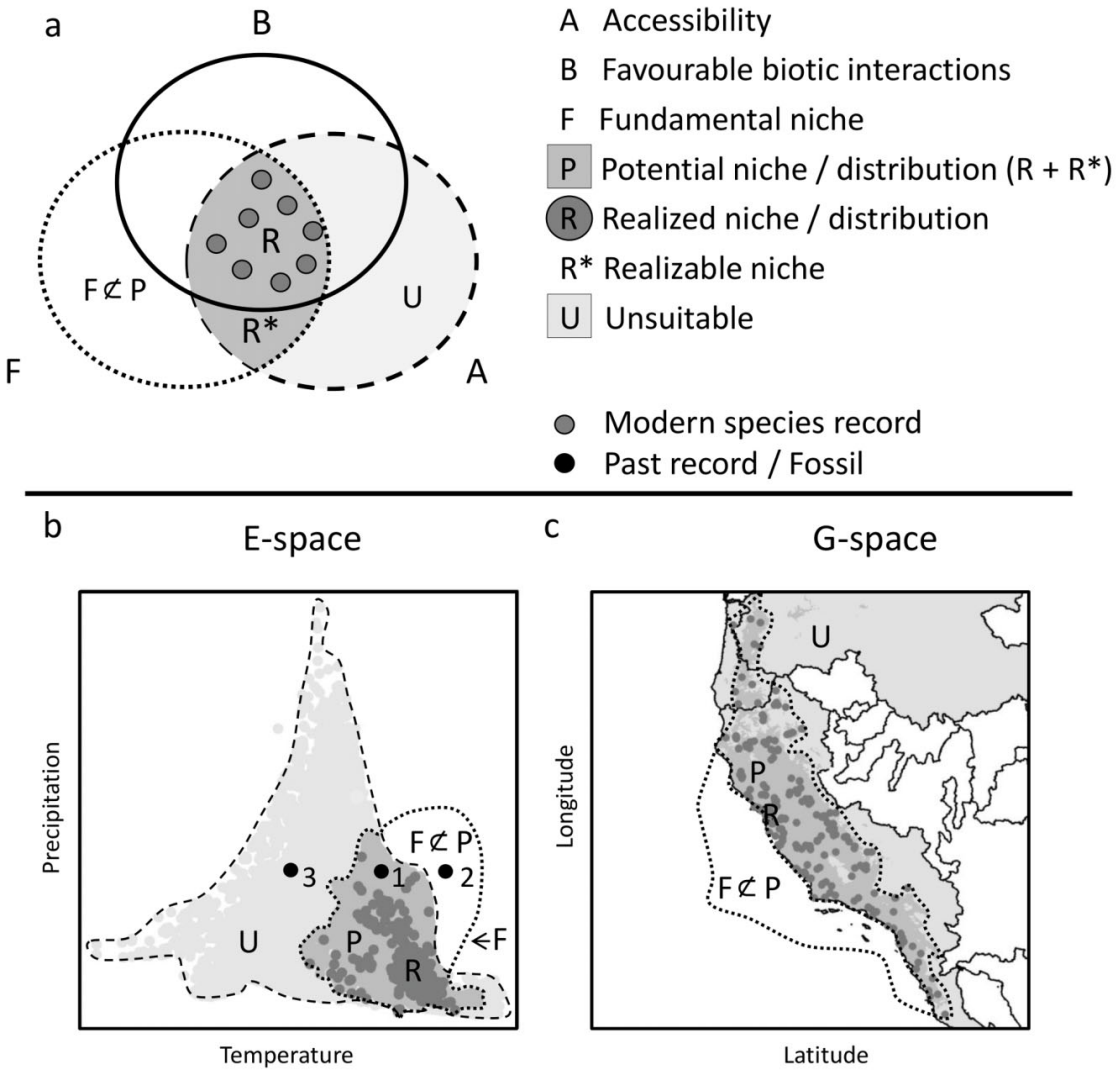
Relationships between fundamental niches, realized niches, potential niches and geographic distributions as (a) a Venn diagram (after [36], [157]); (b) in climatic niche space (E-space); and (c) in geographic space (G-space). A species' realized niche (R) is the intersection of its fundamental niche (F), its accessible climate or territory (A), and the climate and territory not barred by biotic interactions (B). Its potential niche (P) is the subset of the fundamental niche for which there is available climate (A), and its potential distribution is the territory with climate tolerable to the species. (F ∩ P) is the range of climate or geography that is climatically tolerable to a species but which is not accessible because of the lack of climate availability (E-space) or geographic barriers (G-space). A fossil may occur in E-space within the species' potential distribution (1), outside the range of available paleoclimate (2), or in paleoclimates which are available but actually not suitable (3). See text for details.

The conceptual framework of our paleophylogeographic modeling, like species distribution modeling, is based on the concepts of niches in climate space and their relationship to distributions in geographic space [33]–[35] (Figure 1). Hutchinson conceptualized niches as volumes within a multidimensional space whose axes are variables important to the life of the species [35]. Grinnell, who first introduced the niche concept, limited the concept to abiotic environmental conditions, especially climate [34]. We adopt Grinnell's strictly climatic view of the niche because we are focusing on the effects of Quaternary climatic changes on species ranges. Thus, in our study, the multidimensional niche space is defined by climatic variables (E-space, where “E” stands for environmental). The distribution of a species in this niche space is closely related to its distribution in geographic space (G-space), and vice versa. The niche concept can be logically subdivided into fundamental and realized niches [36]–[39]. A fundamental niche is the full range of E-space in which a species can flourish – its range of climatic tolerance. The realized niche is the range of E-space that the species actually occupies, which may be smaller. Similarly, a realized geographic distribution is the range of territory in G-space that it actually occupies. Both biotic and accessibility factors can prevent a species from realizing the full niche space or territory where it could otherwise flourish based on its climate tolerances. In E-space, biotic exclusion occurs when one species excludes another from part of the climate it can tolerate, in G-space when one species excludes another from a geographic area. Accessibility in E-space occurs where climate is actually available for the species, whereas in G-space species accessibility occurs when there are no geographic barriers, such as oceans, mountains, or rivers, to prevent a species from occupying a territory that it finds climatically tolerable.

Accessibility in E-space is an important concept in our study, and it relates to what we call analogue and non-analogue climates [40]–[41]. Some areas of E-space represent climate that does not actually occur in G-space, which means that there is no “accessible climate” for the species in E-space. For example, consider a mean annual temperature of 50°C, which is far greater than any mean annual temperature found on Earth today and would not have a corresponding point in G-space. Points such as this in E-space are called non-analogue climate points. Some non-analogue climates actually existed in the geological past [40]–[41]. For example, in the early Eocene mean annual temperature was more than 12°C higher than it is today and there were no areas of the globe where low temperatures dropped below the freezing point. The maximum mean annual temperature of the early Eocene world fell well above the maximum boundary in E-space defined by modern climate and was thus a non-analogue with respect to modern climate. Conversely, today's minimum mean annual temperature falls well below the minimum boundary in E-space defined by early Eocene climate and could thus be described as non-analogue with respect to Eocene climate. Accessibility in E-space is thus something that changes as climate changes. In our study, changes in climate accessibility provide important clues to how closely the realized niche approximates the fundamental niche.

In this paper we make another important distinction about *potential* niches and geographic range [42]. A *potential range* is the territory where a species is capable of flourishing and which is accessible to it (Figure 1A,C). In other words, it is the territory where we expect a species to live based on its fundamental niche (climate tolerances) in the absence of biotic barriers but in the presence of geographic barriers. A *potential niche* is the climatic space that falls within the fundamental niche and which actually exists at the time a species lives (available climate). The potential range of a species changes as climate changes, as can its potential niche change as the range of available climate changes. We make use of the changes in potential niches through the last 320 Ka of Quaternary climate change to assess the extent to which turtle species could live in climates that do not exist in the present day.

We make use of fossils in two ways, first to test whether modern niches are congruent with niches in the past by testing the power of modern realized niches to predict past geographic ranges that are compatible with fossil occurrences once phylogenetic changes and changes in available climate have been taken into account. Previous studies that have used fossils as tests have found that SDMs are usually successful at regional and continental scales in estimating the past ranges of species, the location of glacial refugia, and the extent of postglacial range expansions (e.g. [18], [23], [26]–[27], [43]–[45]. Furthermore, SDMs constructed from fossil occurrences and paleoenvironmental data have often successfully predicted modern geographic distributions [46]–[47]. However, there are notable exceptions in which SDMs grossly failed to agree with fossil data or molecular phylogeographic data [27], [30], [43], [48]–[49]. Thus, we recommend that paleogeographic range reconstructions always be tested against fossil data if at all possible. Independent lines of evidence such as physiological data, the fossil record and genetic signatures offer important insights into niche dynamics [18], [49]–[50]. We make full use of these tests in this study.

We also use fossils to study changes in the potential niche by determining whether species occupied non-analogous climate conditions in the past, which in turn helps better understand whether changes in the niche over time are due to changes in biotic factors, like interspecies competition, adaptive changes in the niche, or changes in the climate system itself. Non-analogous climates can easily be identified by plotting modern and paleoclimate data in the same E-space: non-analogue climates are simply those paleoclimate data points that fall outside the boundaries of modern climate. A fossil can fall in one of three places in E-space: (1) inside the species potential niche, (2) outside the available paleoclimate, or (3) inside the available paleoclimate but outside the potential niche (Figure 1B). The first case indicates that the potential niche as estimated from the modern realized niche is compatible with the fossil evidence; the second indicates that either the fossil or the paleoclimate data are erroneous; the third indicates that the species occurred in an available climate outside the range predicted by its modern tolerances, which suggests that its niche has changed over time. These three outcomes help discriminate between changing climate accessibility and evolving fundamental niches.

We used multivariate environmental similarity surfaces (MESS) to measure the positions of fossil occurrences in E-space [51]–[52]. MESS is the Euclidean distance of any given point in E-space to boundaries defined by a set of reference points. The MESS score is positive when the point lies within the reference boundaries and negative when it lies outside them. Non-analogous climate points are thus those points with negative MESS scores that lie outside the boundaries of modern climate (for more details see below). We also used MESS scores to measure the position of fossil occurrences of turtle species, with respect to the boundaries of their realized and potential niches, to determine whether the niches have changed over the last 320 Ka. The MESS scores thus indicate whether a species has tolerated climatic conditions beyond what it experiences today, they help define the full potential niche of the species, and they help forecast the fate of the species over the next century.

An important feature of our paleophylogeographic approach is that we use phylogeny to estimate the evolutionary changes that have occurred in climate tolerances over a clades' history. Because of evolution, we cannot expect *a priori* that a species' niche in the past was the same as it is today [23], [30], [49], [53]. The logic of this approach is based on the observation that the climatic niches of modern members of a clade differ from one another, whereas the ancestor of the clade would have had a single climatic niche. Using phylogenetic comparative methods, ancestral niches can be estimated at each node and along each branch of the tree [45], [54]–[57]. We use the phylogenetic information to adjust each species niche in E-space for where it is along the phylogenetic branch leading back to its common ancestor with other species [23]. The phylogenetic changes can be partitioned from geographic tracking in our paleophylogeographic models [23] and thus help us address which mode of response has been more important in the clade's history.

Turtles and tortoises are a good group for studying the response of species to climate change because: (i) their current distributions are well documented [58]–[60]; (ii) they have a good Quaternary fossil record (e.g. [61] with references therein); (iii) their phylogenetic relationships are well resolved [62]; and (iv) phylogeographic data based on molecular markers are available for many species (Table 1). Even more importantly, turtles and tortoises are ectotherms that are more directly affected by ambient temperature than mammals and birds [55], [63] and thus are expected to have realized niches that are more directly controlled by climate. Indeed, incubation temperature determines sex in most species ([64] and references therein). Fifty nine of the 61 living North American turtles are included in this study.

**Table 1:** Comprehensive overview of phylogeographic and morphometric analyses of North American turtle species.

Species	Marker	Diversity/bottleneck	differentiation	Time	Reference
*Actinemys marmorata*	mt/nuclear DNA sequences	Low diversity caused by geographically restricted refugia (x)	Four well supported clades: large Northern clade composed of populations from Washington south to San Luis Obispo County, California, west of the Coast Ranges; a San Joaquin Valley clade from the southern Great Central Valley; a geographically restricted Santa Barbara clade from a limited region in Santa Barbara and Ventura counties; and a Southern clade that occurs south of the Tehachapi Mountains and west of the Transverse Range south to Baja California, Mexico. (1)	old	[103]
*Apalone ferox*	mtDNA	Genetic bottlenecks caused due to dispersal into postglacial and glacial habitats (1)	Historical vicariant processes during the Pliocene, genetic break between northern–western and south-eastern populations into seven lineages. (1)	old	[104]
*Apalone mutica*	mtDNA	NA	Strong intraspecific vicariance, split into five clades and two main groups west versus east; *A. mutica mutica*, *A. mutica calvata*. (1)	old	[104]
*Apalone spinifera*	mtDNA	NA	Strong intraspecific vicariance, split into seven clades and two main groups (west versus east). (1)	old	
	Mt/ncDNA: CytB, R35, RAG-1, Cmos	High genetic variability, no genetic bottlenecks (1)	Six lineages e.g. subspecies: south/eastern/northern: *A. spinifera aspera, A. spinifera hartwegi, A. spinifera spinifera*, western: *A. spinifera pallida, A. spinifera emoryi, A. spinifera guadalupensis.* (1)	old	[105]
*Chelydra serpentina*	mtDNA, allozymes	Low variation, even in allozymes (x)	mtDNA support the species-level distinctness of *C. serpentina rossignonii* and *C. serpentina acutirostris* from each other and from *C. serpentina serpentina* – *C. serpentina osceola* complex (today recognized as full species); allozyme variation too low for subspecies detection. (x)	old	[141]
	mtDNA: control region	Low mtDNA variation, unusual for a widely distributed species (x)	No genetic structure (x)	old	[106]
*Chrysemys dorsalis*	mtDNA control region	Low mtDNA variation (0.15–0.45%) (1)	Lowest mtDNA variation compared with the *C. picta* complex (see below) (1)	old	[107]
*Chrysemys picta*	mtDNA control region	mtDNA variation *C. p. belli*: 0.15–1.37%; *C. p. picta*: 0.15–1.21%; *C. p. marginata*:0.15–1.51%; (1)	Two distinct main groups (three lineages, *C. picta picta*, *C. picta belli*, *C. picta marginata*): Massive extinction/recolonization event across the Great Plains/Rocky Mountain region encompassing over half the continental United States. (1)	old	[107]
*Deirochelys reticularia*	Morphology		Three morphologically distinct subspecies, *D. reticularia reticularia* in south eastern US; *D. reticularia miaria* on the Florida panhandle and *D. reticularia chrysea* in the lower Mississippi basin to northern Texas. Split between eastern and western forms is well supported by our models. (1)		[142]
*Emydoidea blandingii*	5 μSats	Low diversity (1)	Three distinct groups: (pairwise FSTs = 0.042–0.124; p<0.05) (1)	old	[117]
	5 μSats	Low diversity (1)	Appalachian Mountains and the Hudson River appear to present major barriers to gene flow; Appalachian Mountains as well as the highly disjunct Nova Scotia populations of Blanding's turtle are recognized as evolutionarily significant units. (1)	old to recent	[143]
*Glyptemys insculpta*	mtDNA control region	21 haplotypes, low diversity, bottleneck during the Pleistocene (1)	Little genetic differentiation (highest pairwise difference was 2%), one main postglacial dispersal route was inferred along the east coast, with subsequent westwards dispersal and one common southern refugium. (1)	old	[108]
*Glyptemys muhlenbergii*	mtDNA; cytb, nd4, and d-loop	Bottleneck in refugia and subsequent rapid post-Pleistocene expansion into the north (1)	Low level of divergence. (1)	old	[109]
*Graptemys gibbonsi*	Morphometric, Nuclear and mtDNA (mtDNA, control region, ND4)	NA	Shallow genetic and morphologic differentiation between the Pearl and Pascagoula river samples of *G. gibbonsi* (p-distances: ND4 0.0/mtDNA control region 0.013) (1)	recent	[110]
*Graptemys nigrinoda*	Morphology	NA	Two subspecies, *G. nigrinoda nigrinoda* and *G. nigrinoda delticola* with an intermediate form occurring in large parts of the range. (x)	old	[144]
*Graptemys ouachitensis*	mtDNA	Low haplotype variation (1)	Two main clades dividing the species into two subspecies: *G. ouachitensis ouachitensis* in the north and *G. oachitensis sabinensis* in the south. (1)	old	[145]
*Graptemys pseudogeographica*	Morphology	NA	Two subspecies, *G. pseudogeographica pseudogeographica* in the northern and *G. pseudogeographica kohnii* in the southern Mississippi basin. (x)	old	[62]
*Kinosternon baurii*	mtDNA; control region	High haplotype diversity due to relatively stable refugium (1)	*K. baurii* forms one group at the Atlantic coastal states. (1)	old	[146]
*Kinosternon hirtipes*	Morphology	NA	Six subspecies, *H. hirtipes hirtipes, H. hirtipes chapalaense, H. hirtipes magdalense, H. hirtipes megacephalum* (†), *H. hirtipes murrayi, H. hirtipes tarascense*. (1)		[62]
*Kinosternon subrubrum*	mtDNA; control region	Very high genetic diversity (due to large and relatively stable refugia) (1)	Four major lineages with two main splits (west versus east): Western group (Missouri, Louisiana), Central Group (Gulf coastal states), eastern group (Atlantic coastal states) southern group (Florida); *K. baurii* belongs to eastern group. *K. baurii* and *K. subrubrum* in Florida are highly distinct, but exhibit minimal mtDNA divergence along the Atlantic coastal states. (1)	old	[146]
*Macrochelys temminckii*	mtDNA; μSats	Range-wide consistently low within-population mtDNA and microsatellite diversity (1)	Strong differentiation: Six evolutionarily significant units are recommended on the basis of reciprocal mtDNA monophyly and high levels of microsatellite DNA divergence, Suwannee River population might eventually be recognized as a distinct taxonomic unit. (1)	old	[126]
	mtDNA control region	Low intraspecific diversity, major proportion of genetic diversity is restricted to lineages (caused by distinct and small refugia) (1)	Strong differentiation into three groups (eastern, central and western portion of the species' range) coinciding with three recognized biogeographic provinces. (1)		[147]
*Malaclemys terrapin*	mtDNA; cytochrome *b* and control region	mtDNA genotypic diversity (G = 0.582) very low despite large and relatively stable refugia (NA due to anthropogenic translocation)	Exceptionally low genotypic diversity and divergence levels. (NA due to anthropogenic translocation)	old	[148]
	Mark-Release-Recapture, 6 μSats	NA	Low genetic differentiation, with isolation by distance, probably a pattern caused by translocations; (NA due to anthropogenic translocation)	recent	[125]
*Pseudemys cocinna*	Morphology	NA	Two to three morphologically distinct subspecies, *P. cocinna cocinna*, *P. cocinna floridana* (?), *P. cocinna suwanniensis*. (1)		[62]
*Pseudemys gorzugi*	mtDNA ND4, 5 μSats	? (small but stable refugium, not distinguishable)	Relatively homogeneous genetic structure throughout its range; (1)	old and recent	[124]
*Rhinoclemmys pulcherrima*	Morphology	NA	Four distinct subspecies, *R. pulcherrima pulcherrima, R. pulcherrima incisa, R. pulcherrima manni, R. pulcherrima rogerbarbouri*. Historic fluctuations in simulated potential distributions coincide with the distribution of subspecies. (1)	old	[62]
*Sternotherus minor*	mtDNA	Considerably lower genetic variability within the north-western clade (due to strong refugia retraction-expansion) (1)	Strong genetic split into west versus east lineage. (1)	old	[149]
*Sternotherus odoratus*	mtDNA	NA	Strong genetic split between west and east. (1)	old	[150]
*Terrapene carolina*	Morphology	NA	Significant morphologic splits between six subspecies: a western subspecies *T. carolina triunguis*, an eastern subspecies *T. carolina carolina*, *T. carolina bauri* on the Florida peninsula, *T. carolina major* near the gulf coast (but see below), and *T. carolina mexicana* as well as *T. carolina yucatana* in Mexico. Subspecies restrictions coincide with climate niche restriction over time in most subspecies (despite *T. carolina major*; *T. carolina carolina* and T. *carolina bauri* are not resolved). (1)	old	[151]
	mtDNA, d-loop; 9 μSats	No population structure in *T. c. major* (NA, likely not a distinct lineage)	Analyses comprised four of six extant subspecies of *Terrapene carolina* in eastern North America: *T. carolina bauri*, *T. carolina carolina*, *T. carolina triunguis*, and *T. carolina major*. Lineages are significantly differentiated, F_ST_ ranging between 0.12 (*carolina - bauri*) and 0.38 (*major - triunguis*). *T. carolina major* might not be a distinct evolutionary lineage. It is not predicted by the PPGMs. (1)	old	[152]
*Terrapene coahuila*	Mark-Release-Recapture; 9 μSats	NA	Isolation by distance across the geographical range suggests that dispersal limitation exists at the regional scale. (NA)	recent	[153]
*Terrapene nelsoni*	Morphology	NA	Significant morphologic split between a southern group (here *T. nelsoni nelsoni*) and a northern group (*T. nelsoni klauberi*). (1)	old	[151]
*Terrapene ornata*	Morphology	NA	Two subspecies, *T. ornate ornata* in the Mississippi basin south to Texas and *T. ornate luteola* within the Chihuahua, Coahuila, Sonora (Mexico) and Arizona, New Mexico and Texas (USA). (1)	old	[151]
	16 μSats	NA	Bottlenecked population show no loss of genetic information, Ne = 300, census pop size 700. (NA)	recent	[154]
*Terrapene ornata*	Morphology	NA	Significant morphologic split between a western subspecies (*T. ornata luteola*) and eastern subspecies (*Terrapene ornata ornata*). (x)	old	[151]
*Trachemys gaigeae*	Morphology	NA	Significant morphologic split between a northern group (*T. gaigae gaigeae*) and a southern group (*T. gaigae hartwegi*). (1)	old	[155]
*Trachemys nebulosa*	Morphology	NA	Significant split between the Baja California populations (*T. nebulosa nebulosa*) and populations on the eastern mainland (*T. nebulosa hiltoni*); (1)	old	[155]
*Trachemys scripta*	Morphology	NA	Significant morphologic split between an eastern subspecies *T. scripta scripta*, a western lineage (*T. scripta elegans*) and an intermediate northern lineage (*T. scripta troostii*). (1)	old	[155]
*Trachemys venusta*	Morphology	NA	Genetic subdivision into five subspecies, while one is restricted to a very small geographical area (thus not compared with modeling results). Subspecies restrictions coincide with climate niche restriction over time in *Trachemys venusta venusta*, *Trachemys venusta cataspila*, *Trachemys venusta panamensis*, and *Trachemys venusta uhrigi*. (1)		[156]

Given are details on intraspecific variability and differentiation, the marker system, the dating of splits (old = before the LGM, recent = after the LGM) as well as a comparison with the results obtained from paleophylogeographic modeling (1 = pattern mirrored in PPGM; x = pattern not mirrored).

To summarize, in this study we use modern geographic distributions to estimate realized and potential niches for modern turtle species. We use paleophylogeographic modeling to reconstruct the history of their geographic range changes over the last 320 Ka using (i) a temporally continuous set of paleoclimatic models that we constructed from paleoclimate general circulation model data, (ii) the oxygen isotope record of global mean annual temperature over the same period of time, and (iii) the phylogenetic changes in the niche that occurred along each species' branch as estimated from the phylogeny of the entire clade. We use fossil occurrences to test whether the modern realized niches are good predictors of paleogeographic distributions and to measure the frequency with which turtles lived in non-analogue climates in the past. We test whether the paleogeographic ranges are congruent with physiological and phylogeographic data and we determine whether evolutionary adaptation or geographic range tracking has been more important as species responded to the three glacial-interglacial cycles included in our study. We use our results to characterize the full potential niches of species and to identify ones that may be more vulnerable to the 21^st^ Century climate changes that are forecast for the next century.

## Materials and Methods

### Species occurrences

We analyzed 59 of the 61 chelonian species in North America. Although the phylogenetic relationships of these species are well-resolved and comparatively stable, their taxonomy is controversial, especially at the sub-species level [62], [65]–[67]. For consistency, we have arbitrarily chosen to follow the most recent taxonomic classification [62], but our modeling depends on the phylogeny rather than on the classification. Occurrences for most of the species were compiled from the World Turtle Database [60]. Adequate sample sizes are crucial for sound models [68]–[69], so for species with fewer than ten records in the World Turtle Database, we added occurrences from the Global Biodiversity Information Facility (GBIF, www.gbif.org). All occurrences were checked for accuracy. To avoid the effects of uneven sampling, we resampled the point occurrences with a grid with ten arc-minutes resolution, retaining only one occurrence point per grid cell. In total, 19,240 unique resampled points for the 59 species were retained for further analysis.

### Fossil occurrences

We compiled fossil occurrences of extant species for the last 320 Ka from published literature [61], [70]–[74] and the Paleobiology Database (pbdb.org). We did not include occurrences whose location or taxonomy was uncertain. In total 141 fossil occurrences were found for 18 of the 59 species (Appendix S3 in Material S1). The geological age estimates for these occurrences vary considerably in their precision and uncertainty. Most fossils younger than 50 Ka are radiocarbon dated, which provides precise ages accurate to within a few decades. Older fossils extend beyond the range of radiocarbon and are dated with lithostratigraphy and biostratigraphy. Occurrences between 50 Ka and 125 Ka are assigned to one of three Wisconsinan (last glacial) stages or to the Sangamonian interglacial (last interglacial), which have a precision of about 10 Ka. Older occurrences are assigned to one of two North American Land Mammal Ages (NALMA) and have precisions of tens of thousands of years [75]. The maximum and minimum age of each occurrence is reported in Appendix S3 in Material S1. Our strategy for dealing with the age uncertainty is described below.

### Modern climate data and paleoclimate interpolation

Modern climate data with a spatial resolution of 2.5 arc min were obtained from WorldClim [76]. This set of 19 bioclimatic variables characterize climate in terms of means (e.g. mean annual temperature) and extremes (e.g. maximum temperature of the coldest quarter) (see below; modified from [77]). We selected a subset of eight moderately to uncorrelated climate variables whose coefficients of determination were <0.75 to avoid the effects that correlations have on Maxent analyses (see below) [78]: mean diurnal temperature range (BIO2), maximum temperature of warmest month (BIO6), mean temperature of wettest quarter (BIO8), mean temperature of warmest quarter (BIO10), precipitation of wettest quarter (BIO16), precipitation of driest quarter (BIO17), precipitation of warmest quarter (BIO18) and precipitation of coldest quarter (BIO19).

We used a continuous temporal series of interpolated paleoclimate models previously developed for the late Quaternary of North America [23]. These interpolated models were constructed by using oxygen isotope data from deep sea cores [79], which are a proxy for global mean annual temperature, to interpolate between modern climate (an interglacial climate end-member) and a global circulation climate model (GCM) of paleoclimate at the last glacial maximum at 21 Ka (MIROC3.2 GCM, [80]) (a glacial climate end-member). Each end-member climate data set consisted of the same 19 bioclimatic variables described above. All the variables were scaled relative to their mean annual temperature using the continuous record of global mean annual temperature from the oxygen isotope record [23]. The interpolated paleoclimate models thus capture the full range of climate variability over the last 320 Ka, including the warmer climates of the last (Sangamonian) interglacial and the intense variability within glacial cycles. The paleoclimate models were constructed at 4 thousand year intervals, the same temporal resolution as the isotope data. The interpolated models were validated by comparing them with a GCM at 6 kya and a GCM that describes climate between 120 and 140 kya (see Figure 3 and Figure 4 in [23]). The interpolated models agreed well with the direct GCM models, differing no more from the GCMs than two GCMs differ from one another. Paleoclimate interpolation was performed at a resolution of 50 km^2^ equal-area grid cells to avoid latitudinal bias in sampling density [23]. The sampling grid coordinates are available at http://mypage.iu.edu/~pdpolly/Data.html.

**Figure 2:**
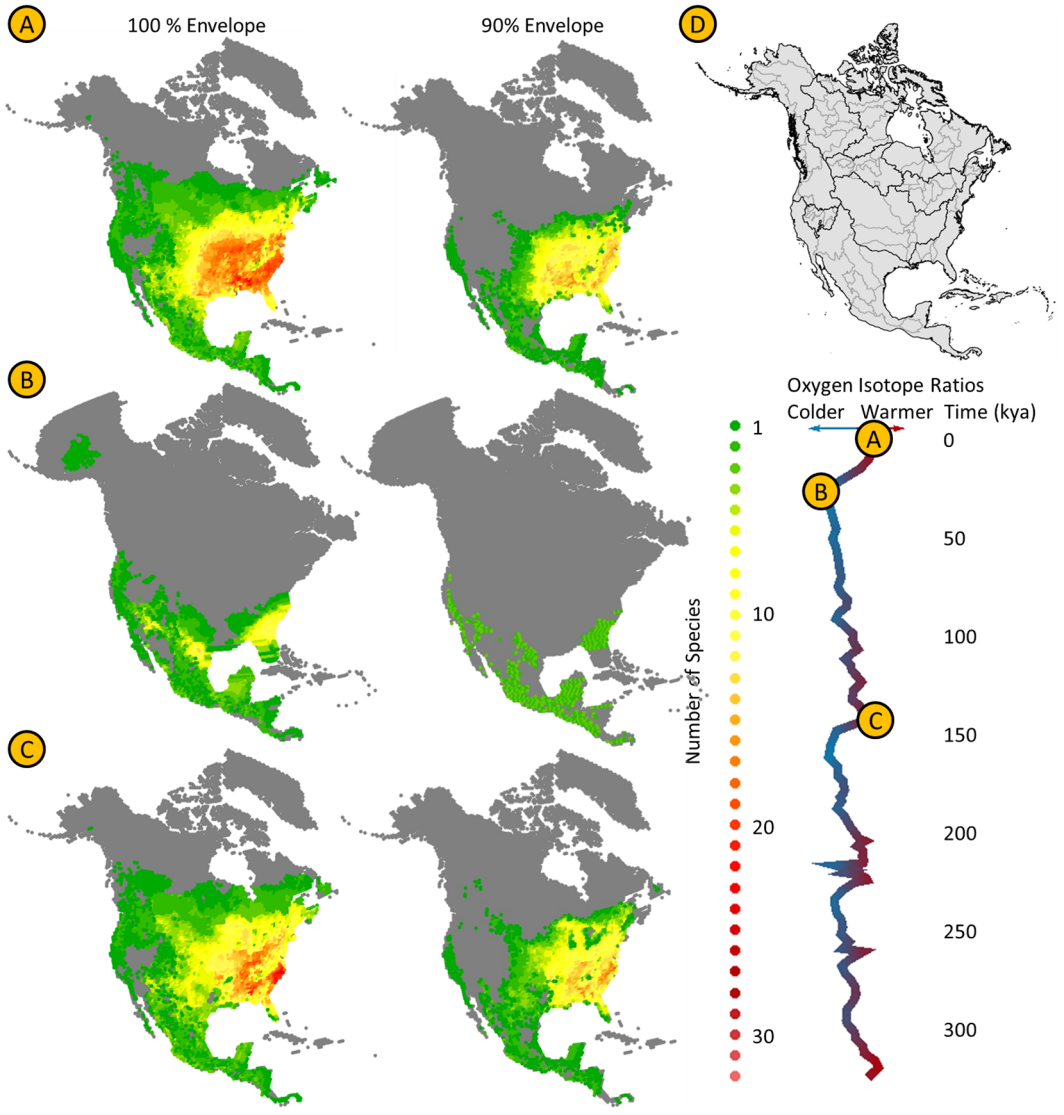
Predicted current species richness according to 100% and 90% environmental envelopes (A) as well as historic fluctuations as projected for the last glacial maximum 21 ky BP (B) and the last interglacial (C) according to palaeophylogeographic models of 59 Nearctic chelonians. Dispersal capacities per species were restricted to the corresponding watersheds (D). For full videos see Appendix S4 in Material S1.

**Figure 3:**
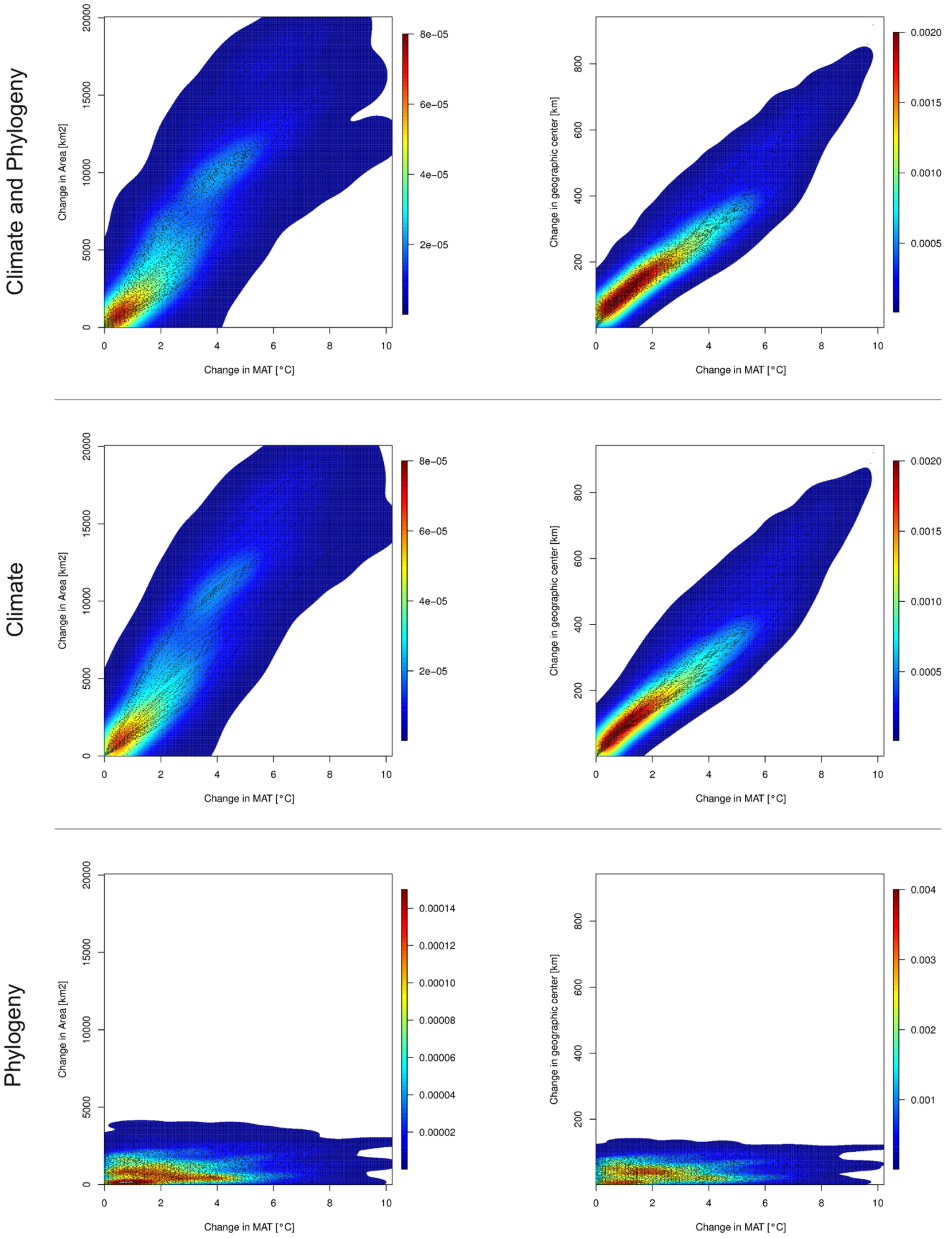
Relationships between pairwise changes in mean annual temperatures (MAT) and pairwise changes in potential distribution range sizes as well as between MAT and pairwise changes in geographic centers of potential distributions in 59 Nearctic chelonians during the last 320 ky. Warmer colors reflect higher point densities considering both climate and phylogenetic effects (top panel), only climate (middle panel) and only phylogeny (bottom).

**Figure 4:**
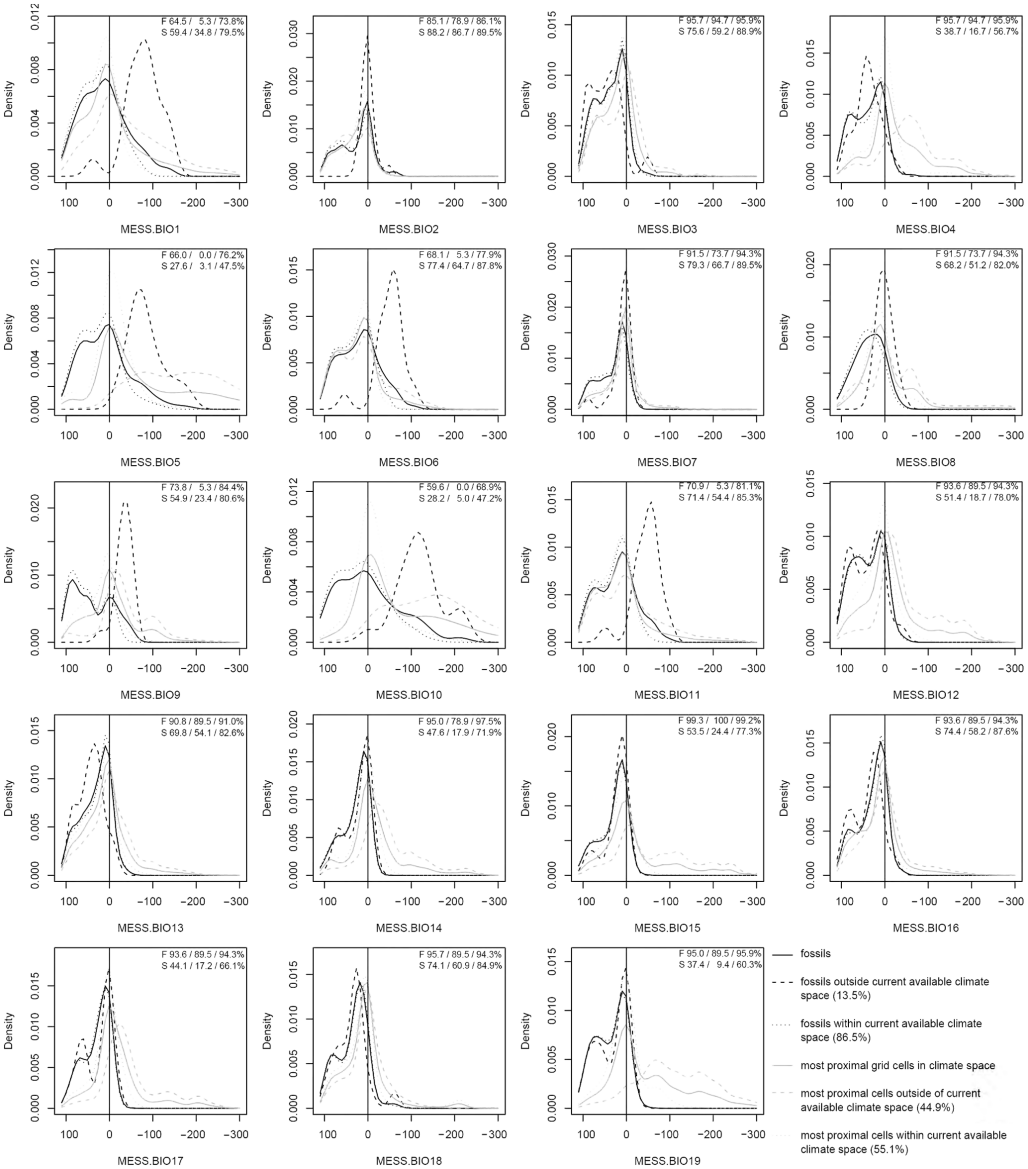
Historic niche dynamics in Nearctic chelonians based on 141 fossils and the five most proximal (SIM5) 50 km^2^ points in climate space in those cases where species find no analogous climates to their past potential niche. Positive MESS scores indicate conditions within the species' modern potential niche and negative MESS scores indicate bioclimatic conditions outside of the modern potential niche. Solid lines refer to the complete set of fossils (black) and SIM5 points (gray), wherein dotted lines refer to subsets nested across all variables within the species' potential niche. Percentages provided in each subplot refer to the proportion of records within the species' current realizable niche and the respective subsets (total/within potential niche/outside of potential niche) for fossils (F) and SIM5 (S). Dashed lines refer to subsets of exeeding those conditions currently available to the species in at least one predictor. Abbreviations are: BIO1 = annual mean temperature; BIO2 = mean diurnal range; BIO3 = isothermality; BIO4 = temperature seasonality; BIO5 = max temperature of warmest month; BIO6 = min temperature of coldest month; BIO7 = temperature annual range; BIO8 = mean temperature of wettest quarter; BIO9 = mean temperature of driest quarter; BIO10 = mean temperature of warmest quarter; BIO11 = mean temperature of coldest quarter; BIO12 = annual precipitation; BIO13 = precipitation of wettest month; BIO14 = precipitation of driest month; BIO15 = precipitation seasonality; BIO16 = precipitation of wettest quarter; BIO17 = precipitation of driest quarter; BIO18 = precipitation of warmest quarter; BIO19 = precipitation of coldest quarter; MESS: Multivariate Environmental Similarity Score.

### Species distribution models (SDMs)

Modern SDMs were produced for each species using Maxent 3.3.3e [51], [81]–[82]. Maxent provides good predictions of modern geographic ranges from point occurrence data [83]. Default settings were used with logistic output ranging from 0 (no probability of occurrence) to 1 (optimal) [82]. For model training, the available climate space was limited to the climate within the Level 2 watersheds where the species is known to occur (see below). 100 SDMs were computed for each species, each trained with a randomly selected 70% of the occurrences and evaluated with the remaining 30% of the occurrences. The models were tested using the AUC approach of Phillips *et al.* [81] where an AUC score of 0.5 indicates that the estimated range is not better than random and a score of 1.0 indicates perfect estimation. The 100 replicate SDMs were combined to create presence-absence maps using the minimum training presence logistic threshold and the ten-percentile training omission threshold methods as recommended by Liu *et al.* [84].

### Paleophylogeographic species distribution models (PPGMs)

To estimate geographic ranges of the species in the geological past, we used the paleophyogeographic approach described above, which incorporates phylogenetic correction for niche evolution and the continuous series of paleoclimatic models [23]. Potential niches were estimated from rectilinear BIOCLIM envelopes around modern E-space species occurrence points. The BIOCLIM approach is preferred for paleogeographic modeling because (i) it makes fewer assumptions about the constancy of the species-climate relationship than Maxent, despite the fact that it tends to over-predict ranges in the modern world (the over-prediction is because it tends not to identify range limits defined by biotic interactions) [18], [23], [49], [83]; and (ii) because its rectilinear shape has the potential to include non-analogue climates that are part of the fundamental niche, but which are not available in modern climate and thus not part of more restrictive niche envelopes. Importantly, rectilinear BIOCLIM climate envelopes are unbiased by correlations between climate variables, which is critical because the correlations between variables are not expected to be the same in the past as they are today. The full potential niche and one that excludes the outlying 10% of the E-space points were estimated. The suitability of the two estimates was assessed by their ability to correctly estimate the modern geographic distributions. The reduced envelopes were found to be too restrictive for many of the turtle species.

The modern potential niches were projected onto a phylogenetic tree of the 59 species (see below) to estimate the phylogenetic component of niche change (see [23] for complete description). Ancestral niches at the nodes of the tree and along its branches can be reconstructed from a generalized linear model (GLM) regression. The correlation structure we used in the GLM method assumes that the climatic niches evolved according to a Brownian motion at a stochastically constant rate. The regression was used to estimate the niche boundaries of each species at 4 ky intervals along their respective branches. These phylogenetically corrected potential niches were then used to estimate each species' paleogeographic range at each 4 ky interval. We refer to these estimated ranges as paleophylogeographic models (PPGMs). Each PPGM was masked using Level-1 watersheds from Pfafstetter System, which are large-scale river drainages like the Mississippi Basin (Figure 2D), in order to exclude spurious points in the reconstructed ranges that lie beyond the geographic barriers that make them inaccessible to the species. The 4 ky PPGM slices were compiled into an animated map that shows the changing potential range for each species through the last three glacial-interglacial cycles.

To partition the geographic responses of each species into adaptive and purely geographic components, we subtracted the phylogenetically corrected PPGMs from uncorrected PPGMs [23]. The phylogenetic (adaptive) component is the difference in geographic area and centroid between the two. The purely geographic component is the residual of the uncorrected PPGMs. The rate of geographic response per degree change in mean annual temperature (BIO1) was calculated as a function of total area of the geographic range and position of the range's geographic centroid using simple linear regression [23] and, for visualization, kernel density estimation using *kde2d* function of the *MASS* package for R 15.0 [85].

### Phylogeny

We constructed a time-calibrated molecular phylogeny for the 59 chelonian species using a supermatrix approach. Data from four mitochondrial genes (COI, control region, cytb, ND4) and three nuclear genes (R35, RAG-1, TB29) were downloaded from GenBank. The number of loci varied across the taxa, but even with missing data the concatenated data are still expected to provide a reasonable estimate of the phylogeny [86]. GenBank accession numbers for the sequences are reported in Appendix S1 in Material S1. Each gene was aligned separately using the MUSCLE algorithm (MUSCLE version 3.6, [87]) and checked manually. Ambiguously aligned or randomly similar sequence sections were detected with ALISCORE [88] and removed. The final, concatenated data set contained 5742 bp (including gaps). Parameters for the substitution model (GTR+G) were estimated separately for each gene by partitioning the data set during subsequent analyses. A Yule speciation process was used to estimate the prior probability of the tree. Divergence times were estimated with BEAST 1.6.2 with a relaxed molecular clock model with rates with an uncorrelated lognormal distribution [89]–[90]. Two fossil *Trachemys* species, *T. idahoensis* (4 Ma) and *T. inflata* (7 Ma) were used as calibration points for the most recent common ancestors (MRCA) of *T. scripta*/*T. gaigeae* and of the North American clade/Tropical clade of *Trachemys*, respectively [91]. *Glyptemys valentinensis* (14.5 Ma) was used to calibrate the MRCA of the genus *Glyptemys*; [92], and *Kinosternon pojoaque* (13.3 Ma) was used to calibrate the MRCA of *K. arizonense*, *K. durangoense* and *K. hirtipes*; [93]) For *T. idahoensis*, a uniform prior distribution with the lower bound at 4 Ma and the upper bound at 7 Ma (i.e. the lower bound of the older fossil species *T. inflata*) was used (cf. [92]). We used single-sided hard bounds for the minimum age of divergence and an exponential distribution as a soft bounds for the maximum age. Every 1000^th^ trees were sampled from three independent runs with 10^7^ generations. The initial 5% of each run was discarded as burn-in, resulting in a total of 28,500 trees. Maximum likelihood bootstrap values (1,000 replicates) were calculated using RAxML v.7.2.X [94].

### Dispersal limits and accessible climate

Drainage basin boundaries have been shown to be important barriers to turtle dispersal [66], [95]. Geographic data on basin boundaries were obtained from the USGS HYDRO1k dataset (eros.usgs.gov/#/Find_Data/Products_and_Data_Available/gtopo30/hydro/namerica). Level-2 drainage basin units were used as to define each species' available climate space for Maxent training [96] and for calculation of MESS scores (see below). Level-1 units (Figure 2D) were used to mask PPGMs. The masking allows us to incorporate the concept of accessibility into both the niche modeling and the geographic distribution modeling (see discussion in Introduction).

### Multivariate environmental similarity surface (MESS) scores

Multivariate environmental similarity surface (MESS) scores [51] were used to identify non-analogue paleoclimates and to describe the position of each species niche within its available climate space. Negative MESS scores identify past occurrences that fall outside the available climate space on one or more of the climate variables (i.e., non-analogue occurrences). Identification of non-analogue occurrences is important for distinguishing opening of potential niche space from evolution of the fundamental niche due to changing climate [97].

A MESS score is assigned to each point in the E-space based on where it falls with respect to the boundaries of a set of reference points. If the point falls within the boundaries, then a positive MESS score is assigned with a range from 0 to 100 depending on how close the point is to the center of the bounded area (a score of 100 indicates that the point lies in the middle of the bounded area). If the point lies outside the bounded area, then a negative MESS score is assigned with a range from 0 to -∞ as the Euclidean distance between it and the nearest boundary divided by the width of the bounded area. Thus if the distance between the point and the nearest boundary is equal to the width of the bounded area, then the MESS score is −100. For more details see Elith *et al.* [52].

Four kinds of MESS map were computed. A map of MESS scores was produced for each fossil occurrence with respect to (a) the boundaries of the species modern potential niche space and (b) for the boundaries of the combined available climate space of all the species using first the combined set of climate variables and then for each individual climate variable.

We used the MESS scores to deal with the uncertainty of the ages of the older fossils. We found the time slice in which the paleoclimate was the most compatible with the fossil occurrence as the one whose potential niche MESS score was the most positive (see below). Thus, we conservatively assumed that the best estimate of the age of the fossil is the time slice where the paleoclimate is most compatible with its geographic occurrence. Because we interpret negative MESS scores as indicators that potential climate space has been opened, this approach minimizes ad hoc assumptions that the fundamental niche has changed. Note that for two fossils we found two time slices with identical MESS scores so we included both slices as being equally likely.

For some time slices, the PPGMs of some species contracted to nothing, suggesting that the paleoclimate provided no suitable climatic habitat for the species and that it could easily have become extinct. We know with hindsight that the species are, in fact, still living, so for those species we arbitrarily assigned them a geographic range of the 5 equal-area grid cells (250 km^2^) whose potential niche MESS scores were the least negative (SIM5). We assumed that the SIM5 range was the most parsimonious refugium for the species (these arbitrarily assigned ranges are highlighted in the video montage maps of the PPGMs). We used potential niche MESS scores for the SIM5 as an estimate of how far a species can be pushed outside its modern climate limits and still survive.

We used the potential niche MESS scores computed with all climate variables and single variables for the past distributions relative to their modern potential niche in E-space as a quantitative measure of how much the potential niches have changed over the last 320 ky. We visualized the changes using the *sm.density.compare* function of the *sm* package [98] in R 15.0.

Alternative ways to measure niche change exist (e.g. [54], [99]–[101]). These include the amount of overlap between niches in E-space as a parameter that ranging from zero to one (e.g., Schoener's D), where zero means no overlap and one means complete overlap [100]–[101]. We preferred MESS scores because these alternatives do not provide information about the scale of difference between niches that do not overlap. Furthermore, they cannot be used to compare single point occurrences, e.g. fossils, to a niche distribution.

### Comparisons of paleophylogeographic models (PPGM) with molecular phylogeographic data

We compared our paleophylogeographic range maps to published phylogeographic scenarios derived from molecular and morphologic data for the 34 species with such data (the phylogeographic data are summarized in the left margin of the corresponding animated paleophylogeographic maps in Appendix S4 in Material S1). We compared the number of genetically distinct intraspecific phylogroups (based on molecular data) and their spatial distribution (e.g. phylogenetic lineages identified via allele frequencies, haplotypes and/or morphometric data) to the PPGM range maps to determine the concordance of these data with past range fragmentations and contractions. We used a simple binary scoring to summarize whether the molecular phylogeographic data were consistent (1) or inconsistent (X) with our paleophylogeographic maps in the number of genetically distinct phylogroups and the location of refugia. We chose to not statically quantify the geographic overlap between molecularly predicted refugia and our paleophylogeographic reconstructions [102] because of lack of geographic specificity of most phylogeographic data and because the conclusions we draw are sufficiently supported by our simple classification.

### Physiological data

Physiological parameters that might restrict the distribution of chelonians were compared to the modern SDM and Quaternary PPGM reconstructions to determine whether the realized niches in E-space have limits defined by the animals' physiology. Data on the following parameters were compiled from 226 field and lab experimental studies: maximum temperature tolerated by the species (critical thermal maximum, CT_max_), minimum temperature tolerated by the species (critical thermal minimum. CT_min_), and temperature range at which incubation occurs. We found data for 48 of the 59 species (Appendix S5 in Material S1). Maximum temperature of the warmest month (BIO5) was compared to CT_max_, the minimum temperature of the coldest month (BIO6) was compared to CT_min_, and the mean temperature of the warmest quarter (BIO10) was compared to incubation temperature range. Comparisons were made for each species' modern available climate space and its realized niche.

## Results

### Phylogeny

A single, well-supported tree resulted from the multi-gene Bayesian analysis (Appendix S6 in Material S1). All higher taxa (families, subfamilies and genera) involved were found to be monophyletic, except the genera *Sternotherus* and *Trachemys* (see discussion). The time since common ancestry of several genera were estimated to be very shallow (e.g. *Chrysemys*, *Graptemys* and *Pseudemys*) and most species are estimated to have originated in the last 2 my. The genus *Apalone* is estimated to have diverged from other taxa about 10 Ma.

### Modern SDM geographic range models

The Maxent SDM estimates of modern geographic ranges generally agreed well with the actual distributions (AUC values ranged from 0.740 to 0.972 with a mean 0.891 where 1.0 is perfect match and 0.5 is no better than random) (Appendix S7 in Material S1). All but two species, *Kinosternon durangoense* and *Apalone spinifer*, had modeled ranges that closely matched the real ranges with AUC>0.8. One of those, *K. durangoense*, a Mexican endemic, is very poorly studied and only a few occurrence points are known, thus contributing to uncertainty about its range. The other, *A. spinifera*, a very widespread species that occurs in many climates, has a broad general realized niche that is compatible with a much larger geographic range than it actually inhabits.

The climate variables that placed the strongest limits on the niches and thus the SDM ranges were mean temperature of warmest quarter (BIO10), minimum temperature of coldest month (BIO6), precipitation of driest quarter (BIO17), and precipitation of coldest quarter (BIO19). Most of the eight climate variables that we used for the SDMs were similarly relevant for each species, with standard deviations of there contributions ranging from 7.1 to 15.6% across species (a low standard deviation means that the variable has a similar effect on all the species). Nevertheless, the variation in how much some variables contribute to one species compared to another is high for some variables, indicating that the species differ in the variables that define the limits of their climate niches. For example, precipitation in the coldest quarter, BIO19, whose contribution ranges from 0.1% in *Pseudomys alabamensis*, a species with a tiny distribution along a small segment of the Gulf Coast, to 76% in *Graptemys oculifera*, a species that also has a small distribution but one that extends northward into the continental interior in Alabama.

All Maxent results are provided in Appendix S7 in Material S1. Maps showing the SDM geographic range models for each species are provided in Appendix S4 in Material S1.

### Paleophylogeographic models of Quaternary geographic ranges (PPGMs)

Paleophylogeographic models (PPGMs) are presented as animated maps in Appendix S4 in Material S1 to show the modeled changing geographic ranges of each of the 59 species through the last three glacial-interglacial cycles (320 Ka). Each map shows the PPGM models of the expansions, contractions, and fragmentation of the paleogeographic range along with the fossil occurrences, molecular phylogeographic data, and map of parts of the continent with non-analogue climates. The paleogeographic range models take into account evolutionary changes in the species' climatic niche as estimated from the phylogenetic tree presented above. Each animated map consists of 80 frames separated by 4 ky intervals. The global oxygen isotope curve is shown to the right of each map with a moving index marker to show the age of each frame. These animated maps are key to understanding our conclusions.

Summary maps showing species diversity at three key times are shown in Figure 2. Modern species diversity calculated from the SDM geographic range models is shown in 2A. Paleo species diversity calculated from the PPGM paleogeographic range models is shown in 2B for the last glacial maximum (20 Ka) and in 2C for the last interglacial maximum (140 Ka). The regions that are today covered by temperate forests, temperate grasslands, deserts, and lake systems were regions where geographic ranges changed the most over the last 320 ky. In contrast, the regions along the Pacific Coast, the mixed mountain highland biomes, and the tropics were the regions where the ranges changed the least (Appendix S4 in Material S1, Figure 2).

There were strong relationships between change in mean annual temperature (PC_MAT_) and change of the geographic centers of species' ranges (PC_GC_) (*R^2^* = 0.904, p<0.0001, PC_GC_ = 28.66+73.17*PC_MAT_) and the total areas of their geographic range (PC_RS_) (*R^2^* = 0.796, p<0.0001, PC_RS_ = 24.50+2135.68*PC_MAT_) based on the PPGM models (Figure 3). In other words, the average geographic range of a species expanded or contracted by more than 2,000 km^2^ for every degree that the mean annual temperature increased or decreased. When the phylogenetic correction was omitted, the relationship between change mean annual temperature and purely climate driven change in the geographic center of the range was about the same (*R^2^* = 0.904, p<0.0001, PC_GC_ = 8.32+78.74*PC_MAT_) and the relationship with change in area was stronger (*R^2^* = 0.837, p<0.0001, PC_RS_ = −42.09+2323.9*PC_MAT_) indicating that adaptation has contributed by only a small amount to the responses to climate change over the last 320 Ka. This conclusion was confirmed by modeling the purely phylogenetic component of change, which had no significant relationship between PC_MAT_ and PC_GC_ (*R^2^* = 0.016, p>0.05) or between PC_MAT_ and PC_RS_ (*R^2^* = 0.019, p>0.05). Geographic tracking has been by far the dominant mechanism by which chelonians have responded to changing climates over the last 320 Ka. The pace of phylogenetic diversification of their niches has been far slower than the 100 ky glacial-interglacial cycles of the Quaternary.

The greatest contraction of geographic ranges in PPGMs occurred during glacial periods. In fact, there were two or more 4-ky time slices when there was no suitable climate for 33 species: min = 2 slices for *Pseudemys gorzugi*; max = 96 slices for *Graptemys pearlensis*; median = 45 slices for the 33 species (see Appendix S4 in Material S1). Most of the species that had such extreme geographic bottlenecks in our models have very small modern ranges or are found close to the Gulf of Mexico.

### Paleogeographic ranges compared to phylogeographic data and fossil occurrences

One of the main goals of this study was to determine whether the potential climatic niches of North American turtles are adequate proxies for their fundamental niche by testing how well paleogeographic ranges derived from them agree with phylogeographic data and fossil occurrences. Our paleophylogeographic range models were usually successful at predicting modern patterns of within-species genetic differentiation. The patterns of range fragmentation during glacial cycles corresponded to the spatial distribution of living subspecies (11 out of 14 cases) and an even higher proportion of the fragmentation patterns corresponded to the modern distribution of genetically distinct phylogeographic lineages (21 of 23 species) (Table 1, Appendix S4 in Material S1). Species with deep genetic splits between subspecies or populations often experienced long periods of climatically driven range fragmentation, in many cases cyclically repeated allopatric separation (e.g., *Apalone mutica* [103], *A. spinifera* [104], *Kinosternon subrubrum* [105]), whereas species whose past ranges were not fragmented by climate cycles show little evidence of genetic differentiation (e.g. *Actinemys marmorata* [106]; Table 1, Appendix S4 in Material S1). In rare cases, species with marked range fragmentation in the past have little genetic structure today (e.g. *Chelydra serpentina* [106]). Species whose paleogeographic ranges contracted to nothing or nearly nothing almost always have very low genetic diversity and small geographic ranges today, suggesting that they experienced climate-driven geographic bottlenecks in the past (e.g., *Chrysemys dorsalis* [107], *Glyptemys insculpta* [108], *Glyptemys muhlenbergii* [109], *Graptemys gibbonsi* [110]; Table 1, Appendix S4 in Material S1). Morphologic data generally corresponded with these results. Intraspecific morphological diversities were smallest in those species with small distributions in the present or past or where large range changes likely caused bottleneck effects.

Most of the fossils fell within their potential niches (87%), indicating strong niche stability (Table 2). E-space plots of the fossils, the available climate, the realized niches, and the potential niches for each species are shown in Appendix S2 in Material S1. Of the 13% that fell outside the modern available niche space, most did so on mean temperature of the warmest quarter (BIO10; 66.7%) and mean diurnal temperature range (BIO2; 9.7%) (Table 3, Figure 4, Appendix S3 in Material S1). In all, fossils were outliers on only seven of the 19 bioclimatic variables (BIO1, 2, 5, 6, 9, 10, 11). We interpreted this to mean that niches were conserved for the other 12 bioclimatic variables, and we interpret the 87% agreement between fossils and potential niches to mean that niche conservatism is strong (after taking into account phylogenetic evolution).

**Table 2:** Summary statistics for fossil occurrences in Nearctic chelonians in terms of availability and niche position relative to the species' modern niches (for more details see Appendix S3 in Material S1).

Species	N fossils	within realized niche [%]	within potential niche [%]
*Actinemys marmorata*	4	100.0	100.0
*Apalone ferox*	2	0.0	100.0
*Apalone spinifera*	6	16.7	66.7
*Chelydra serpentina*	22	63.6	77.3
*Chrysemys picta*	14	42.9	92.9
*Clemmys guttata*	1	100.0	100.0
*Deirochelys reticularia*	4	25.0	100.0
*Emydoidea blandingii*	7	42.9	85.7
*Kinosternon subrubrum*	3	66.7	100.0
*Malaclemys terrapin*	1	100.0	100.0
*Pseudemys concinna*	5	20.0	80.0
*Pseudemys floridana*	3	33.3	100.0
*Pseudemys nelsoni*	3	33.3	100.0
*Sternotherus minor*	1	100.0	100.0
*Sternotherus odoratus*	4	50.0	75.0
*Terrapene carolina*	41	56.1	87.8
*Terrapene ornata*	5	60.0	100.0
*Trachemys scripta*	15	26.7	80.0
Total	141	48.9	86.5

**Table 3:** Multivariate environmental similarity scores (MESS) for fossils that fell outside the potential distribution of their species. Negative scores indicate the distance outside the potential niche as a percentage of the niche's size.

Subsets	Variable	N	Mean MESS	Max MESS
Total	BIO10	48	−79.6	−218.0
	BIO2	7	−26.4	−61.6
	BIO5	5	−45.7	−85.4
	BIO8	4	−206.0	−814.3
	BIO4	3	−27.2	−58.7
	BIO6	1	−1.6	−1.6
	BIO17	1	−7.9	−7.9
	BIO13	1	−25.3	−25.3
	BIO12	1	−7.5	−7.5
	BIO19	1	−16.4	−16.4
outside modern potential niche				
	BIO10	18	−125.5	−218.0
	BIO5	1	−24.0	−24.0
within modern potential niche				
	BIO10	30	−52.1	−149.2
	BIO2	7	−26.4	−61.6
	BIO5	4	−51.1	−85.4
	BIO8	4	−206.0	−814.3
	BIO4	3	−27.2	−58.7
	BIO6	1	−1.6	−1.6
	BIO17	1	−7.9	−7.9
	BIO13	1	−25.3	−25.3
	BIO12	1	−7.5	−7.5
	BIO19	1	−16.4	−16.4

For example, a MESS score of −1.6 on BIO6 indicates that the fossil fell 1.6% outside the niche's breadth on the BIO6 climate variable (c.f., Figure 1). Only incompatible climate variables are reported here (see Appendix S3 in Material S1 for a full summary).

### Physiological constraints on species ranges and niches

Physiological data were generally congruent with potential niches. The maximum temperature that a species is able to tolerate (CT_max_) was greater than the maximum warmest temperature (BIO5) in the past and present potential niches of 100% of the species for which CT_max_ was known (27 out of 59 species [111]–[114]). *Chrysemys picta* and *Actinemys marmorata* were the only species where the maximum temperature of their potential niche came to the limits of CT_max_ (Appendices S2 and S5 in Material S1). This suggests that most turtle species can tolerate higher maximum temperatures than they have been subjected to in the present or past. Incubation temperature range was also generally congruent with past and present potential niches. Eight species with fossils had incubation temperature data. Of those only one had incubation ranges that were incongruent with its potential niche.

Conversely, the minimum temperature that a species can tolerate (CT_min_) was strongly incongruent with the potential niche data. CT_min_ data and fossil data were available for eight out of 59 species, seven of which had potential niche minimum temperatures (BIO6) that were lower in the past or present than CT_min_, suggesting either that the species were able to behaviorally insulate themselves from winter colds or that CT_min_ has changed over time, or that the potential niches in the past were limited by CT_min_.

## Discussion

Our paleophylogeographic models illustrate the dramatic range changes that most turtles have experienced during the last three glacial-interglacial cycles (Appendix S4 in Material S1). The geographic ranges of most species fragmented and contracted into one or more southern refugia, often in peninsular Florida, coastal Texas and northern Mexico. Most of the Mexican and Central American species experienced very little change in the geographic centers of their ranges, but the areas of their ranges often contracted. Some local populations in these southern species may have persisted for geologically long time periods in the same place, which is not the case for the northern species. The PPGM projections agree well with molecular phylogeographic patterns (82.4% of 17 species are congruent), genetic subgroupings (91.3% of 23 species are congruent), and subspecies-level differentiation (78.6% of 14 species are congruent; Table 1). Genetic diversities are highest in species for which our PPGM models indicate either stable or large potential distributions, while low genetic diversities were mostly evident in species for which our PPGM maps show significant range changes or severely restricted distributions. Fossil occurrences are generally congruent with the geographic ranges and potential niche reconstructions of the PPGMs, which suggests that climate tolerances of these species have not changed substantially over the last 320 Ka. However, there are exceptions that do indicate some niche changes (Table 2).

### Phylogeny

Our phylogenetic tree is largely congruent with previous phylogenetic work (for a synopsis see Iverson *et al.* [115]). Most discrepancies were found within the Kinosternidae and some genera of the Deirochelinae. Regarding the first, the position of *Sternotherus depressus* leads to a paraphyly of *Sternotherus* in respect to *Kinosternon*. This might be an artifact of the sparse and often non-homologous data available for the members of these two genera and is also reflected by generally low support values in this clade. However, there is currently no comprehensive molecular phylogeny of this group available for verification. Although intrageneric relationships of *Graptemys* and *Pseudemys* in our results slightly differ to previous hypotheses, the posterior probabilities strongly support our topology (Appendix S6 in Material S1). The major ambiguity is the polyphyly of *Trachemys* due to *T. nebulosa*, which is the sister species of *Malaclemys* according to our results, yet this has poor support.

### Paleophylogeographic reconstructions agree with molecular phylogeography and subspecies taxonomy

Our animated paleophylogeographic range maps (Appendix S4 in Material S1) are strongly congruent with biogeographic patterns inferred from population genetic studies and phylogenies. The number and location of past refugia in our models mostly agree with reconstructions derived from population genetic and phylogeographic analyses. Refugia in the PPGMs correspond to deep genetic splits (e.g., subspecies) in most species (c.f., *Apalone mutica*, *Chrysemys picta*, *Trachemys scripta*, *Rhinoclemmys pulcherrima*, *Terrapene nelson*, *Trachemys nebulosa* and *Trachemys venusta*), though minor geographic entities were sometimes less well resolved via PPGMs (c.f., *Macrochelys temminckii* and *Malaclemys terrapin*). Our modeling exercises suggest that several species had no suitable climate space for at least some period during the past three interglacials. This is unlikely to be a methodological artifact resulting from masking the PPGMs by watershed, because most of the Level-1 watersheds are oriented such that a species could track climate for very long north and south distances.

It is instructive to look at the details of species for which there appears to have been no suitable climate during Pleistocene cold phases because the details of each species are different [116]. The freshwater emydid *Emydoidea blandingii* is one. This species inhabits the Missouri river system as well as rivers along the Upper Mississippi catchment, meaning that the entire Mississippi basin was treated as available space in our models. The species should be easily able to track its niche southward, if suitable climate space had been available. Our SIM5 points (the five 50 km^2^ points that are climatically most compatible with the potential niche of the species) suggest three main areas where this species may have found refugia that were close to the climate it is known to tolerate: on the great plains of Kansas and Nebraska, in the Midwest south of the Great Lakes, or on the mid-Atlantic seaboard (Appendix S4 in Material S1). A fossil occurrence in the Great Plains confirms the first of these as a possibility, and a genetic study by Mockford *et al.* [117] is also consistent with this picture. Using mtDNA, these authors argued for two past barriers of gene flow that match the breaks in our PPGM models.

*Glyptemys insculpta*, a northerly distributed semi-freshwater species is another example. Amato *et al.* [108] conducted range-wide genetic analyses on this species and detected very weak differentiation based on mitochondrial genes. The authors suggest a combination of the origin of a single glacial refugium, as well as a selective sweep of the mtDNA genome as the most likely explanation for their findings. A selective sweep was supported through a comparative study finding higher differentiation in microsatellites [118] leading to a mismatch in genetic structuring between the two marker systems. Our PPGM models of *G. insculpta's* geographic history suggest the species had two refugia separated by the Appalachians. Fossil occurrences confirm the presence of a population during the Late Pleistocene – Early Holocene in the east of the Appalachians around the Alabama/Georgia region (cf. Amato *et al.* [108] and references therein). High differentiation of populations suggested by microsatellite analyses [118] makes the existence of further refugia in the west likely. Increased selective pressure within the mtDNA could lead to a dominant genotype of the eastern refugial clade, leading to a selective sweep also in individuals originated from the western refugium after postglacial expansion and secondary contact.

A similar picture becomes evident when looking on southerly distributed species, but for different reasons. In contrast to northerly distributed species, the Gulf of Mexico represents an absolute barrier for southward movements in species with ranges adjacent to the Gulf coast. Several species – especially those with very small range extents such as some *Graptemys* species – may have found non analogous climate space during the past within their restricted geographic distributions. Because all species studied here have survived the climatic variations during the past, there are two main explanations: (1) species have undergone shifts in their realized niche which would give insights into the dynamics of their respective fundamental niche, or alternatively (2) microhabitat features not covered by the comparatively coarse grained resolution of our modeling act as microrefugia as proposed by and Rull [120], which remain unconsidered in our framework. The latter, for example, may be an explanation for the discrepancy between the distribution of currently observed genetic structure and PPGM results in *Macrochelys temminckii*. An analytical comparison of both genetic analyses and PPGMs would shed light into this issue.

### The history of intraspecific diversity

One major implication of the paleophylogeographic approach is that we can disentangle the geographic history of species that have experienced loss of genetic information due to genetic drift. Genetic drift (*sensu* Wright [121]) is a phenomenon that can lead to positive or negative impacts on intraspecific diversity, in which genetic information can get lost and/or recessive information (alleles or haplotypes) can become dominant. Intraspecific (genetic or morphologic) variability differs strongly among species as well as within populations of each species and highly depends on the species' history. In the field of biogeography, we have to distinguish two main processes severely influencing the intraspecific variability: (i) the positive impact of genetic drift may be the consequence of long-lasting geographic isolation and long-term restriction to geographically small retreats, and (ii) a negative impact (like losses of genetic information) in the wake of repeated expansion-retraction dynamics over large geographical space.

When comparing the potential distribution projections from SDMs and PPGMs with molecular data, we find congruencies between range shifts during the past with species' intraspecific diversities in the majority of the analyzed cases. Low genetic diversities are detectable in those species showing the most extensive range contractions. Small and isolated refugia often lead to small population sizes. Individuals from such populations suffer much stronger under environmental stochasticity, population fluctuations and thus repeated population bottlenecks [122]. Genetic data reveal strong population bottlenecks for various turtle and tortoise species from the southern US and Mexico [104], [123]–[124], except for species where different refugial ranges became evident during modeling (e.g. [104], [106], [125], but see [126]). For example, SDMs obtained for *Emydoidea blandingii*, *Glyptemys insculpta* and *Glyptemys muhlenbergii* highlight strongly restricted and small refugia over a long time period and,molecular analyses reveal a negative impact on intraspecific diversity. In contrast, SDMs indicate large and rather stable refugia for *Apalone spinifera*, which might had consequences on the survival of population networks and led to the maintenance of high genetic diversities over time, as revealed by molecular data [105]. The second negative effect of genetic drift can be caused by repeated expansion-retraction dynamics of a species' distributional area in the wake of tracking its climatic niche. These range shifts (e.g. the (re)colonization of (new) suitable habitats) may imply severe population bottlenecks in the wake of small founder populations and in consequence might lead to the elimination of alleles [3], [127]–[128]. Species with restricted dispersal (e.g. stepping-stone wise movement) are more affected by this phenomenon than species showing a phalanx-wise migration mode (see [3]). Interestingly, several species' PPGM ranges dwindle to nothing during cooling events (i.e., glacial periods) in the PPGM framework. This highlights the species-specific niche dynamics or, alternatively, scale effects not covered by our framework such as the presence of microrefugia [119], [120].

### Nearctic chelonian thermal biology

Correspondence between physiological variables and the potential niche described by SDMs and PPGMs vary, where CT_max_ is congruent and CT_min_ is incongruent. The potential niches of all species compared are in congruence with CT_max_. This suggests CT_max_ is represented accurately in the SDMs and that it is a relatively conserved variable. On the other hand, CT_min_ is incongruent with the potential niches. The comparison between the minimum temperature of the coldest month (BIO6) with CT_min_ was intended to explain the extreme surface temperatures during hibernation within the modern potential range. However, many Nearctic chelonians are aquatic and aquatic species usually spend their hibernation period submerged in water at 4°C (e.g. [129]). This hibernation strategy enables the habitation of environments that only temporarily provide suitable conditions. In addition, hatchlings of numerous species are reported to hibernate within the nest and withstand temperatures well below the critical thermal minima reported for adults (e.g. [129]).

### Niche dynamics revealed by fossils and SIM5

Analyzing the relative niche position of turtle fossils unexpectedly reveals that the fossils that deviate from the species potential niche do so mostly in mean temperature of the warmest quarter (BIO10). Within the deviant fossils and their associated species, this suggests large niche shifts of up to one niche breadth in multivariate MESS analyses using the comprehensive set of 19 bioclimatic variables, as well as in univariate MESS analyses (Figure 4). Comparing our fossil and SIM5 data with our PPGM ranges, we detected a multidimensional niche shift in high proportions of the species (Figure 2, Tables 2, 3). Notably, the mean temperature of the warmest quarter (BIO 10) shows a much higher plasticity of pivotal incubation temperatures than previously assumed. This result is somewhat unexpected as the majority (42 of 59 species are documented in the literature) of the Nearctic chelonians have temperature-dependent sex determination (TSD), in which ambient temperature during egg incubation in the summer months determines sex, and only a few (four species have documented cases) have genotypic sex determination (GSD) (see Appendix S5 in Material S1). In TSD species, the sex is environmentally determined wherein, depending on the TSD mode, a balanced sex ratio among hatchlings is only possible within a relatively narrow temperature range [64], [130]. Incubation temperatures exceeding this range in either direction, colder or warmer, may cause the development of only one sex and ultimately skewed sex ratios in populations. In previous studies, authors assume that sex-determining mechanisms may strongly affect the survival probability of a species in a geological time frame [131]. Recently, there is a considerable debate about how the impact of human-induced climate changes might affect species with TSD [132]–[133], and some studies underline that climate change might jeopardize sex ratios and therefore cause severe population decline and extinction [134]. On the other hand, Silber *et al.* [135] provided evidence that a higher proportion of TSD species compared with GSD species may have survived historic climate fluctuations suggesting some advantages of TSD over GSD, wherein Valenzuela & Adams [136] provided evidence that chelonians might have adapted to prehistoric climate change by shifting from TSD to GSD. These results suggest that the species' niches may be much more flexible than previously assumed.

### Revolution in the application of distribution models

The paleophylogeographic approach can be of high relevance in understanding the Quaternary background of species, including past range shifts with subsequent differentiation (e.g. the evolution of Evolutionary Significant Units, [137]) and genetic bottlenecks. All of our comparisons with PPGMs indicate strong congruencies for most species analyzed here for physiological, morphologic and molecular characters. Hereby, the data show strong overlap in past differentiation processes among/within taxa and the evolution of genetic diversity, as well as novel insights into past niche dynamics. These results underline the applicability of PPGMs as a valuable tool to model past range shifts and to picture potential processes standing in concert with long-term isolation and severe range contraction (e.g. population fluctuations and subsequent bottlenecks). Thus, species distribution models have the potential to shift from classical biogeographic range-shift analyses (from past to future) to conservation biology and the characterization of species, and could be a useful tool to predict intraspecific structures (such as differentiation, lineages and diversities) - without expensive molecular analyses. In regard to the 59 turtles and tortoise species, PPGMs are especially of high relevance since the modern distributions for these taxa will turn into non-analogous climatic conditions within the next few decades [59], [138]–[139], which might ultimately lead to cryptic biodiversity loss as suggested for numerous taxa [140].

## Supporting Information

Material S1 **Zip file containing Appendices S1 through S7. Appendix S1:** Genbank accession numbers of sequences of four mitochondrial genes (COI, control region, cytb, ND4) and three nuclear genes (R35, RAG-1, TB29) used for phylogenetic analyses; **Appendix S2:** Plots showing the bioclimatic conditions within each species' currently available climate space, its potential niche as well as its realized niche both in terms of species' modern and fossil occurrences; **Appendix S3:** Fossils of Nearctic chelonians compiled from Holman [61] and supplemented with data obtained through the Paleo Biology Database (available through www.paleodb.org). Negative MESS scores indicate conditions within the species' current realized/potential niche and positive MESS scores indicate bioclimatic conditions outside of the current realized/potential niche; **Appendix S4:** Videos showing the results of the Maxent and PPGM analyses, i.e. distribution changes through time in comparison to current distributions according to Maxent, non-analogous climate and information on subspecies and phylogenetic lineages; **Appendix S5:** Comprehensive summary of data on thermal biology of 48 Nearctic chelonian species; **Appendix S6:** Dated molecular phylogeny for all 59 extant species of Nearctic turtles inferred by Bayesian analyses; **Appendix S7:** Summary statistics of each 100 Maxent models per species.
(ZIP)
