# Supplementary material for: Evaluating the Significance of Paleophylogeographic Species Distribution Models in Reconstructing Quaternary Range-Shifts of Nearctic Chelonians
Source: PLoS One. 2013 Oct 9;8(10):e72855. doi: 10.1371/journal.pone.0072855 (PMC3794015; doi:10.1371/journal.pone.0072855)
Supplement: Material S1 — Zip file containing Appendices S1 through S7. Appendix S1: Genbank accession numbers of sequences of four mitochondrial genes (COI, control region, cytb, ND4) and three nuclear genes (R35, RAG-1, TB29) used for phylogenetic analyses; Appendix S2: Plots showing the bioclimatic conditions within each species' currently available climate space, its potential niche as well as its realized niche both in terms of species' modern and fossil occurrences; Appendix S3: Fossils of Nearctic chelonians compiled from Holman [61] and supplemented with data obtained through the Paleo Biology Database (available through www.paleodb.org). Negative MESS scores indicate conditions within the species' current realized/potential niche and positive MESS scores indicate bioclimatic conditions outside of the current realized/potential niche; Appendix S4: Videos showing the results of the Maxent and PPGM analyses, i.e. distribution changes through time in comparison to current distributions according to Maxent, non-analogous climate and information on subspecies and phylogenetic lineages; Appendix S5: Comprehensive summary of data on thermal biology of 48 Nearctic chelonian species; Appendix S6: Dated molecular phylogeny for all 59 extant species of Nearctic turtles inferred by Bayesian analyses; Appendix S7: Summary statistics of each 100 Maxent models per species. (ZIP) [file pone.0072855.s001.zip › renamed_91f67/Appendix S2 Rödder et al. - PLoS ONE.pdf]

Appendix S2

Rödder, D., A. M. Lawing, M. Flecks, F. Ahmadzadeh, J. Dambach, J. O. Engler, J. C. Habel, T. Hartmann, D. Hörnes, F. Ihlow, K. Schidelko, D. Stiels, P. D. Polly. Evaluating the Significance of Paleophylogeographic Species Distribution Models in Reconstructing Quaternary Range-shifts of Nearctic Chelonians. PLoS ONE X: XXXX

Plots in E-space of each species showing available climate (light grey), realized niche (dark grey), potential niche (medium grey), and climate associated with fossil occurrences (black, red, green, blue, etc.). Available climate is the climate occurring at points in the level-2 drainage basins occupied by the species. Realized niche points are the climate points associated with the species occurrences in the modern world. Potential niche is the total climate space occupied by the species in the PPGM models. For species with fossils, each fossil occurrence is shown in one of the other colors. For fossils whose age was uncertain, points of the same color show the paleoclimate associated with the fossil occurrence at each time slice at which the fossil could conceivably occurred. For further explanation, see Figure 1 and associated Introduction text.

sp1 − Actinemys marmorata

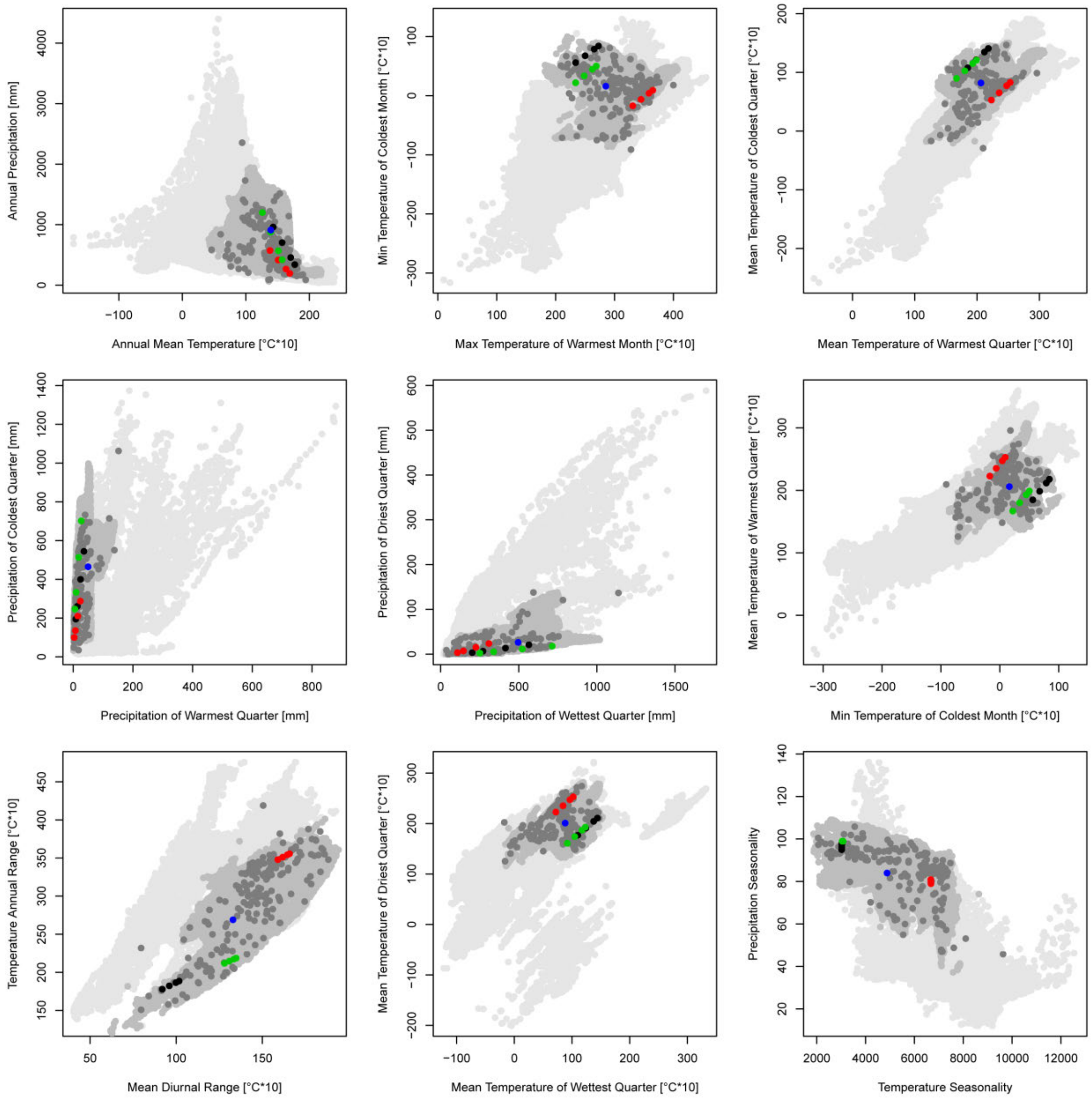

number of samples: 213

number of fossils: 4

sp2 − Apalone ferox

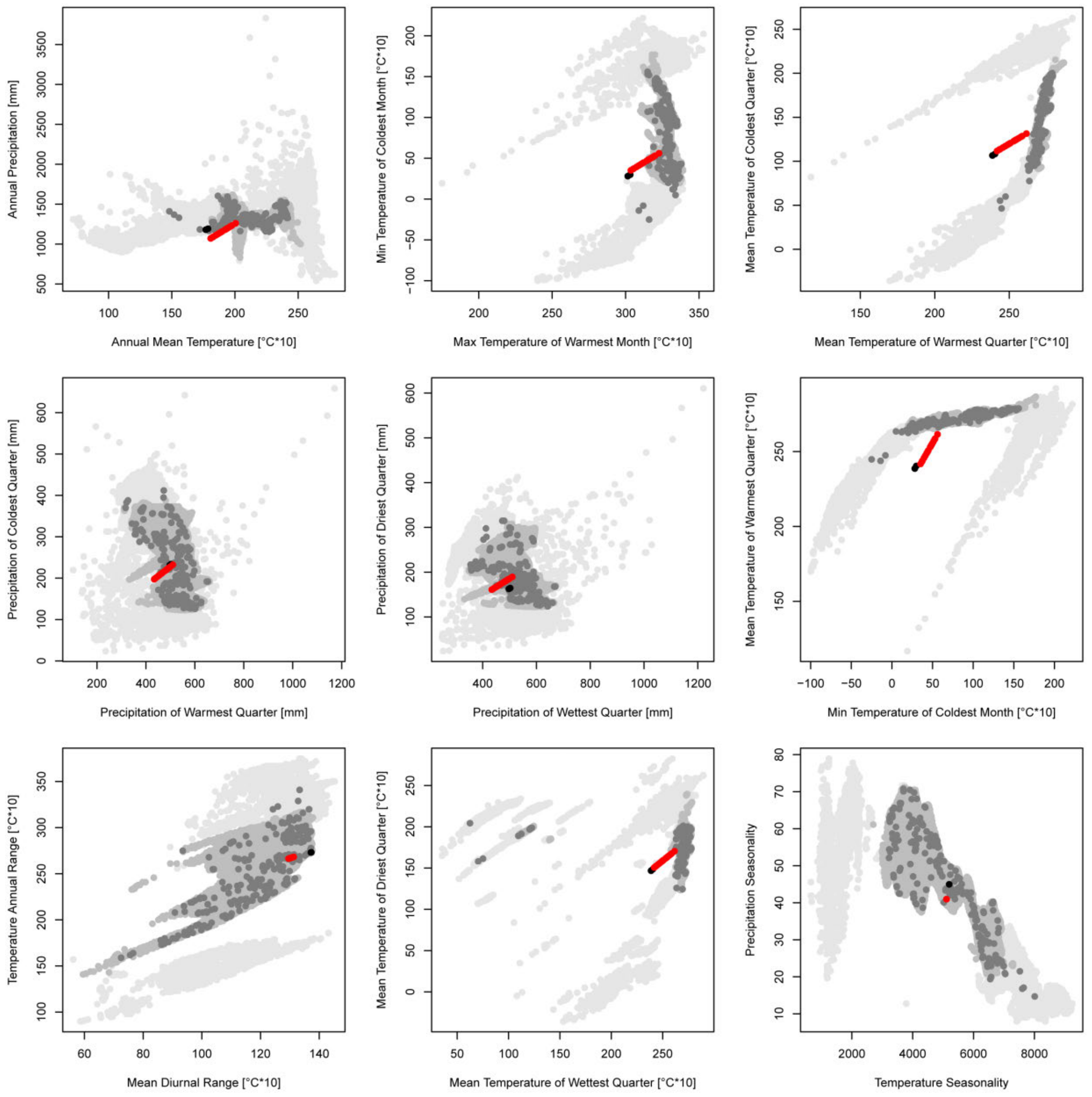

number of samples: 265

number of fossils: 2

sp3 − Apalone mutica

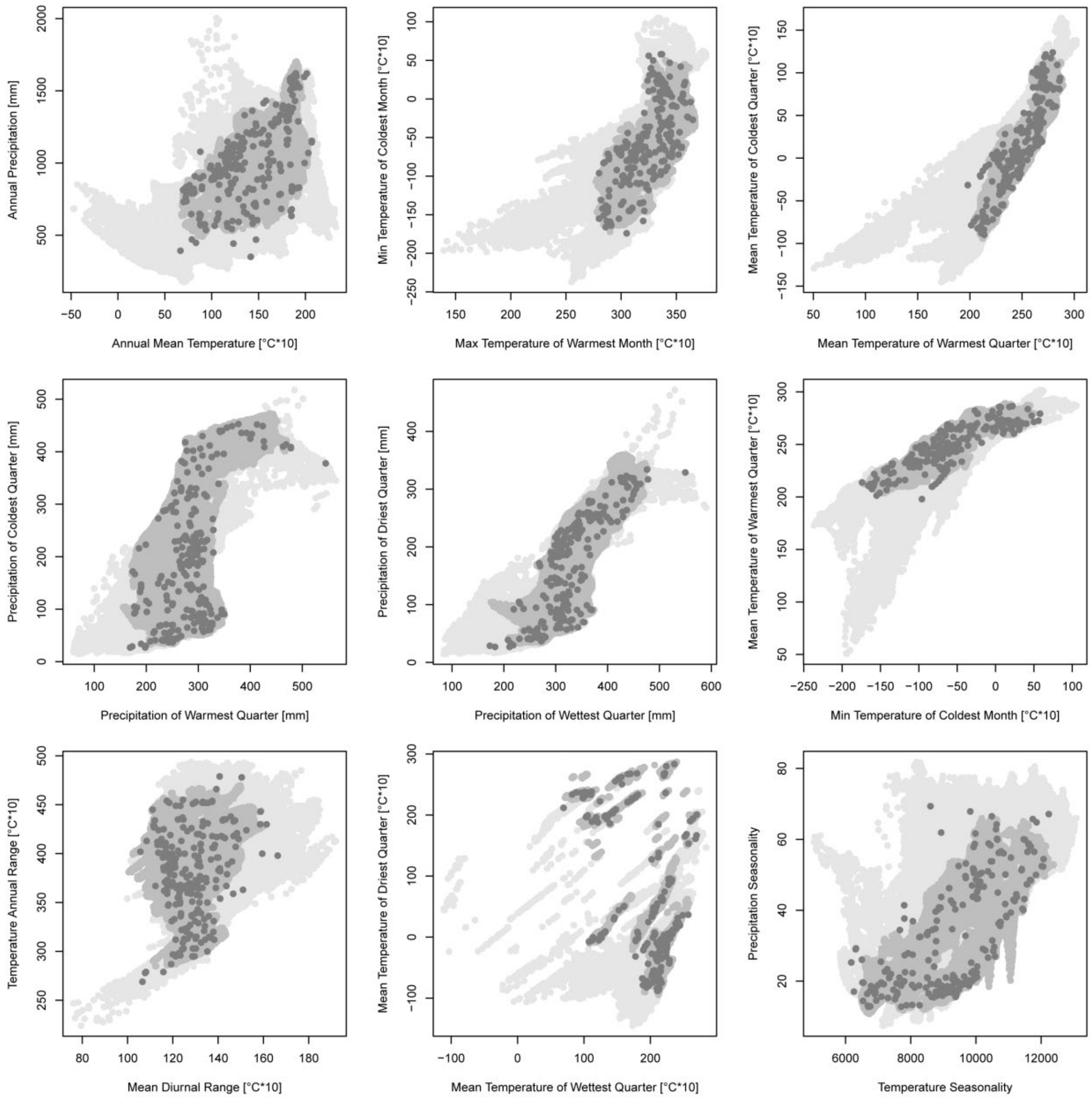

number of samples: 253

number of fossils: 0

sp4 − Apalone spinifera

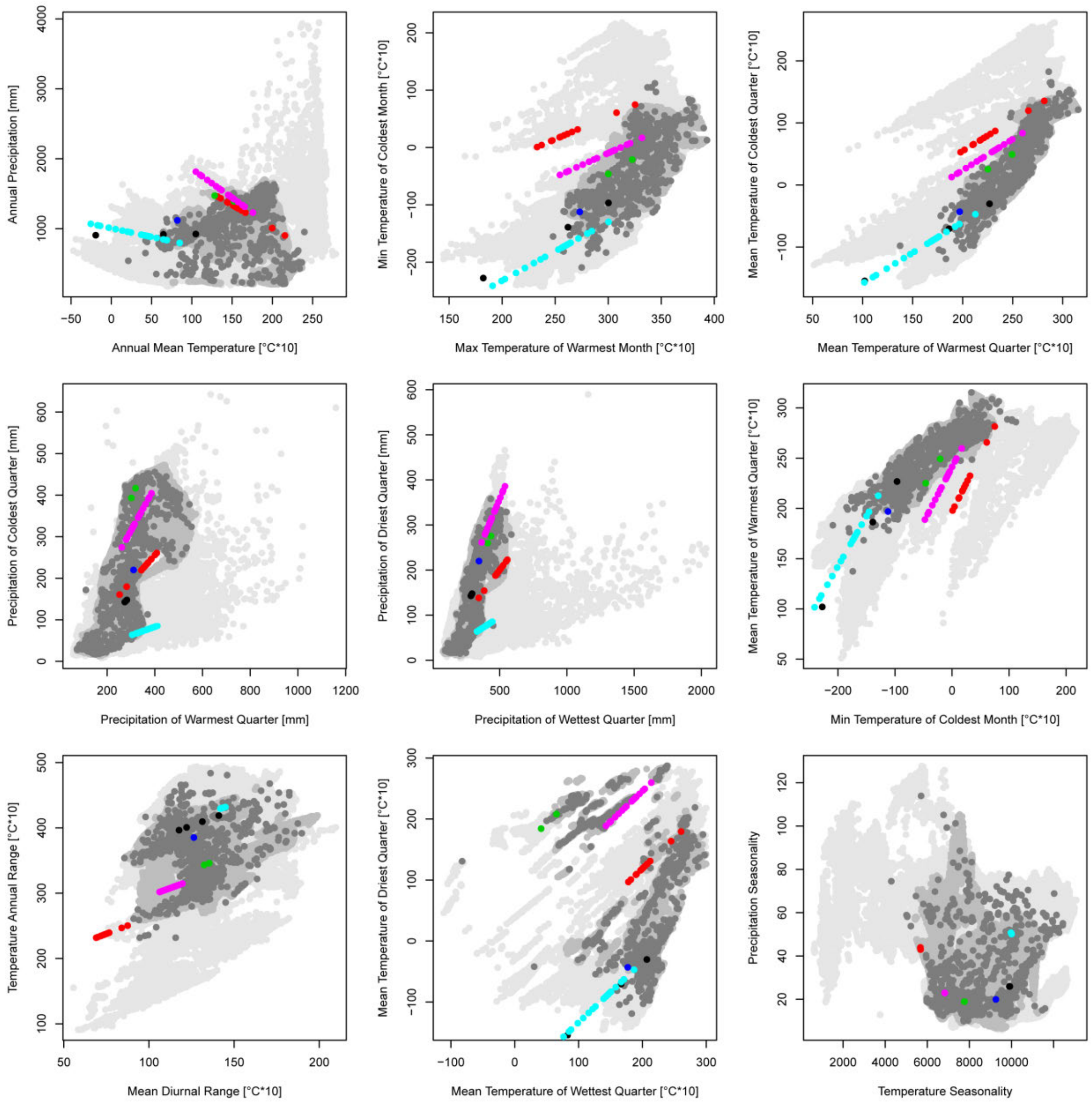

number of samples: 1034

number of fossils: 6

sp5 − Chelydra serpentina

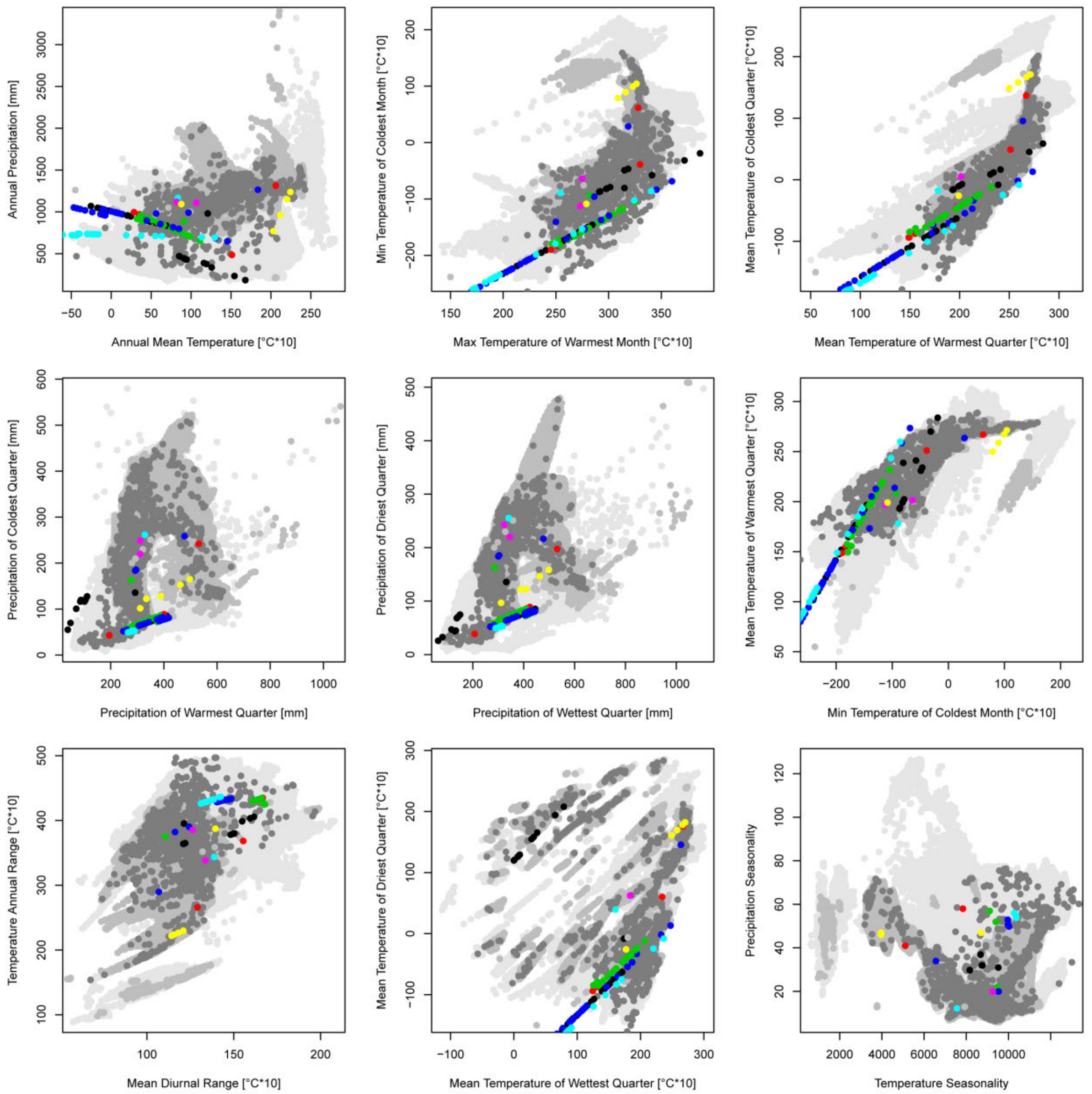

number of samples: 1295

number of fossils: 21

sp6 − Chrysemys dorsalis

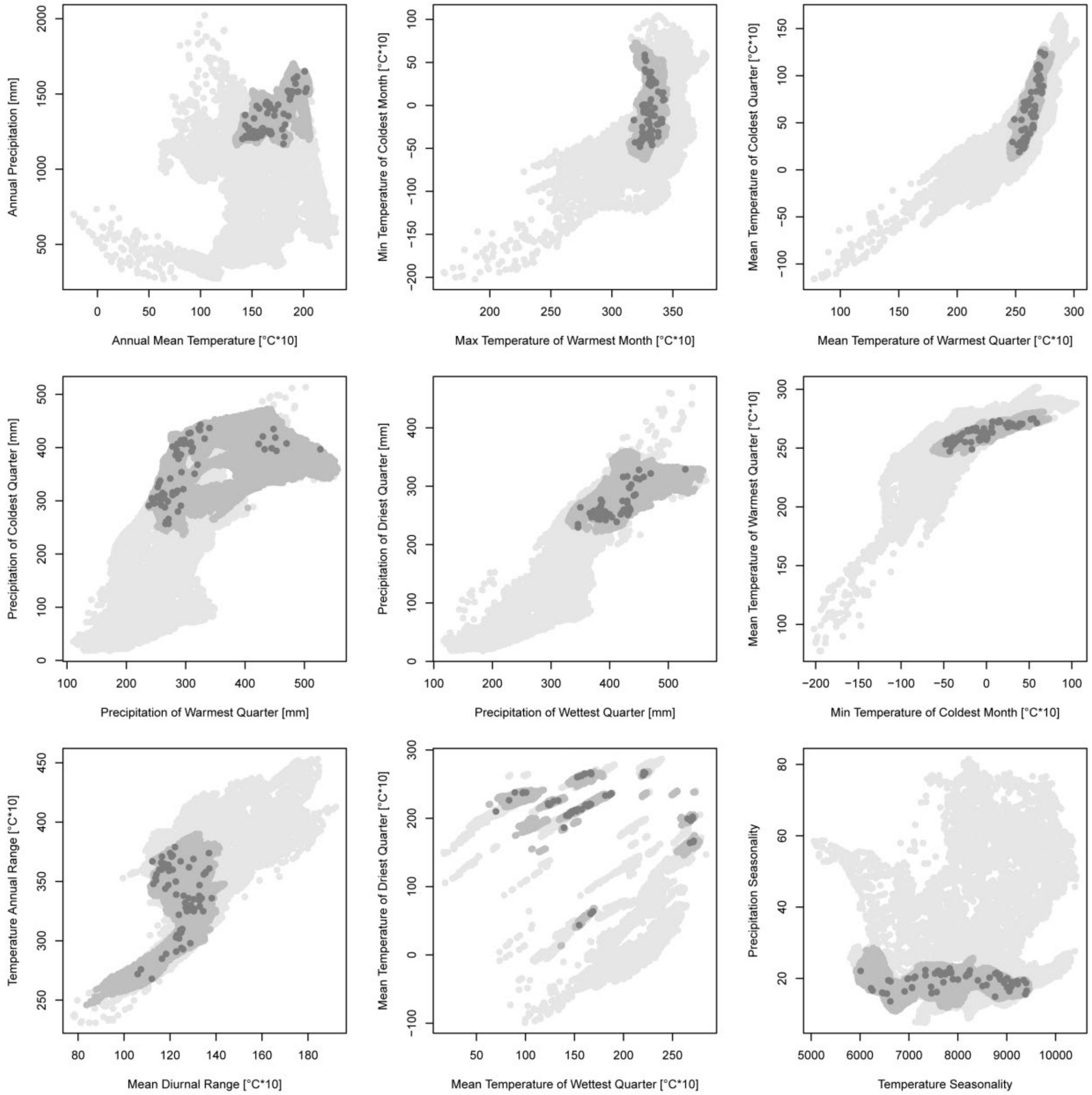

number of samples: 76

number of fossils: 0

sp7 − Chrysemys picta

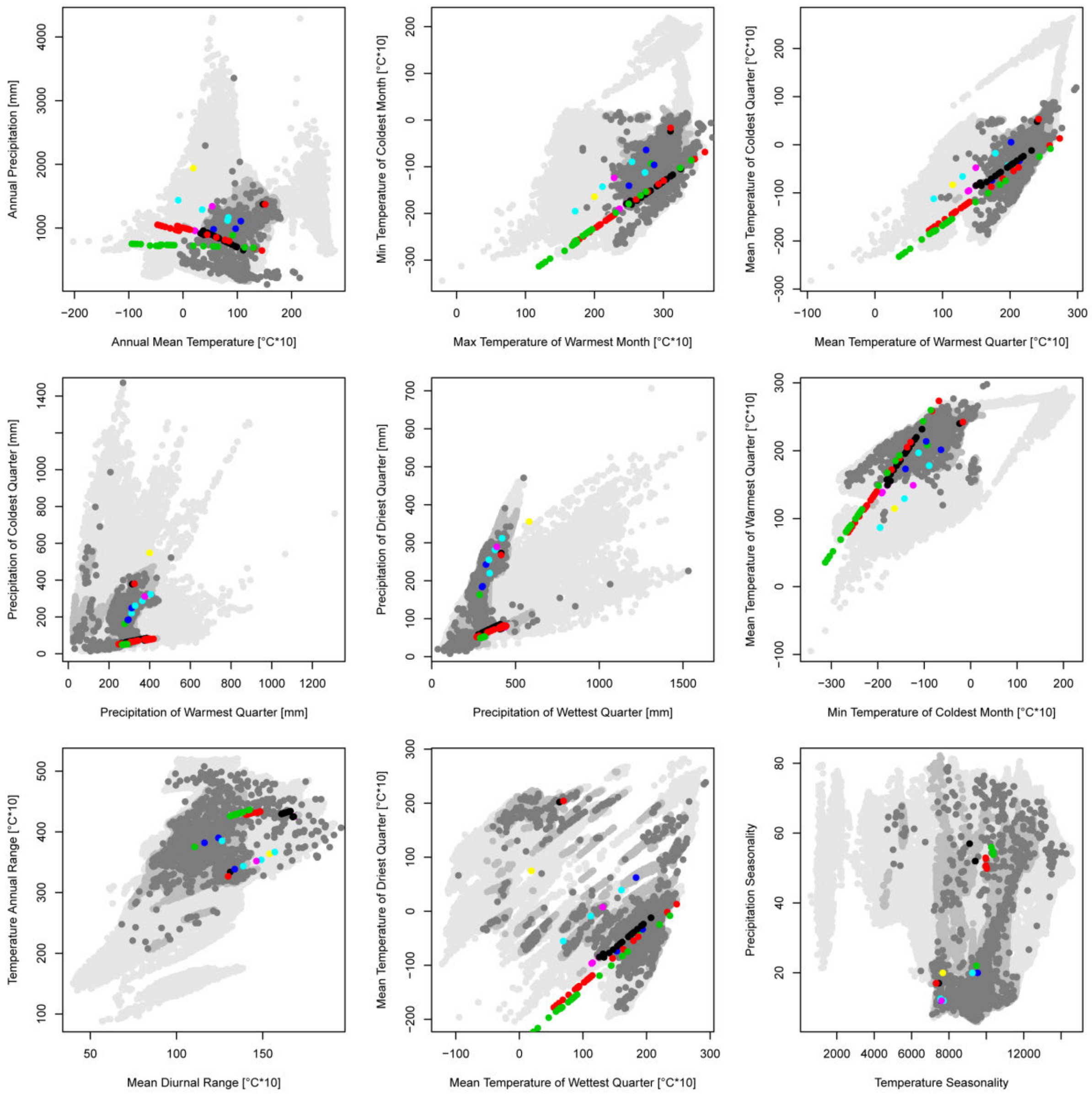

number of samples: 1823

number of fossils: 14

sp8 − Clemmys guttata

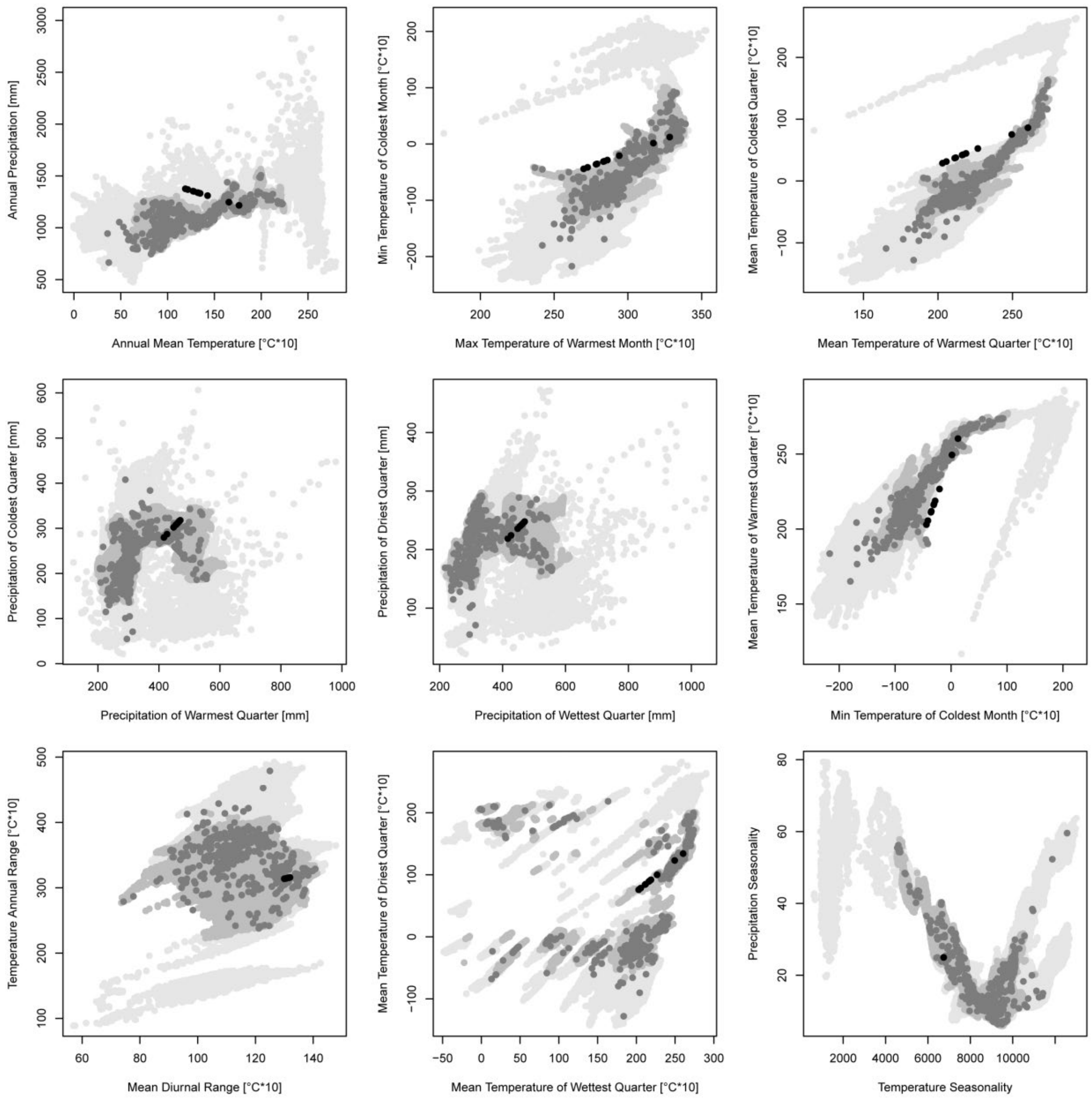

number of samples: 496

number of fossils: 1

sp9 − Deirochelys reticularia

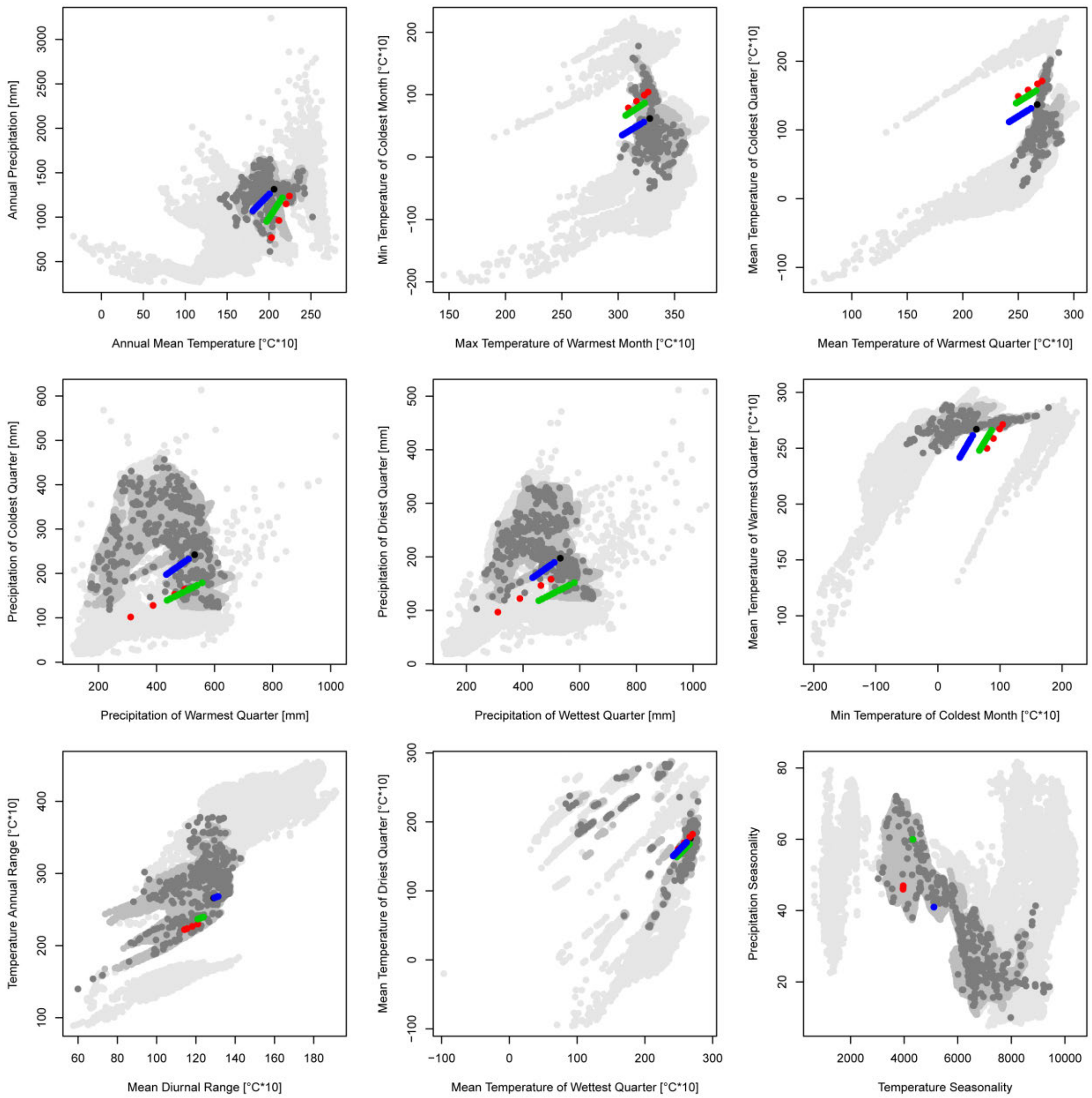

number of samples: 447

number of fossils: 4

sp10 − Emydoidea blandingii

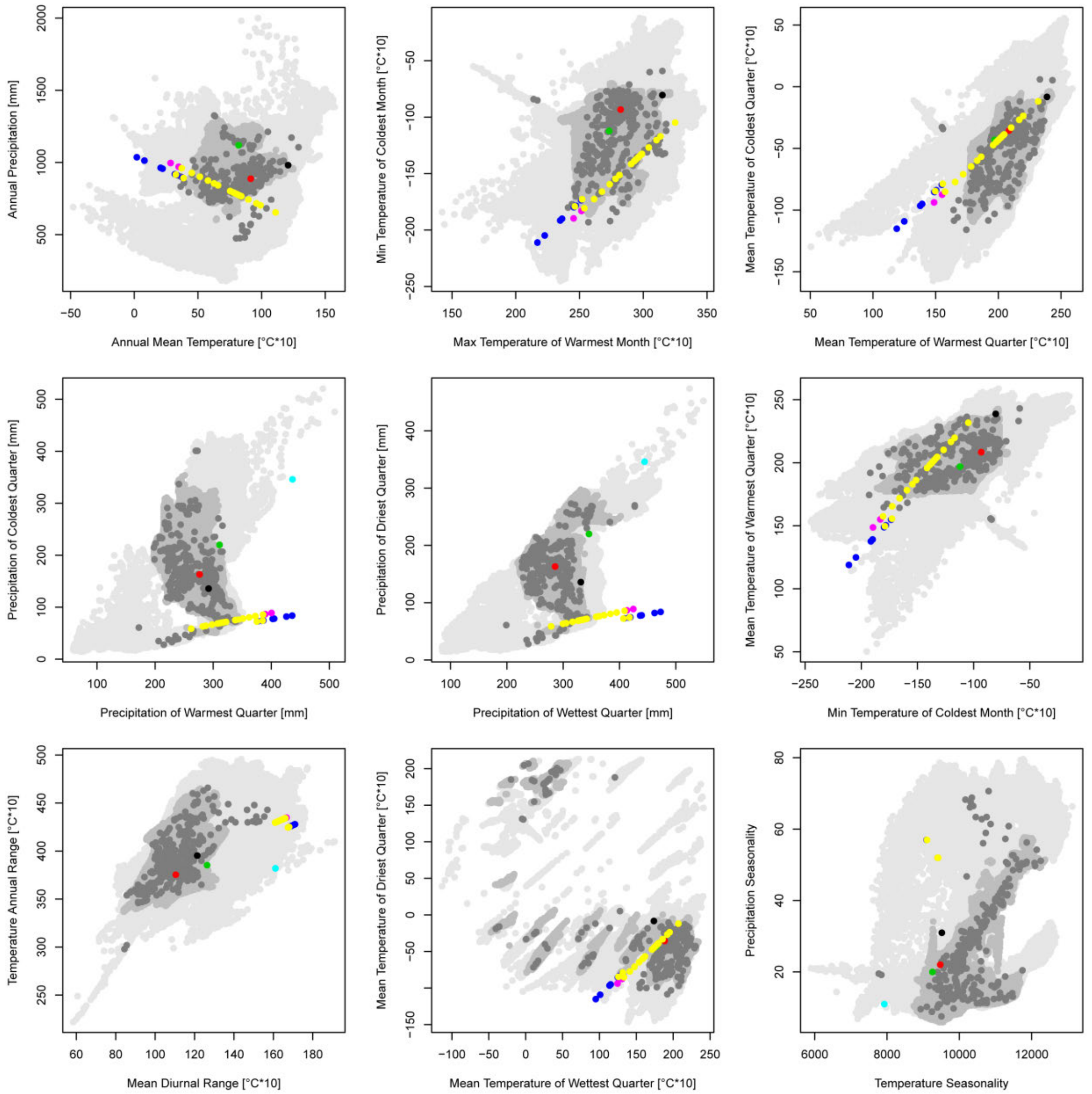

number of samples: 391

number of fossils: 7

sp11 − Glyptemys insculpta

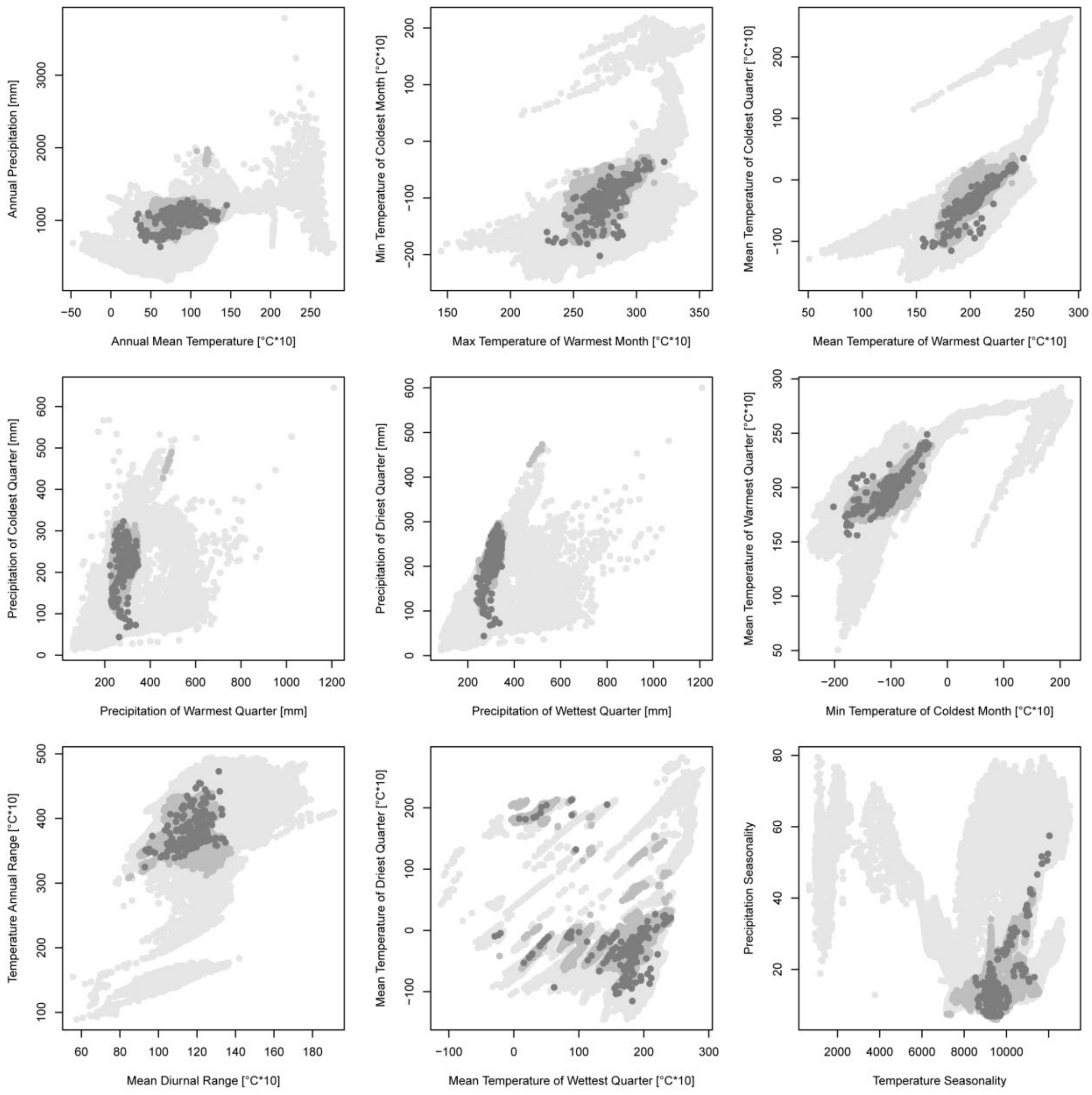

number of samples: 293

number of fossils: 0

sp12 − Glyptemys muhlenbergii

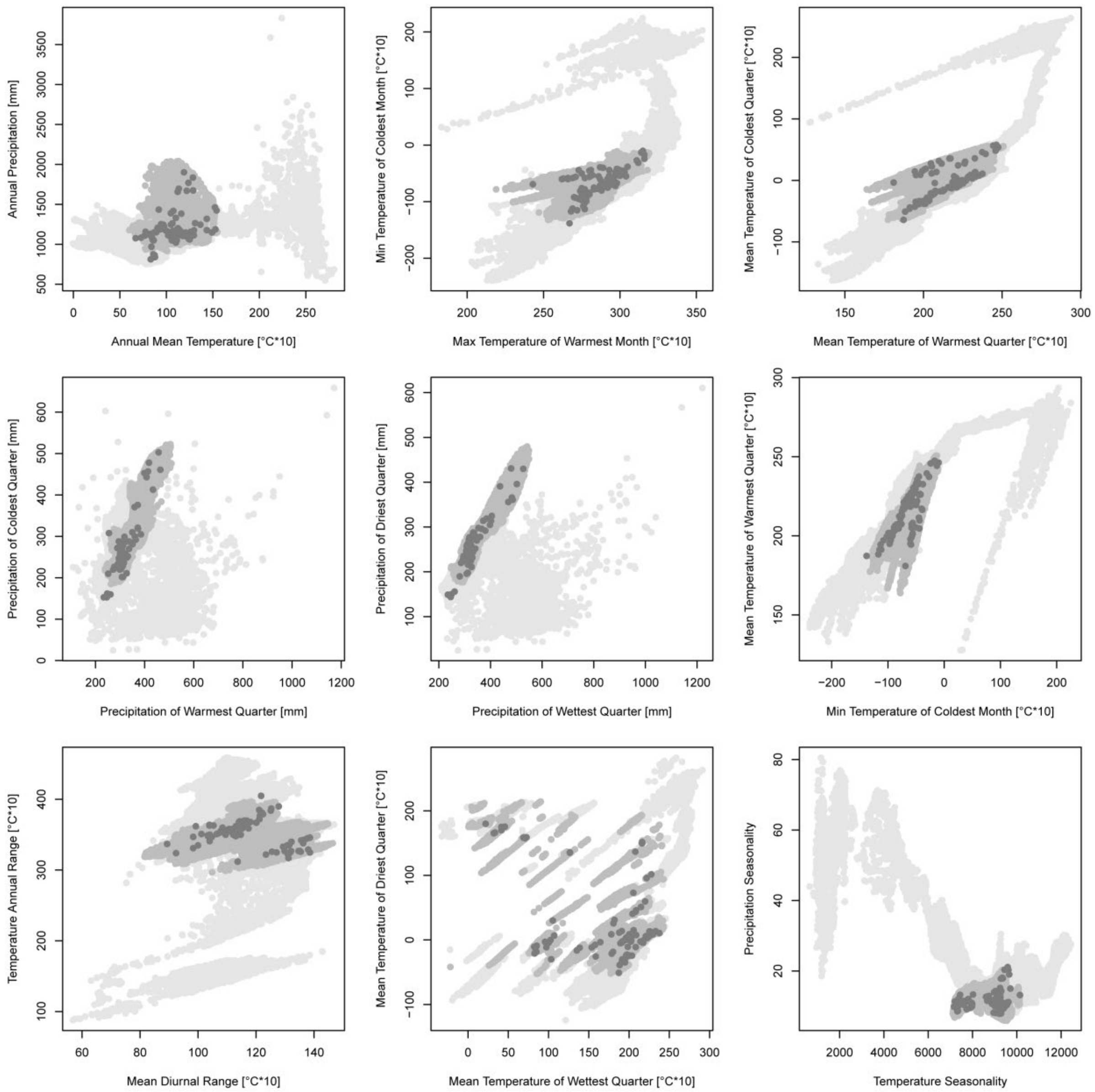

number of samples: 104

number of fossils: 0

sp13 − *Graptemys barbouri*

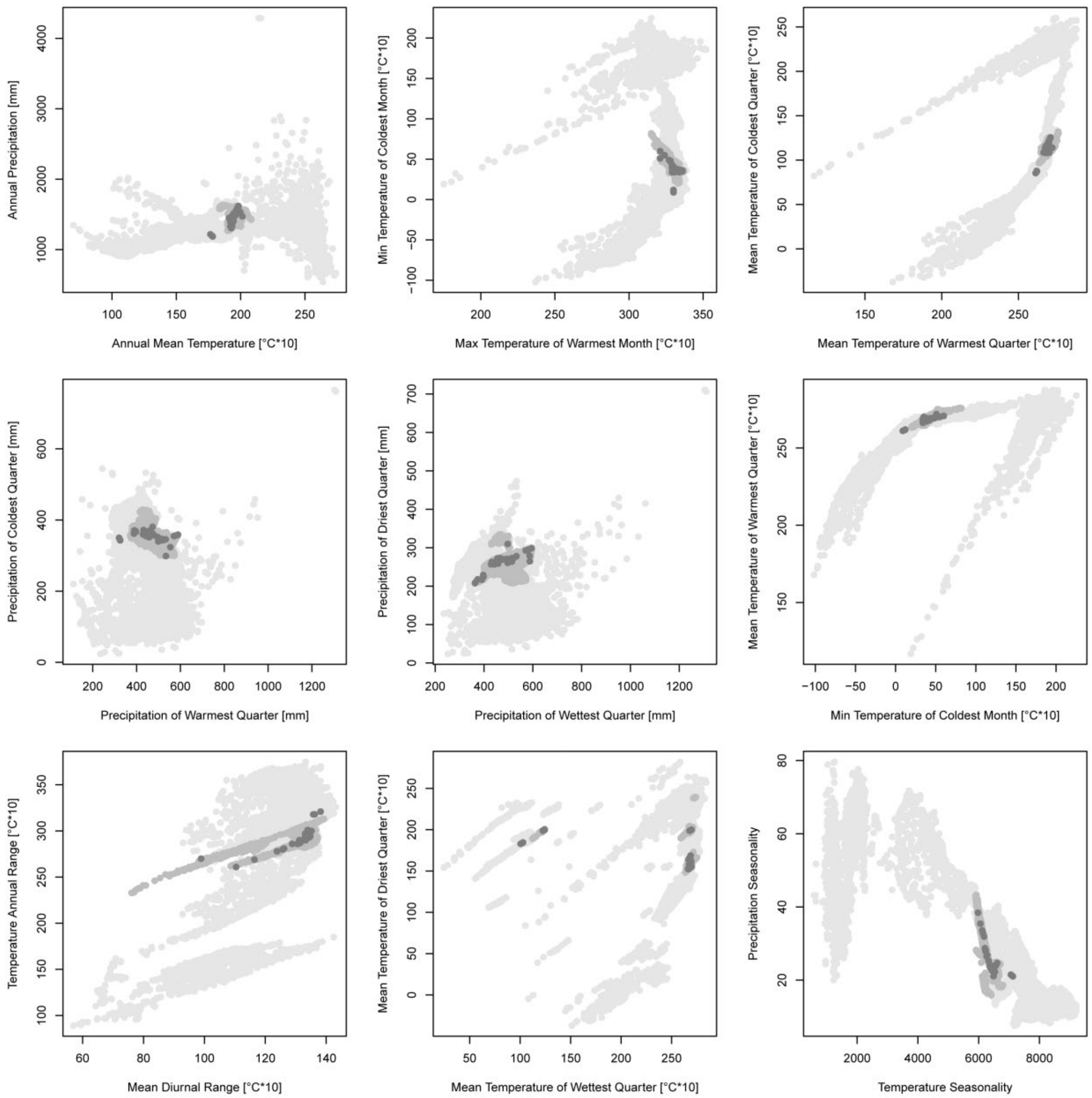

number of samples: 45

number of fossils: 0

sp14 − Graptemys caglei

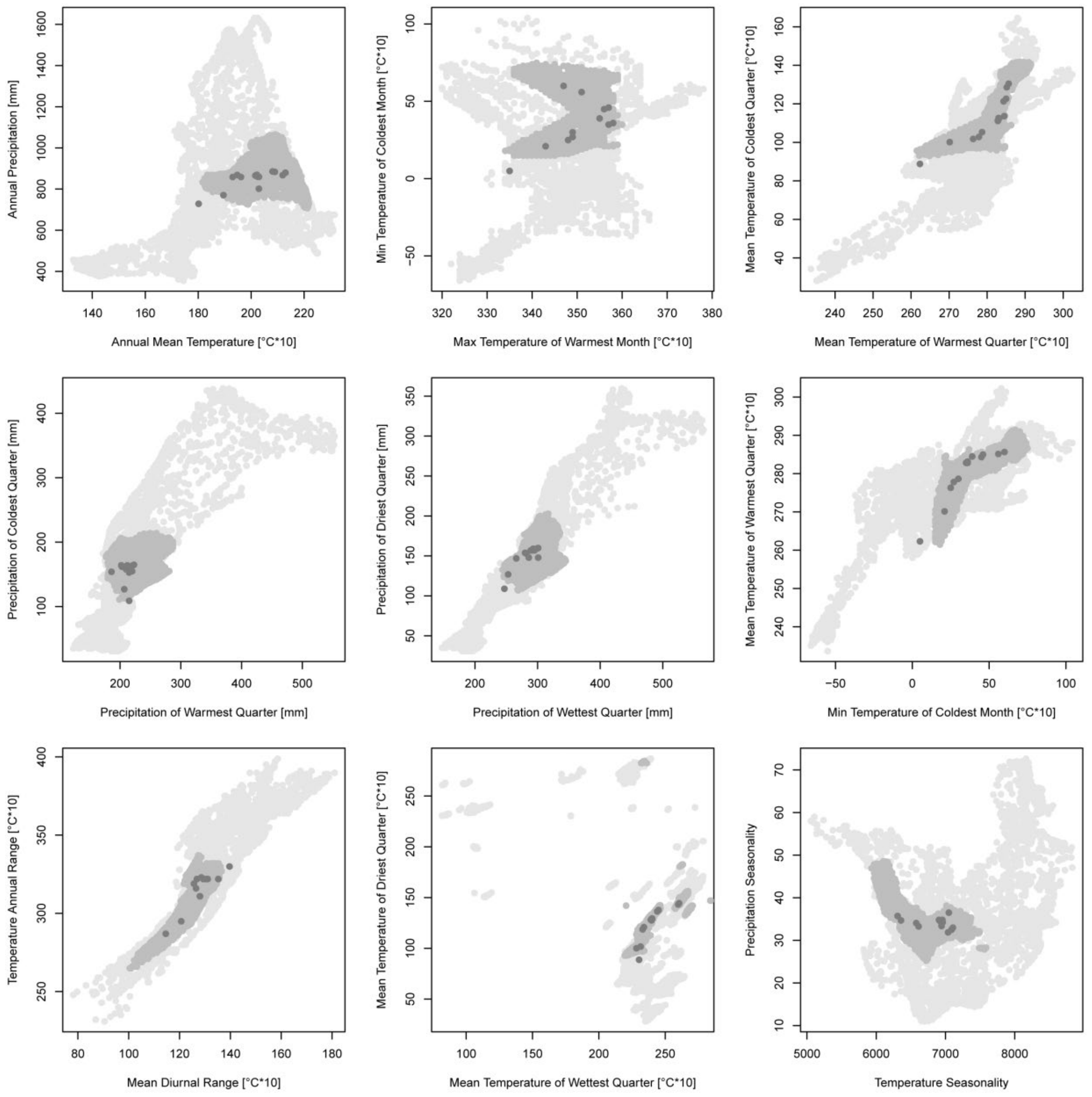

number of samples: 14

number of fossils: 0

sp15 − Graptemys ernsti

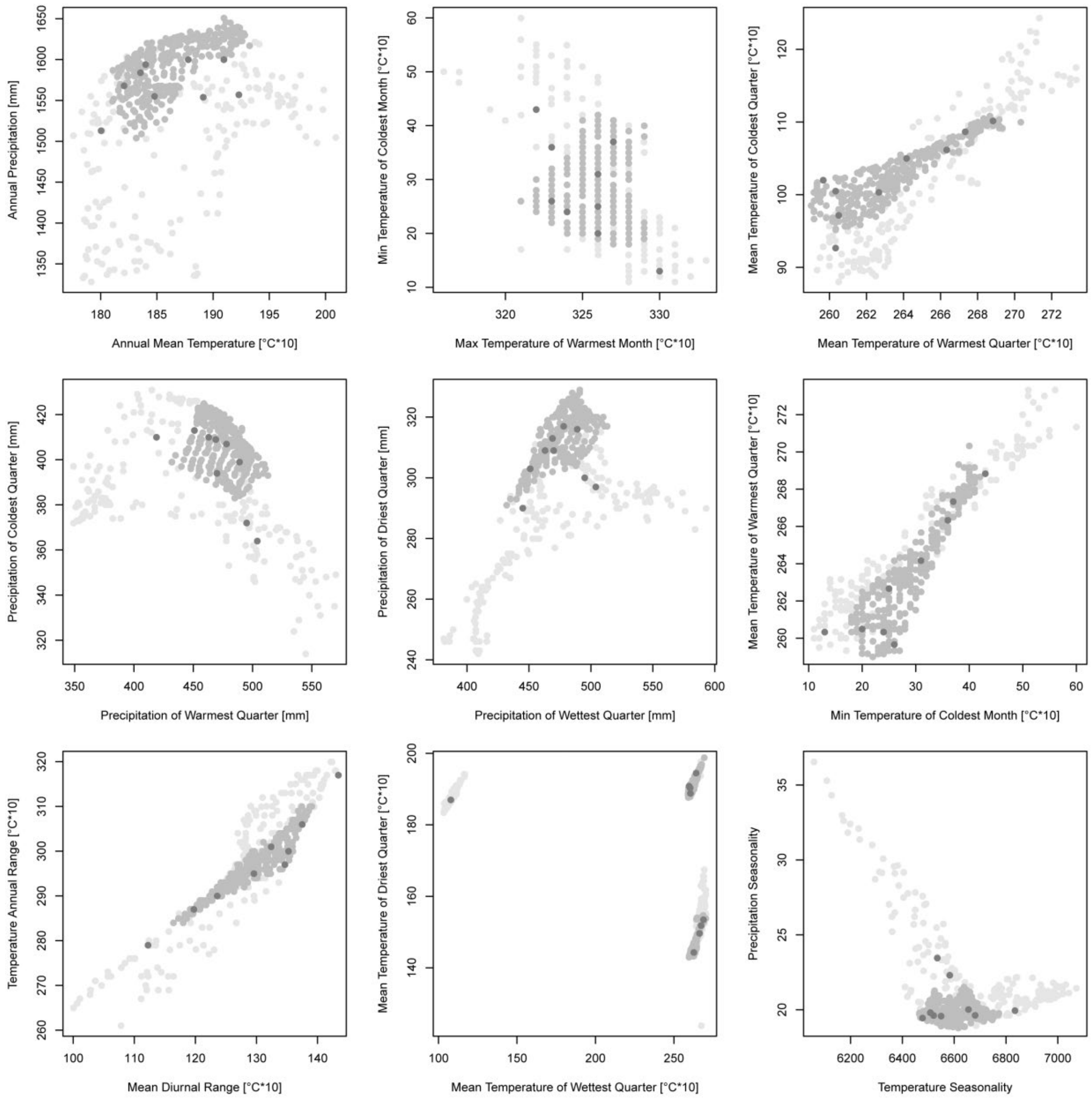

number of samples: 11

number of fossils: 0

sp16 − Graptemys flavimaculata

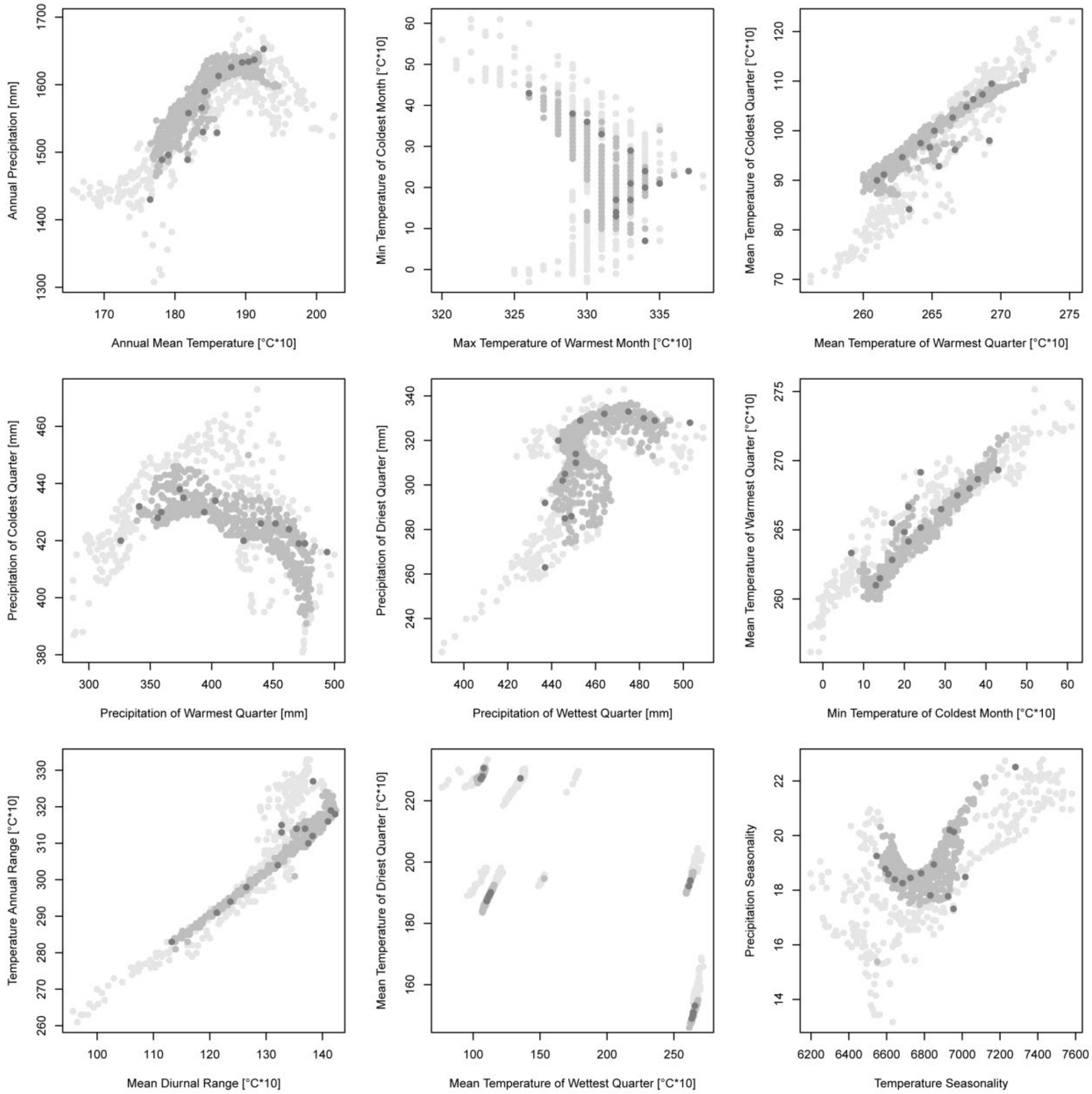

number of samples: 16

number of fossils: 0

sp17 − Graptemys geographica

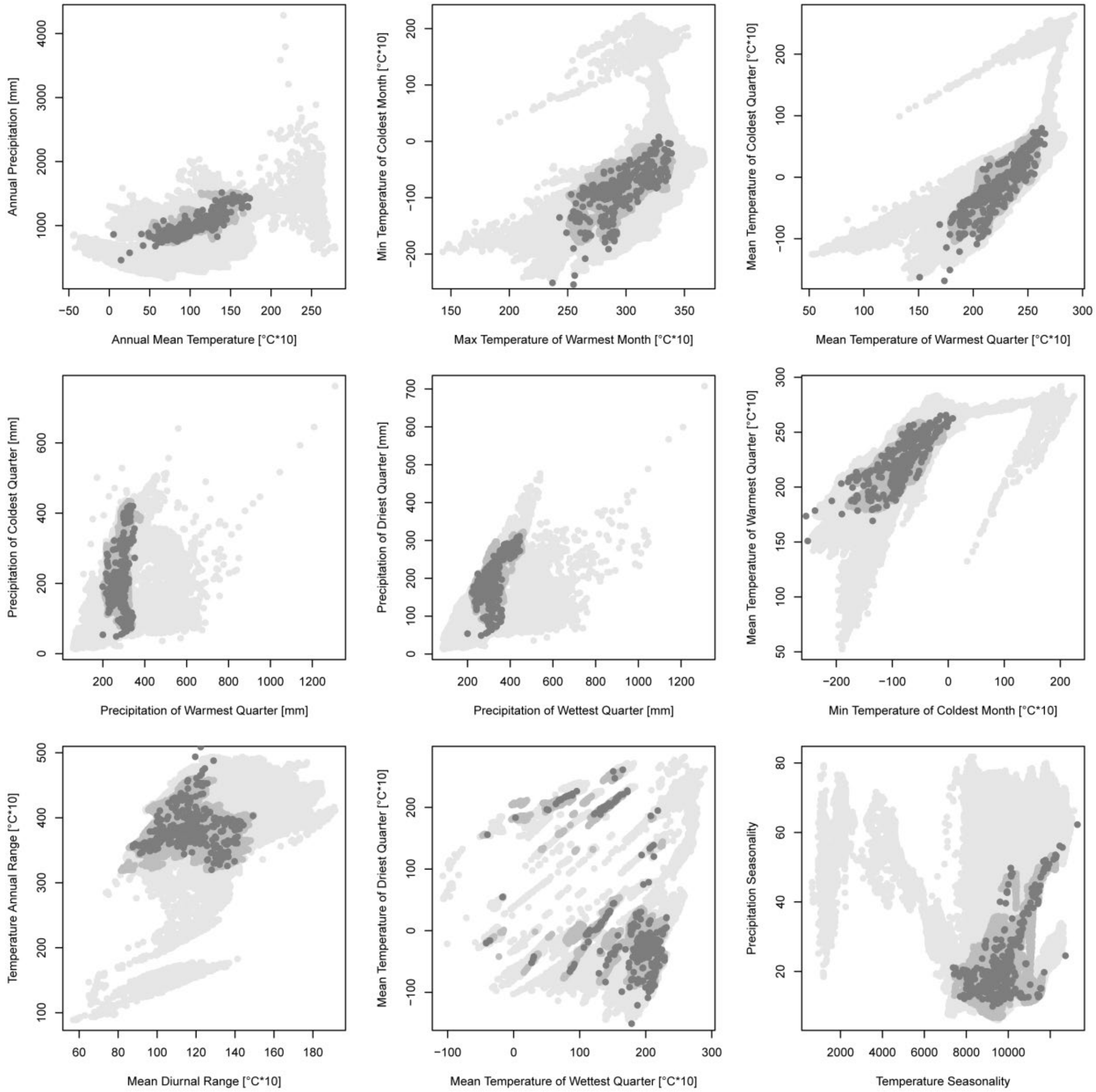

number of samples: 436

number of fossils: 0

sp18 − Graptemys gibbonsi

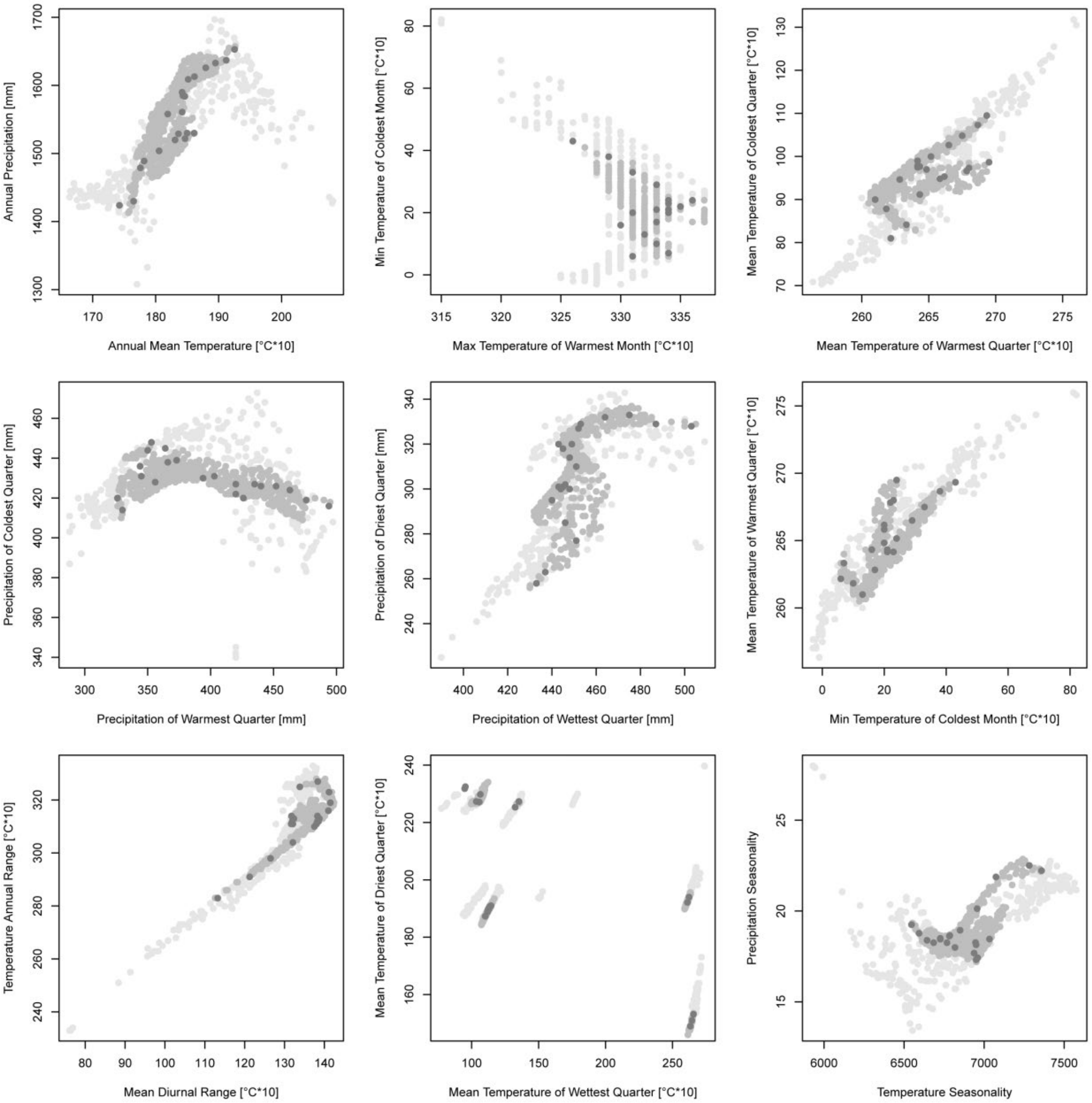

number of samples: 21

number of fossils: 0

sp19 − Graptemys nigrinoda

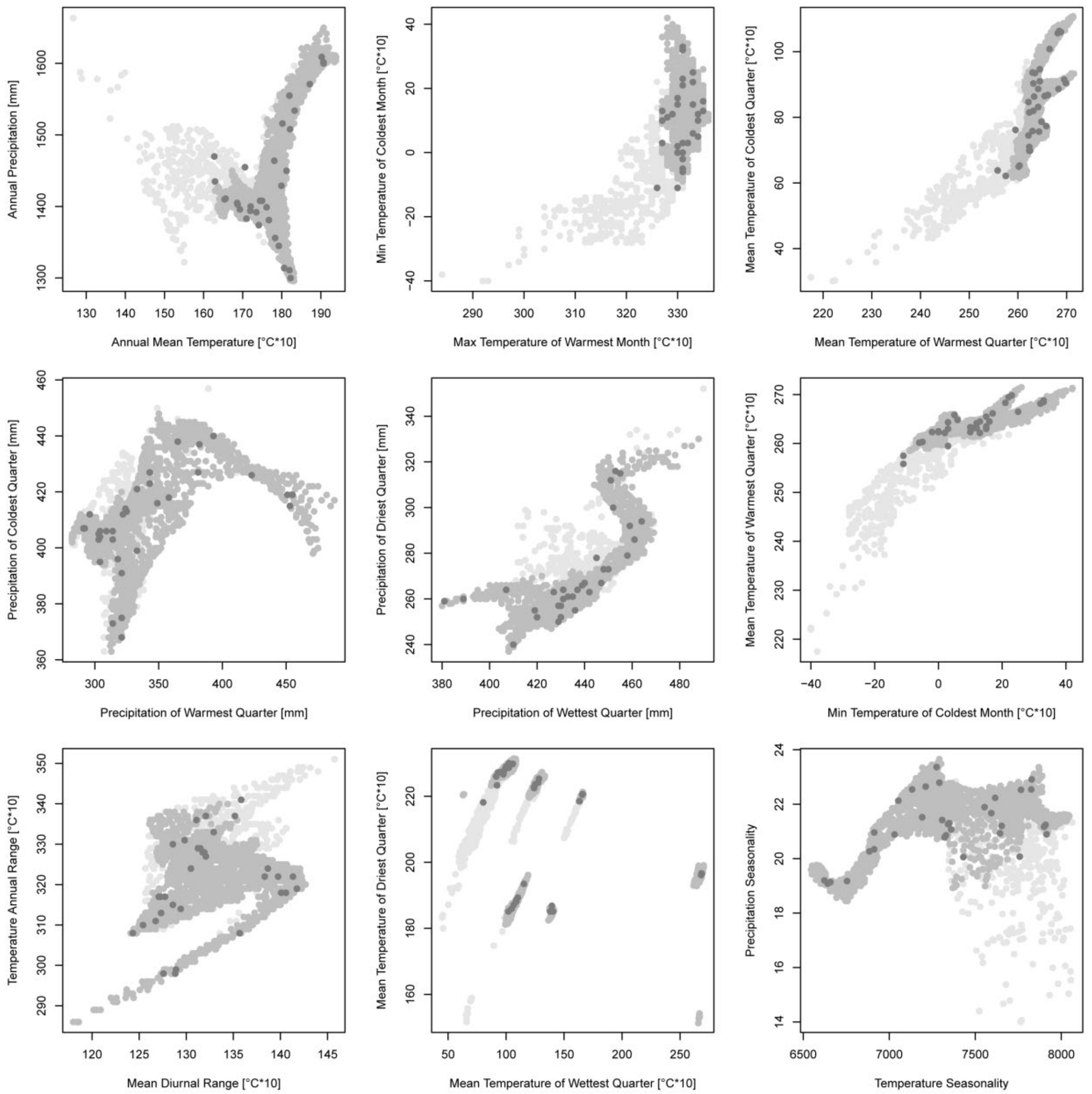

number of samples: 33

number of fossils: 0

sp20 − Graptemys oculifera

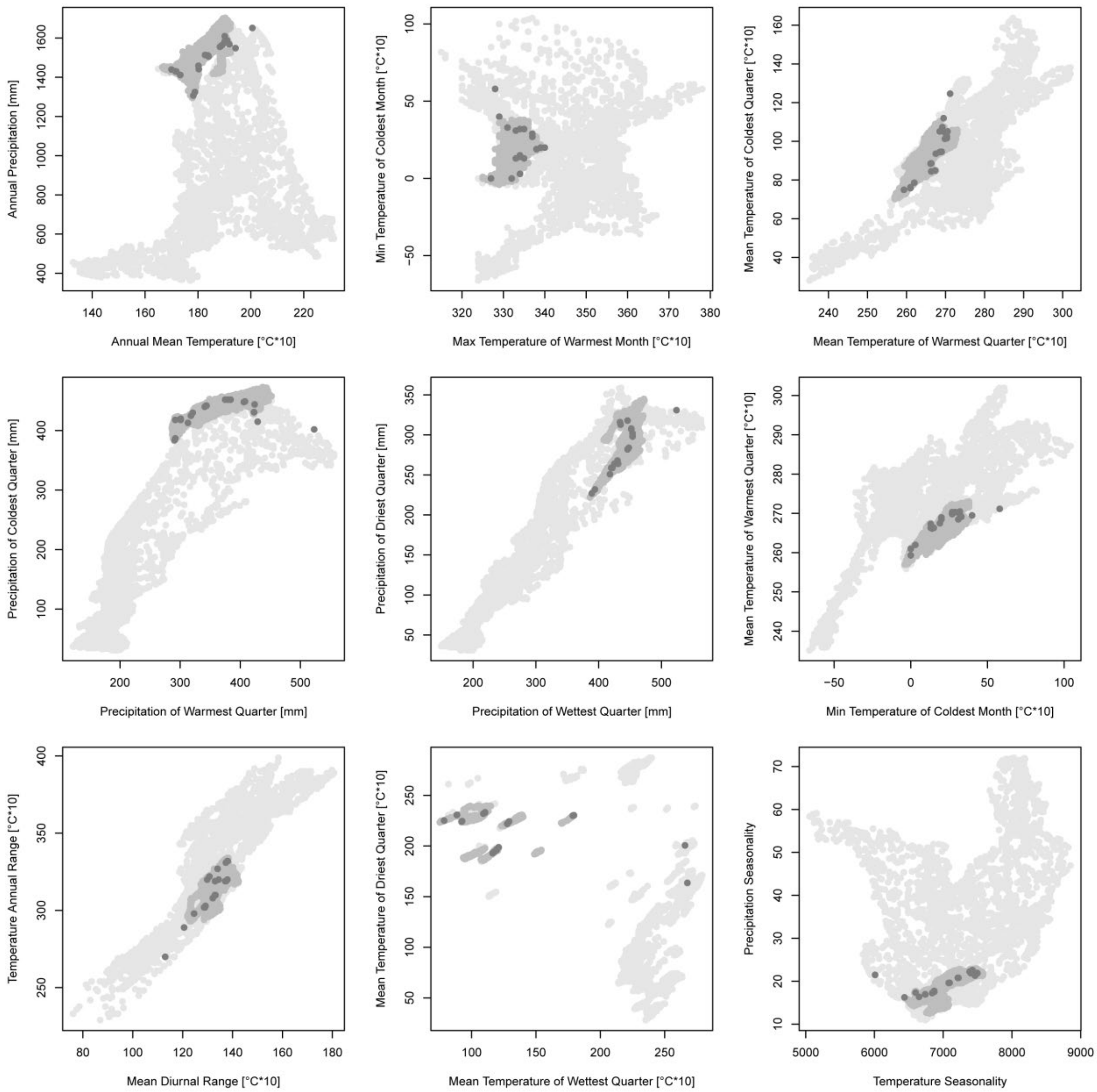

number of samples: 21

number of fossils: 0

sp21 − Graptemys ouachitensis

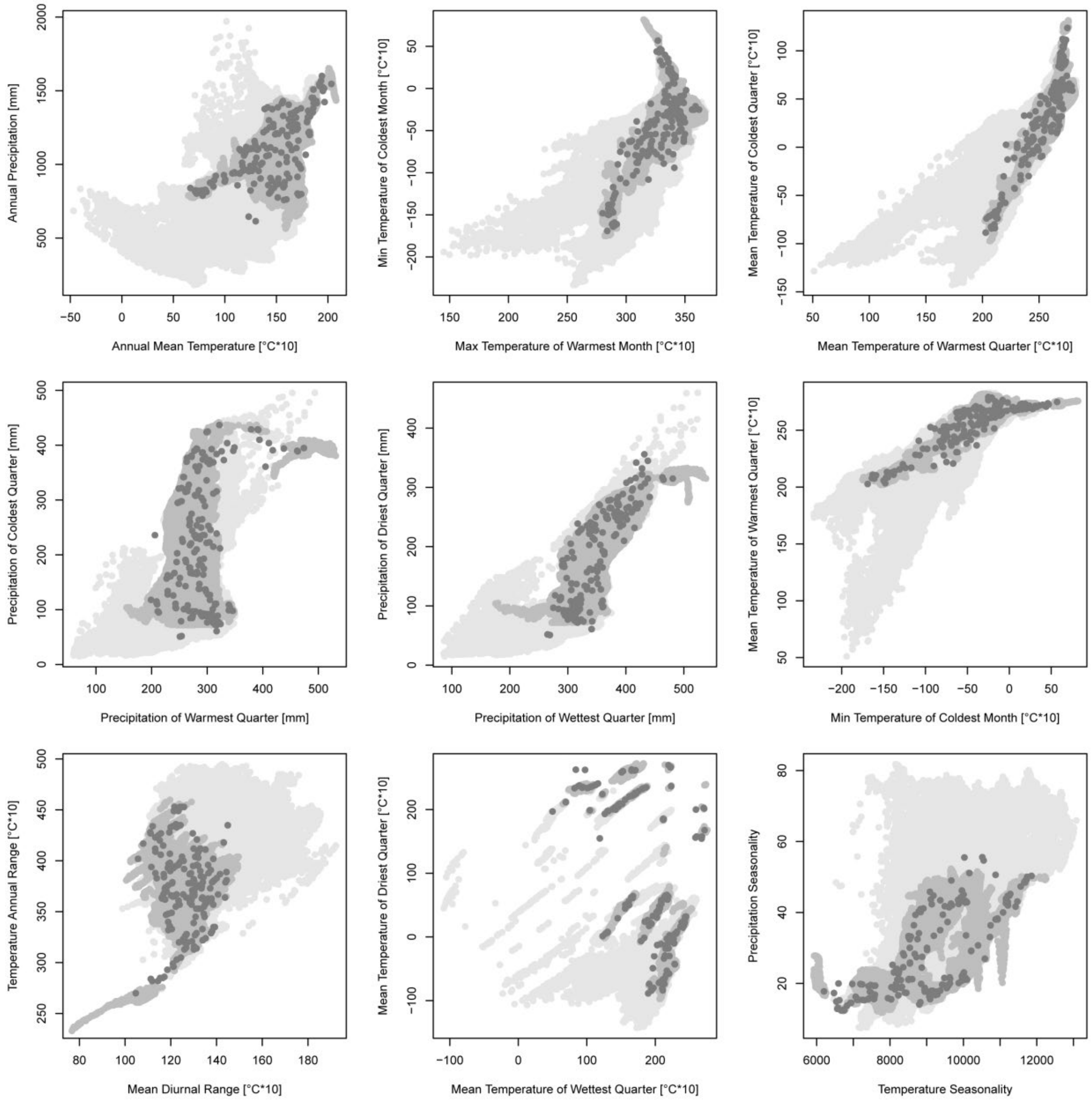

number of samples: 191

number of fossils: 0

sp22 − Graptemys pearlensis

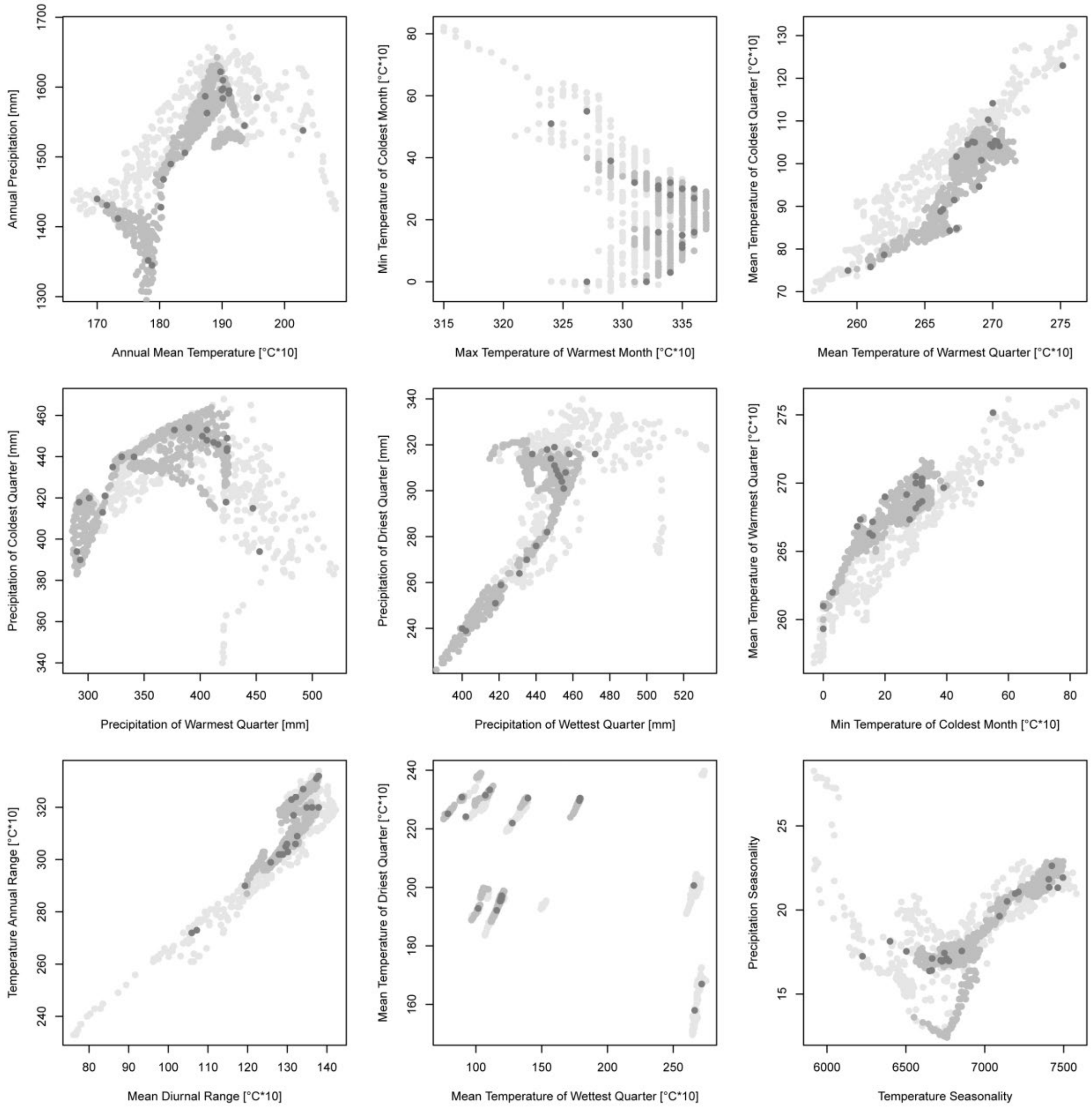

number of samples: 23

number of fossils: 0

sp23 − Graptemys pseudogeographica

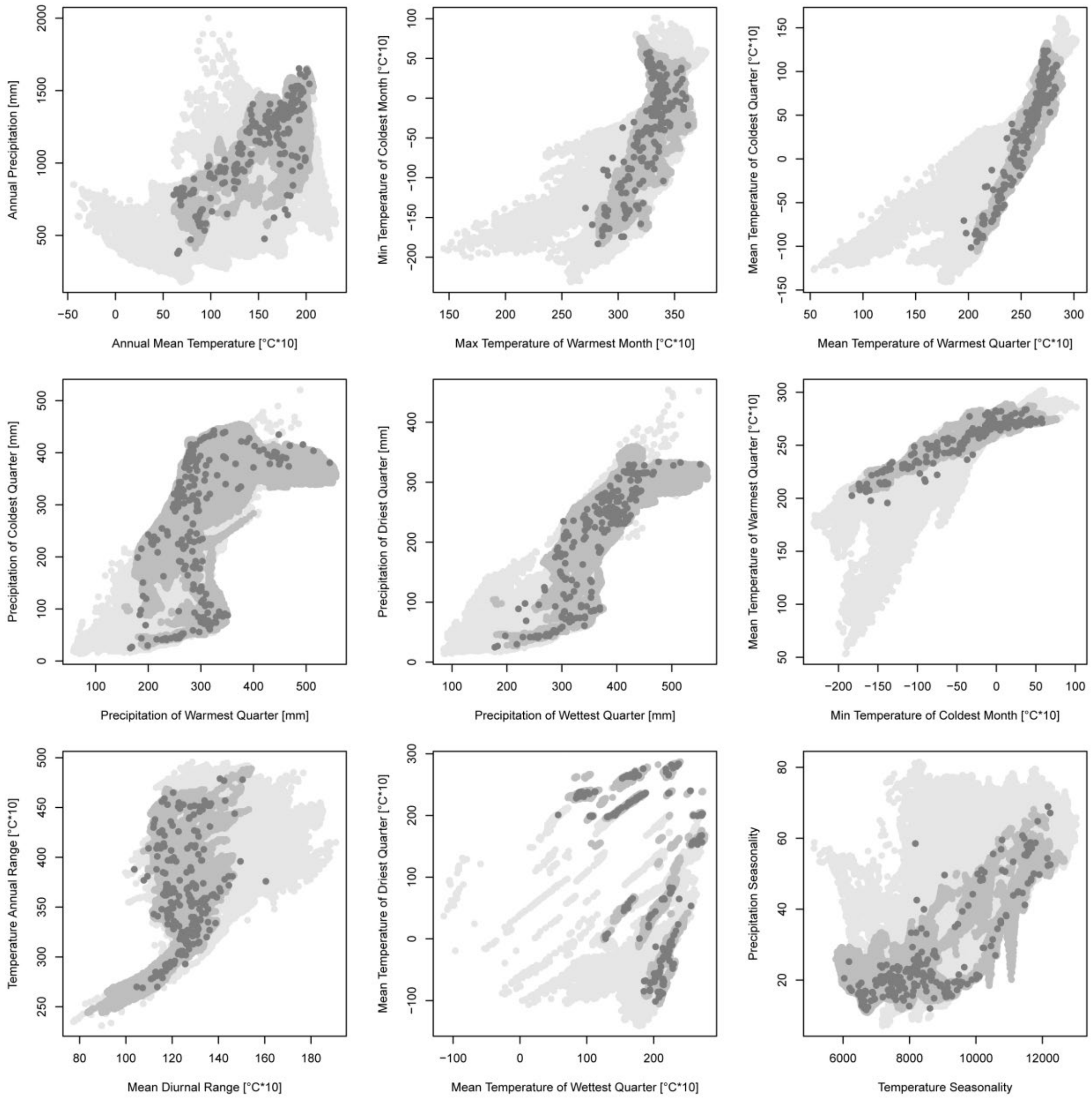

number of samples: 252

number of fossils: 0

sp24 − Graptemys pulchra

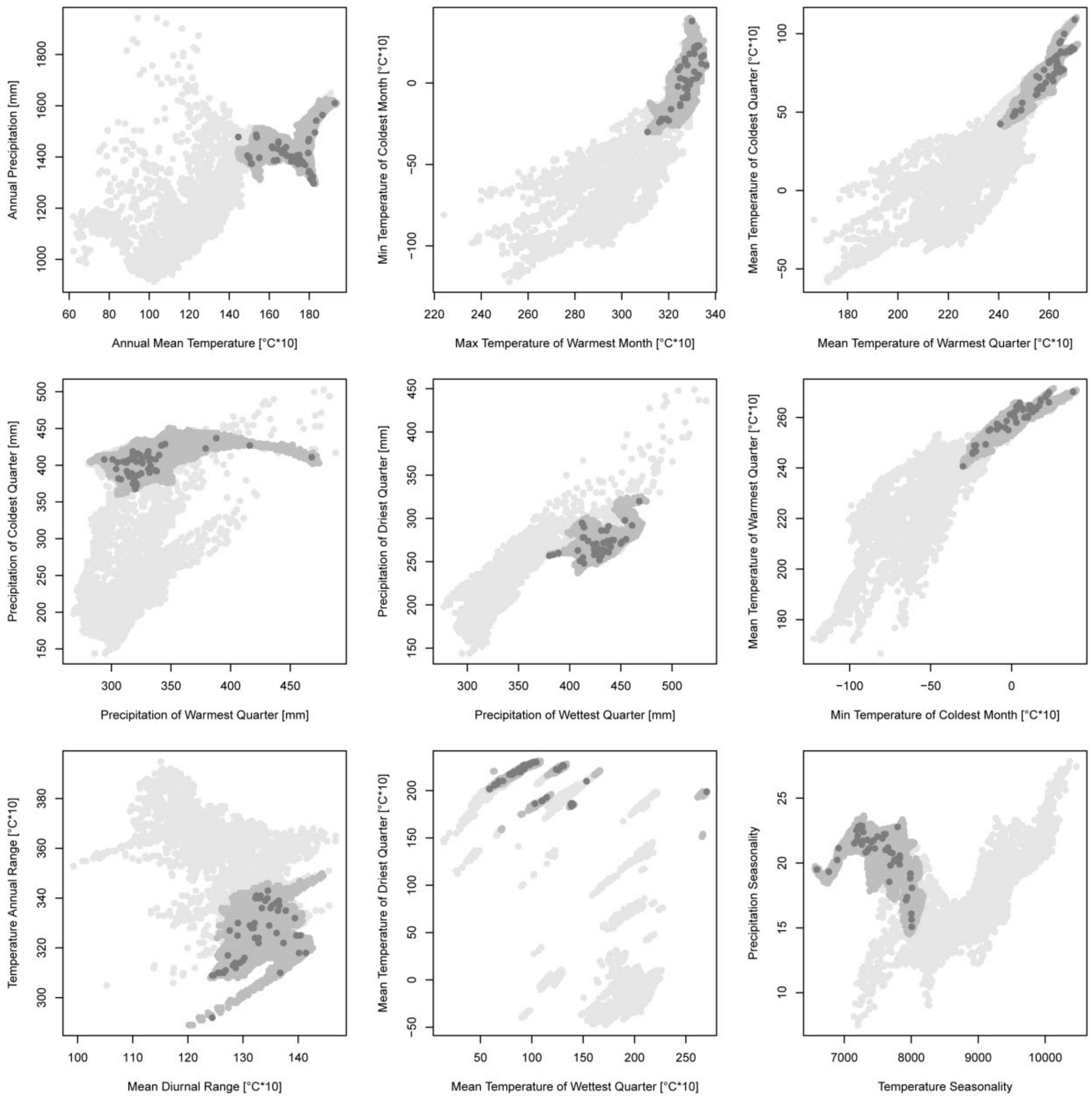

number of samples: 54

number of fossils: 0

sp25 − Graptemys versa

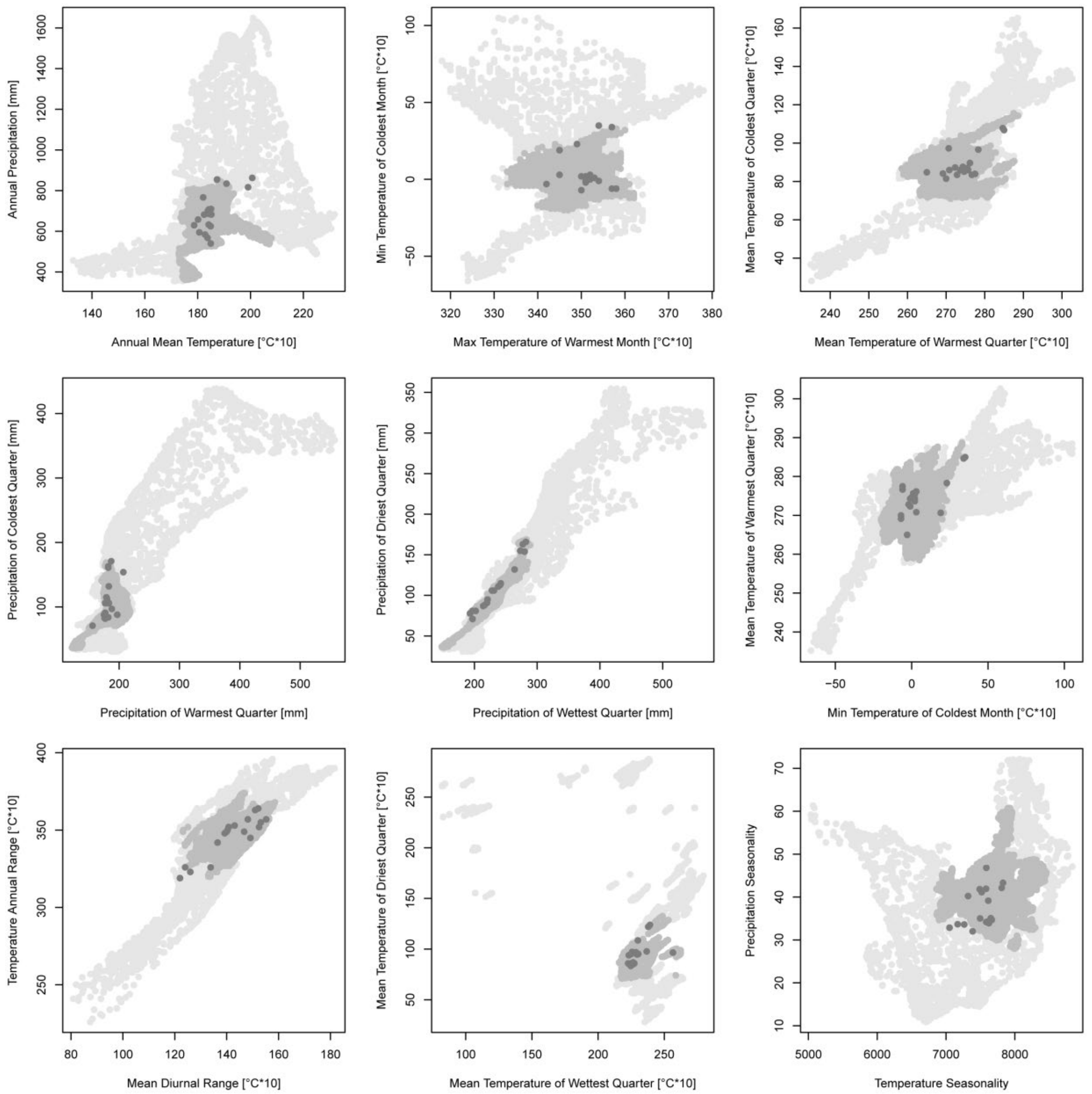

number of samples: 19

number of fossils: 0

sp26 − Kinosternon alamosae

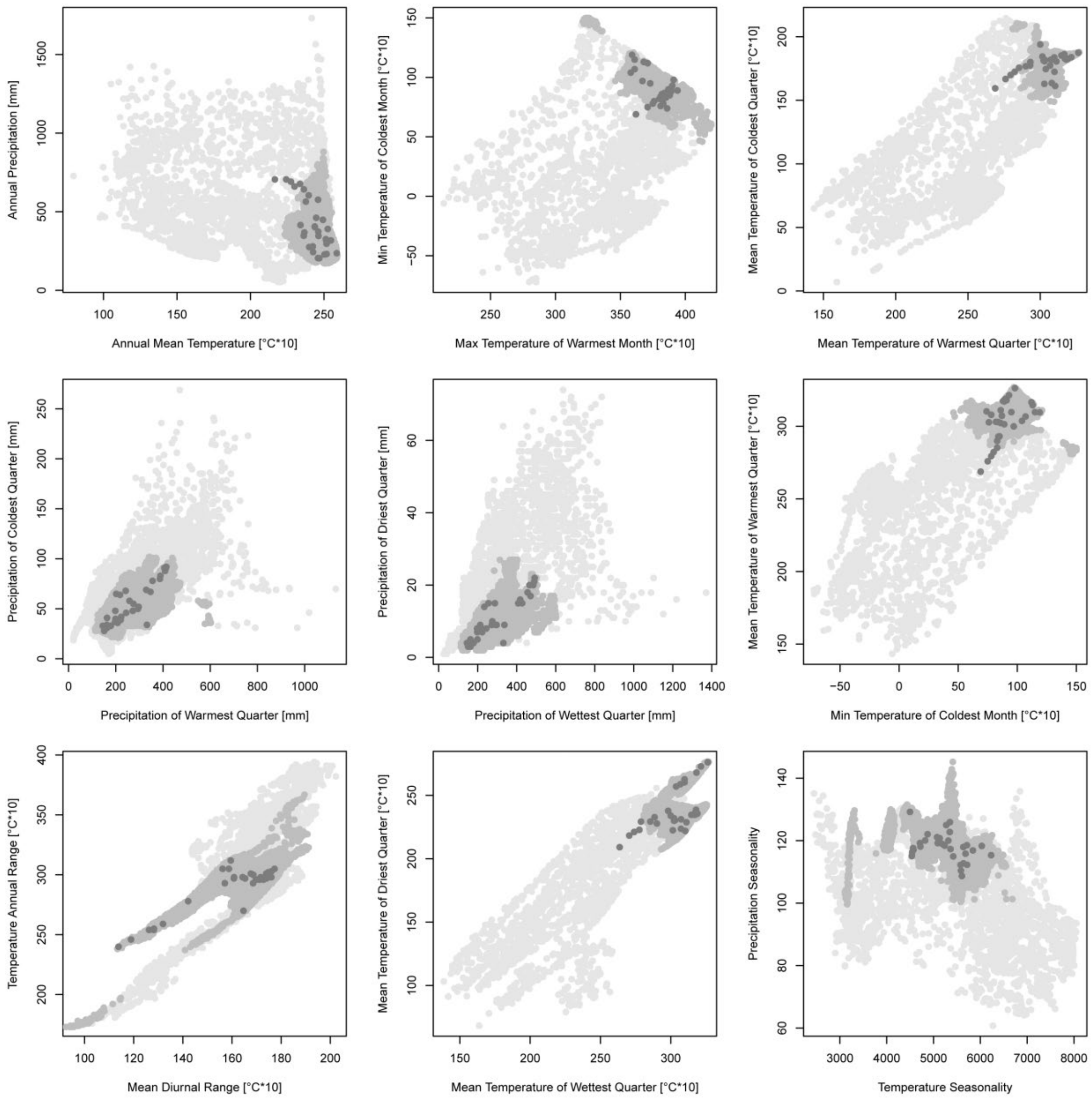

number of samples: 37

number of fossils: 0

sp27 − Kinosternon arizonense

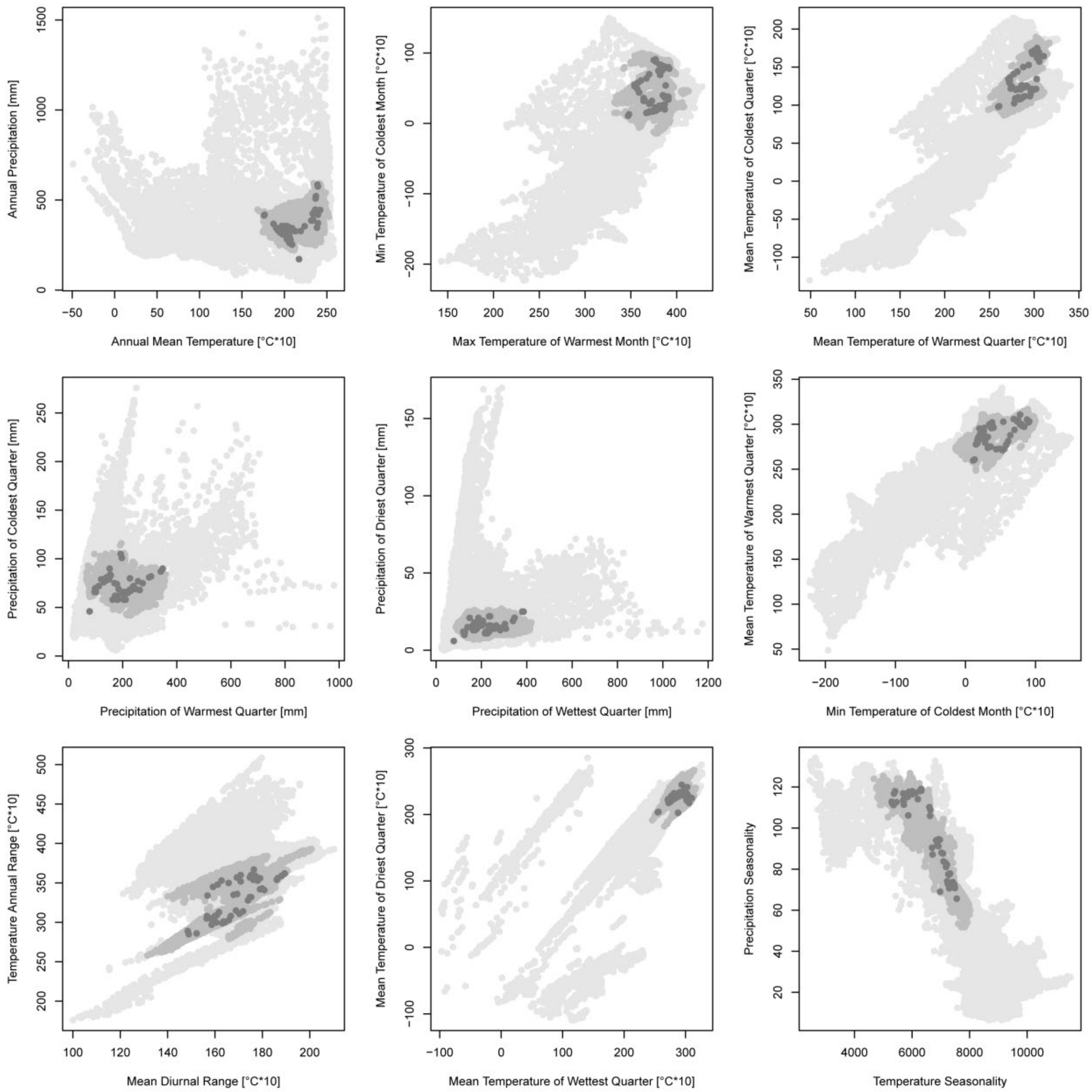

number of samples: 64

number of fossils: 0

sp28 − Kinosternon baurii

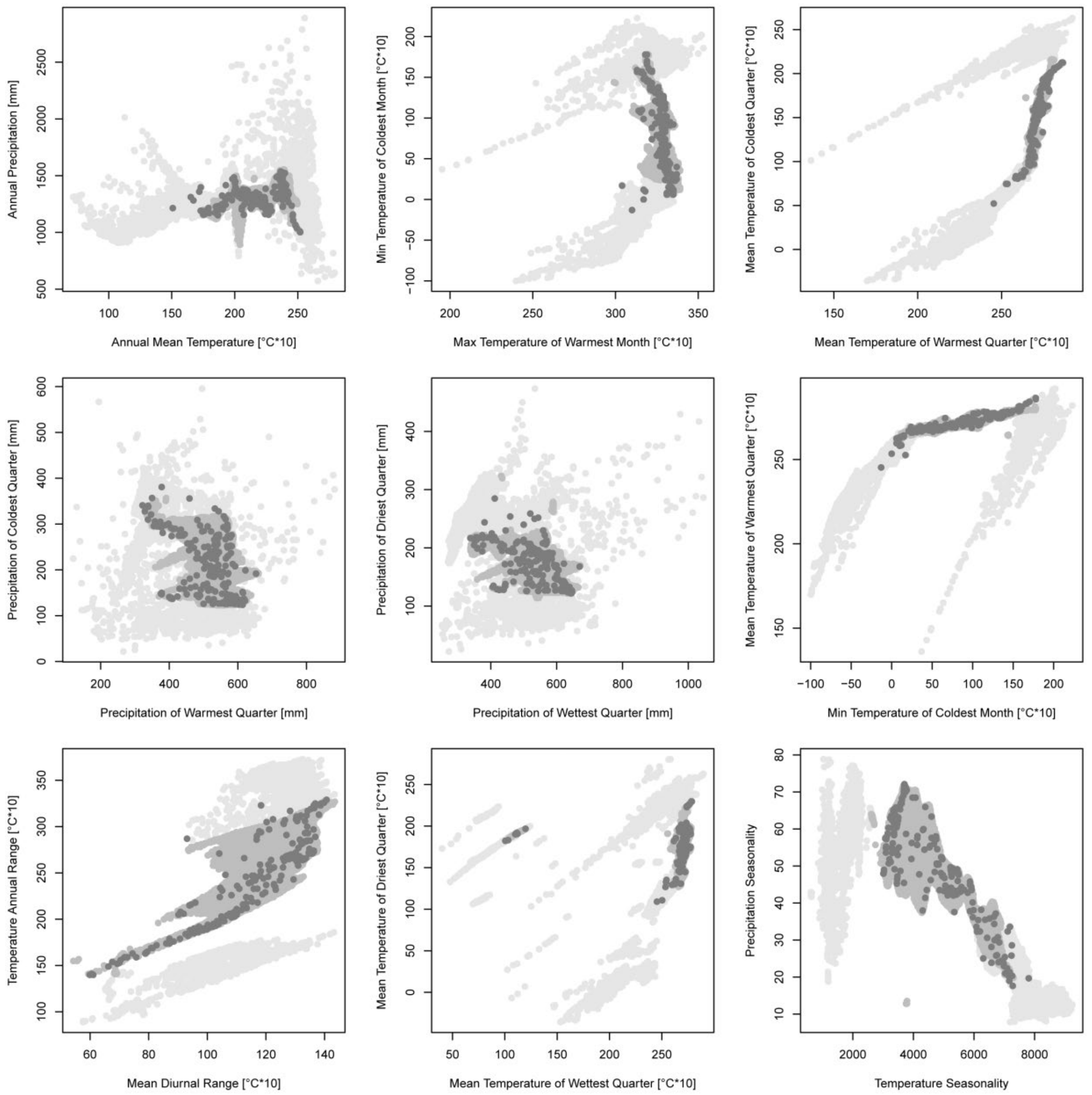

number of samples: 239

number of fossils: 0

sp29 − Kinosternon durangoense

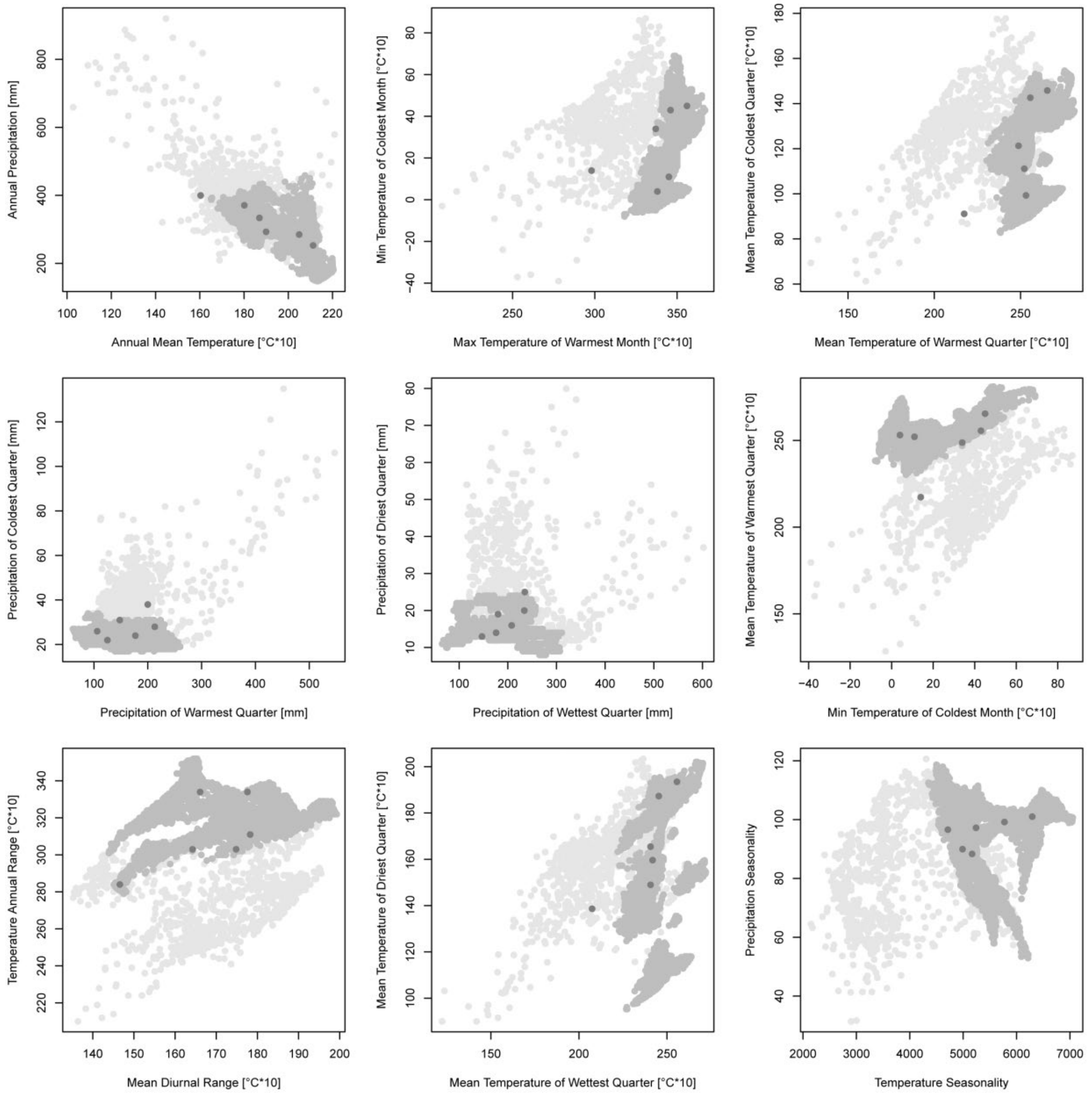

number of samples: 6

number of fossils: 0

sp30 − Kinosternon flavescens

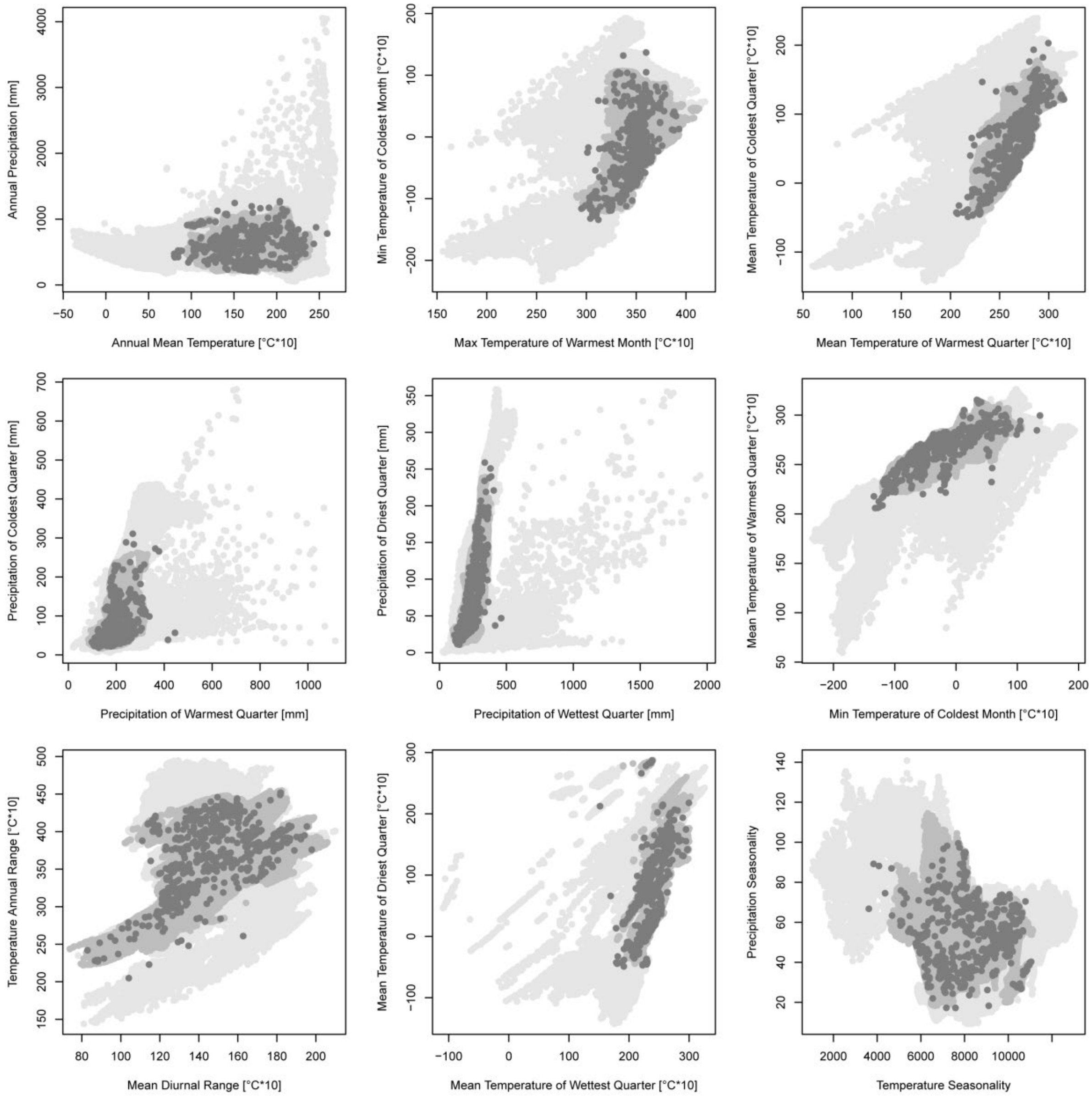

number of samples: 667

number of fossils: 0

sp31 − Kinosternon herrerai

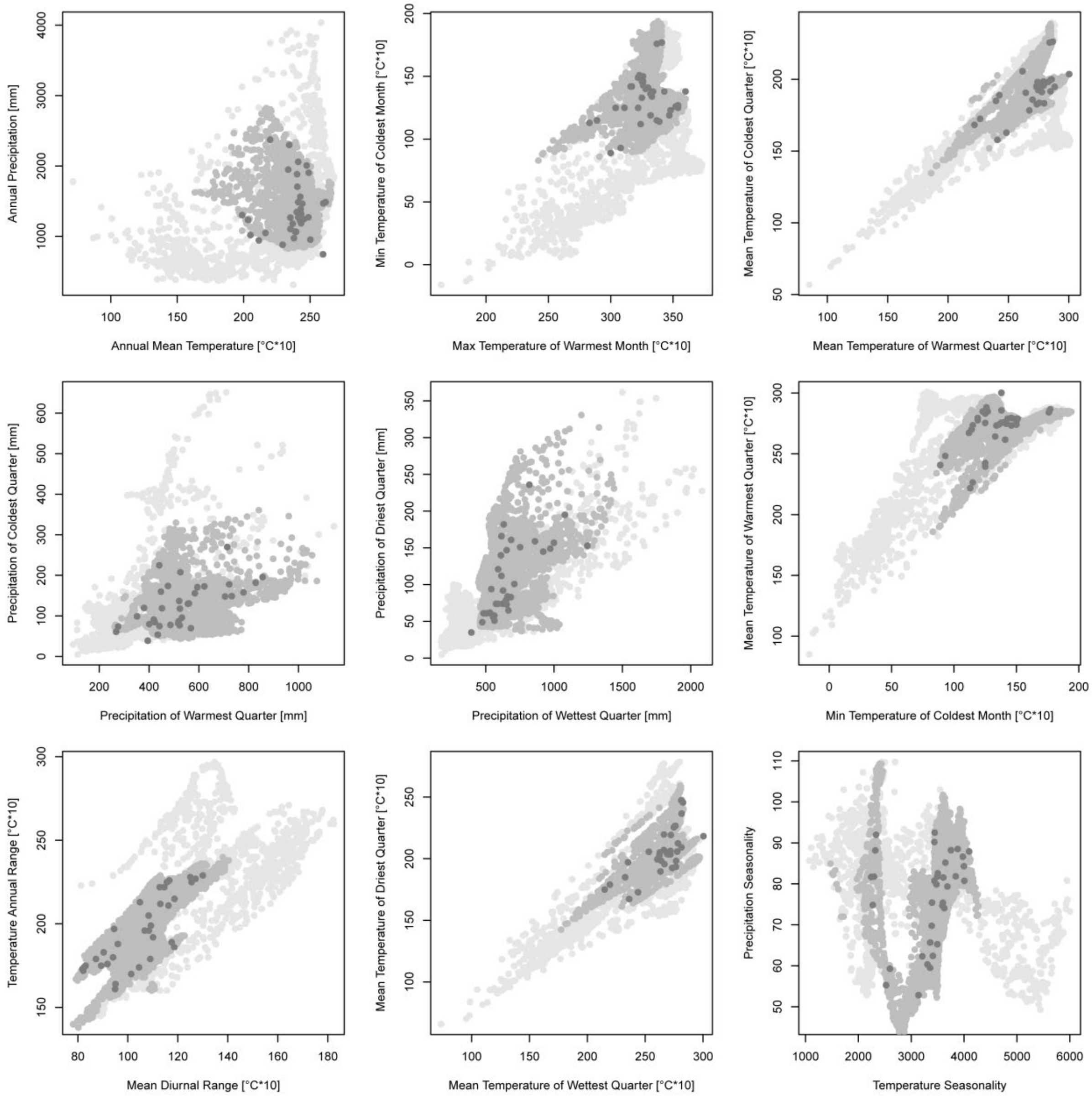

number of samples: 36

number of fossils: 0

sp32 − Kinosternon hirtipes

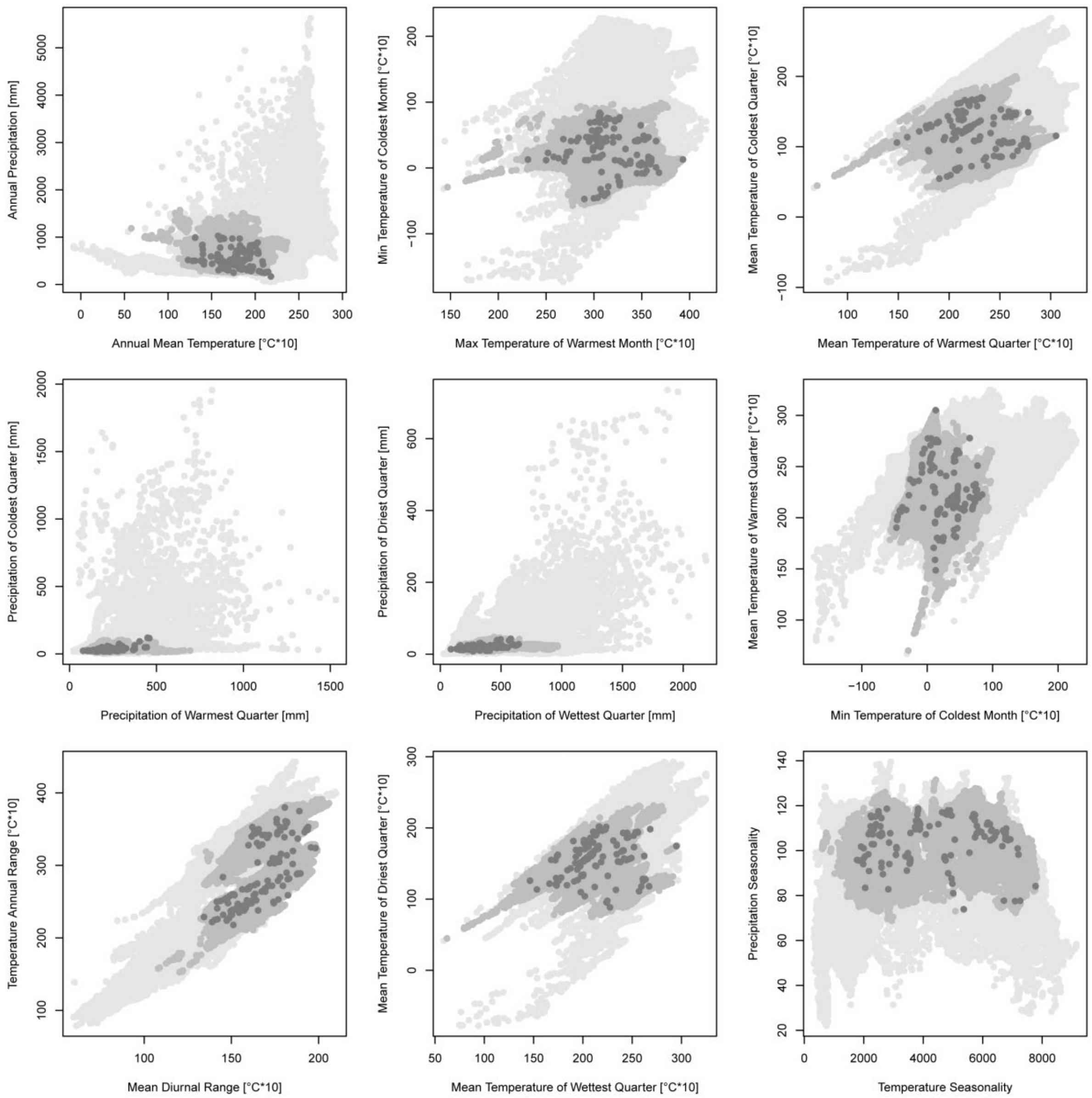

number of samples: 134

number of fossils: 0

sp33 − Kinosternon integrum

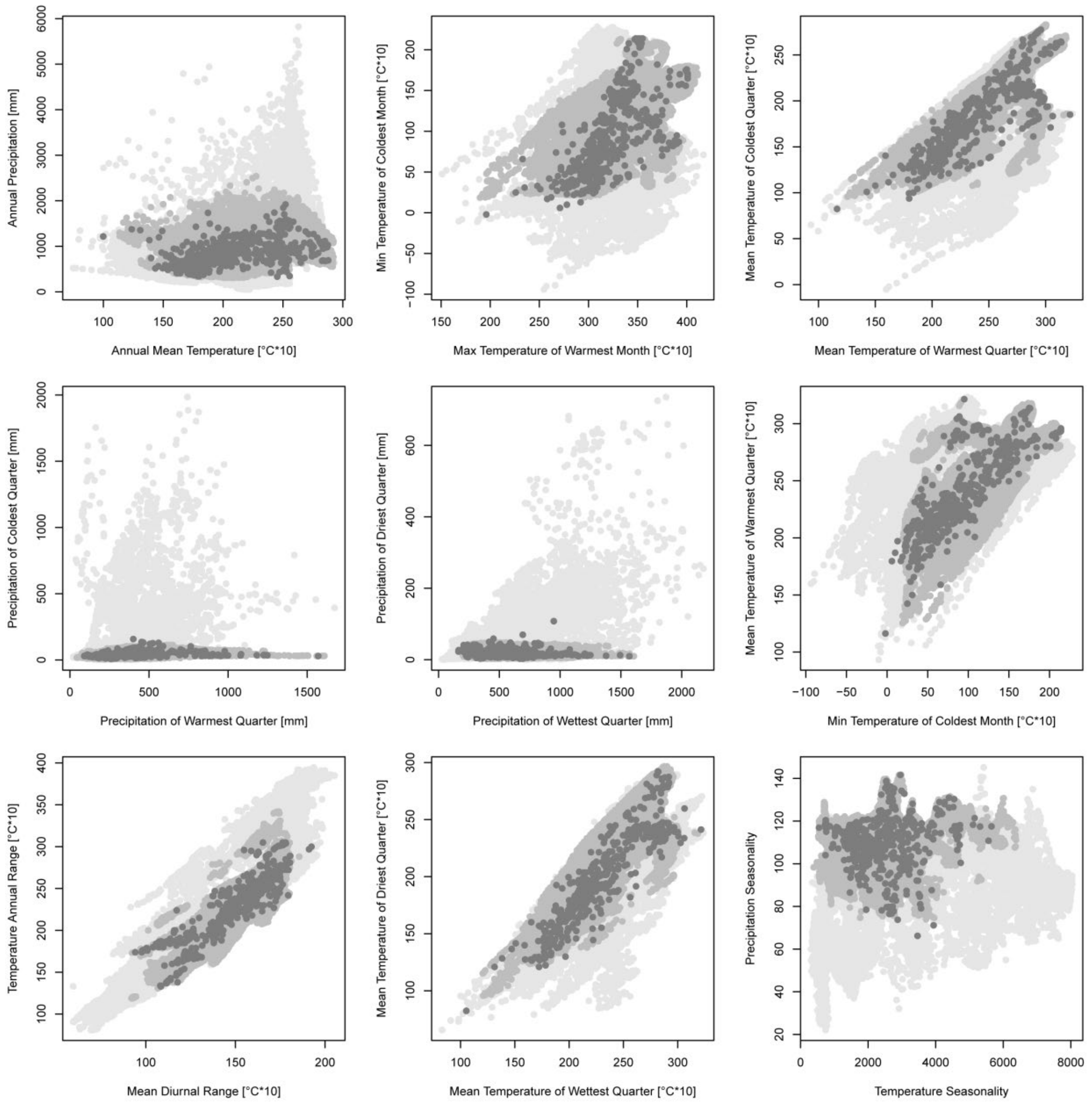

number of samples: 545

number of fossils: 0

sp34 − Kinosternon sonoriense

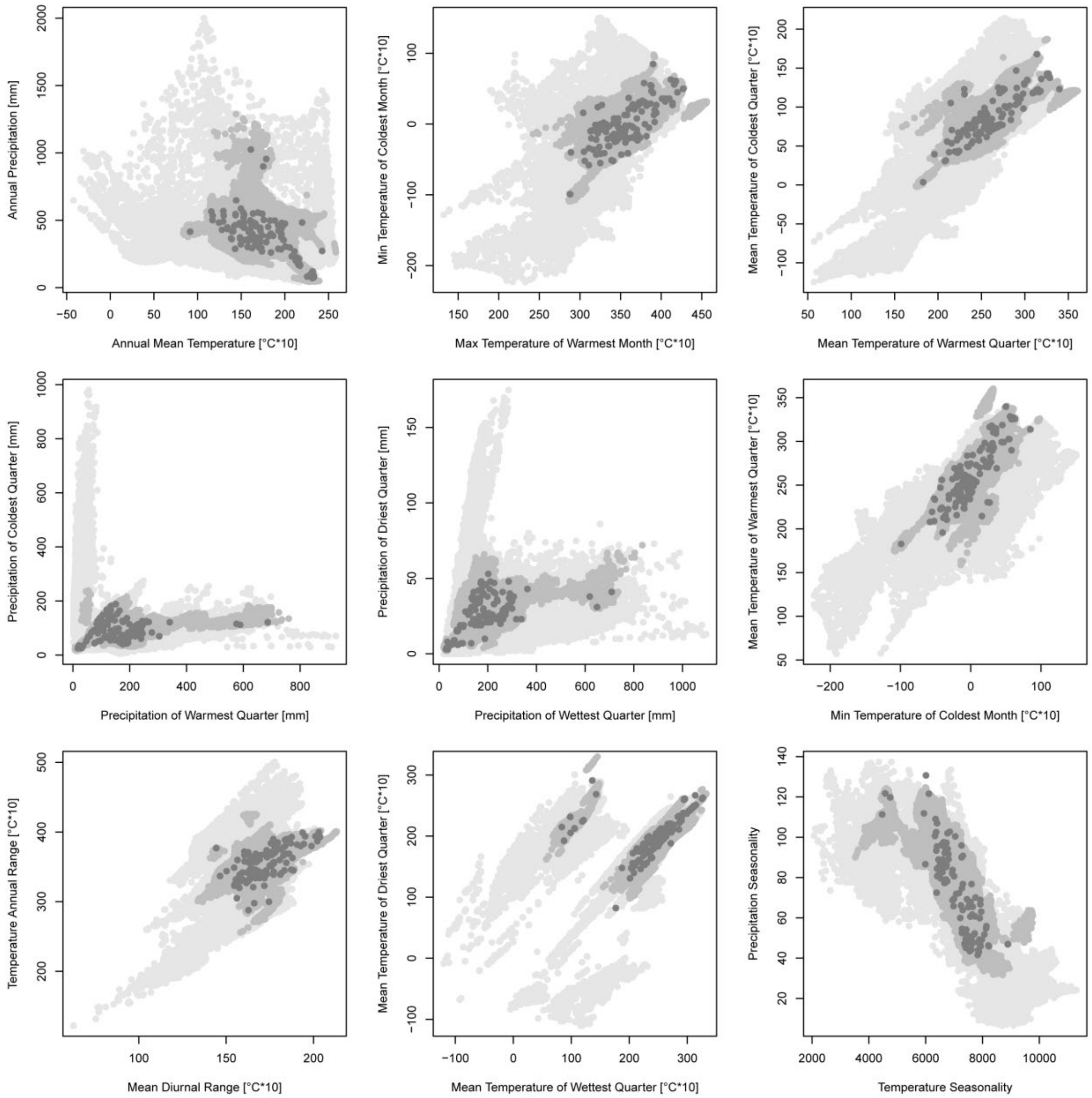

number of samples: 132

number of fossils: 0

sp35 − Kinosternon subrubrum

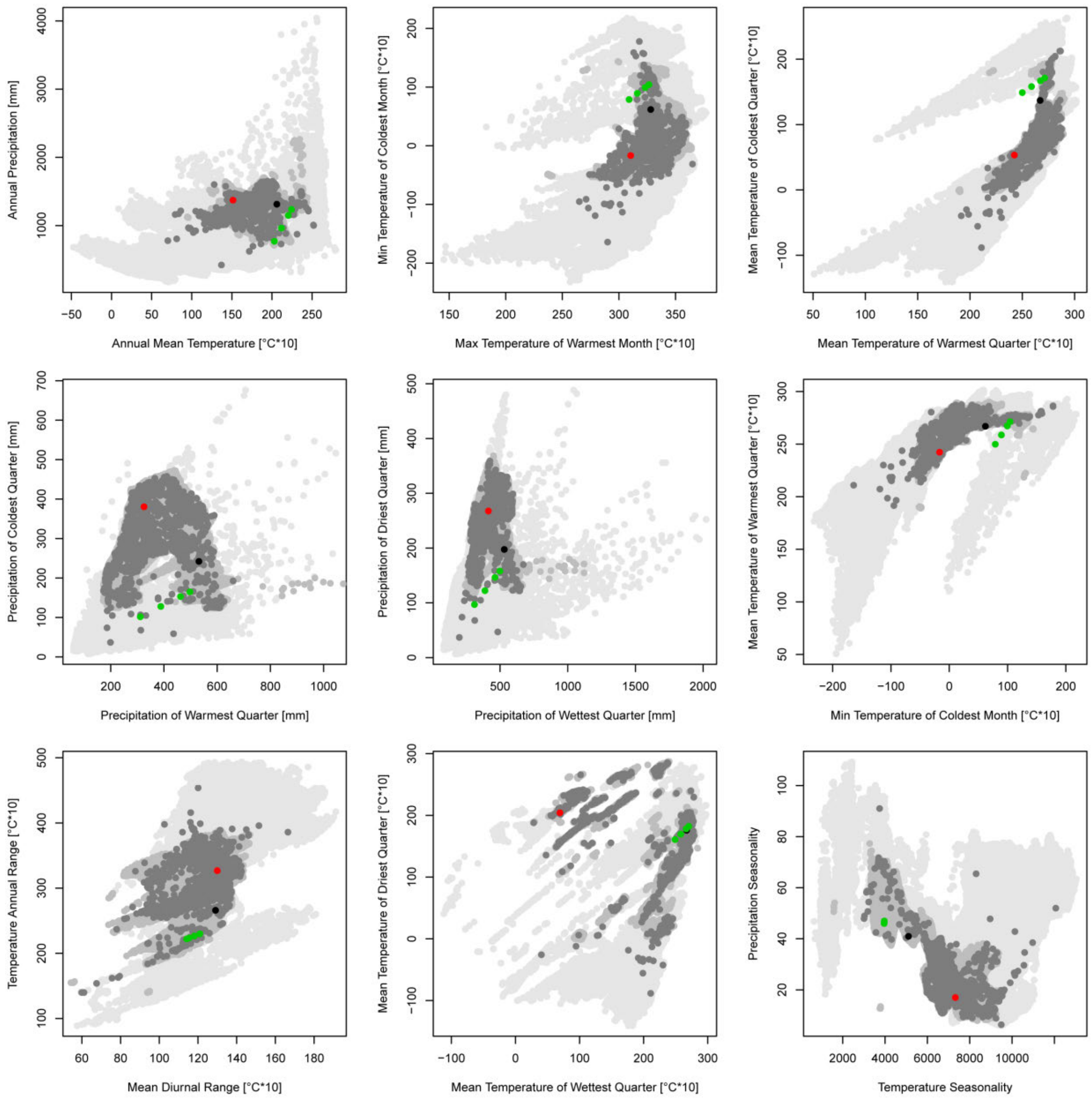

number of samples: 1581

number of fossils: 3

sp36 − Macrochelys temminckii

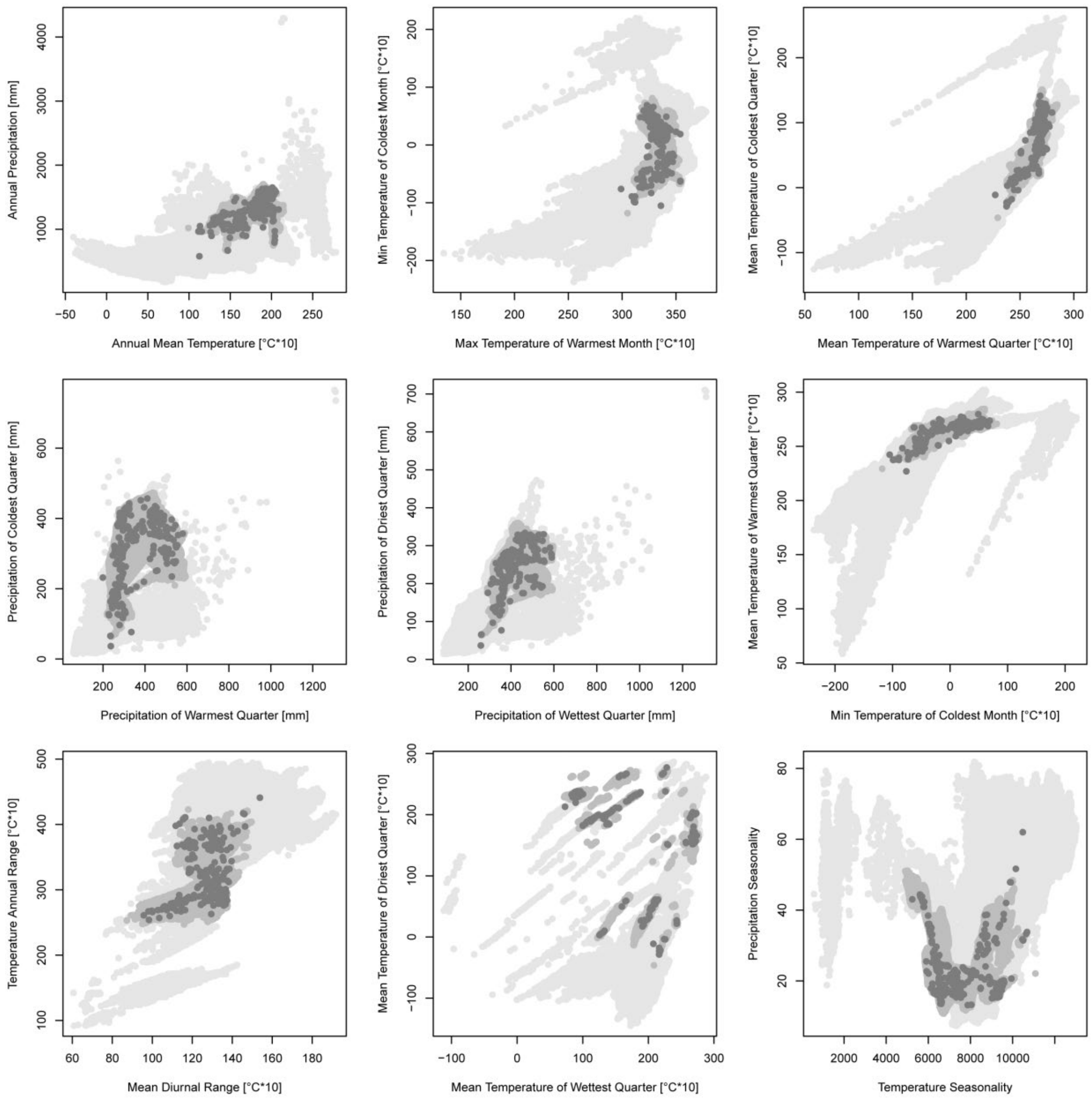

number of samples: 271

number of fossils: 0

sp37 − Malaclemys terrapin

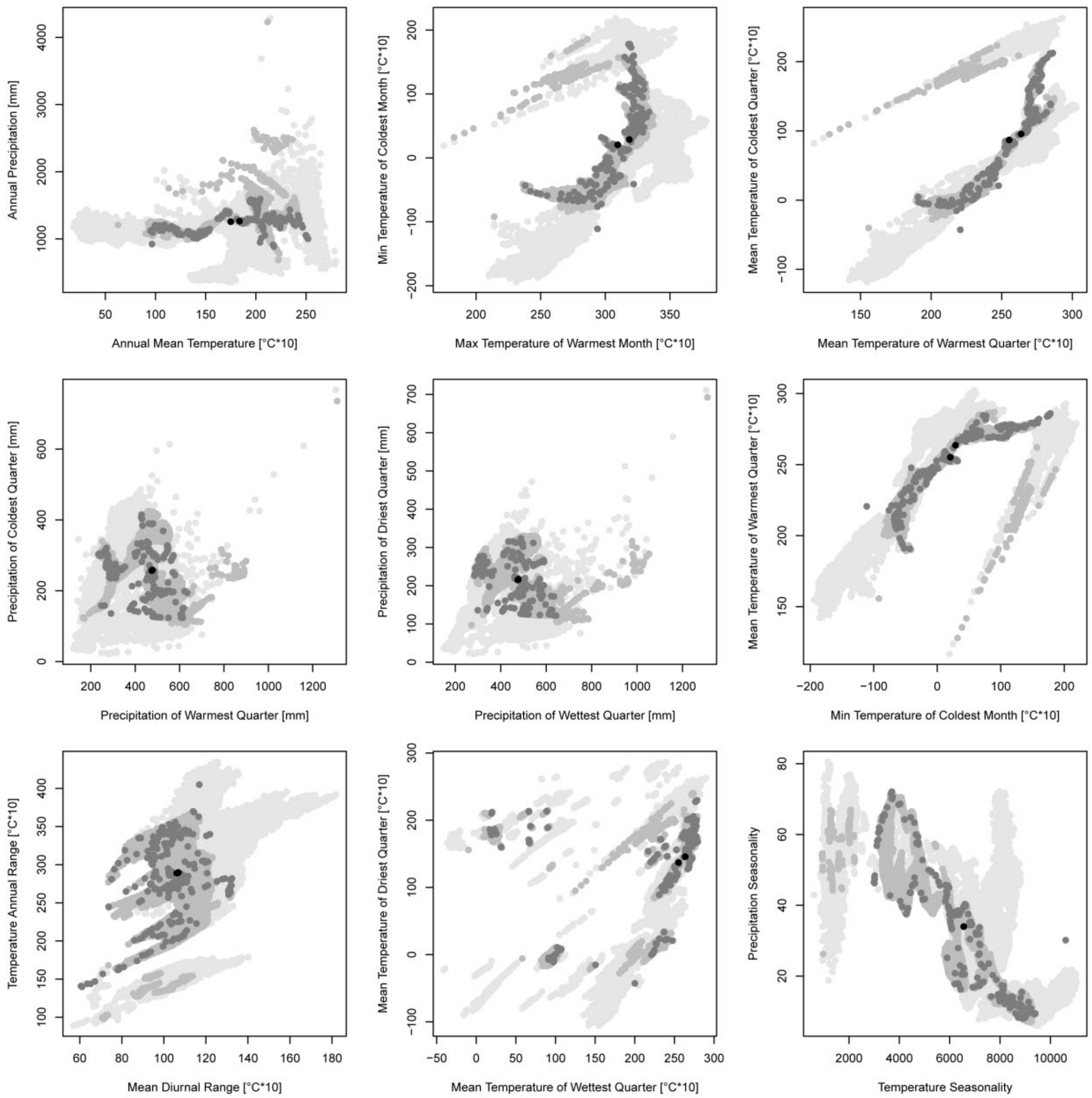

number of samples: 240

number of fossils: 1

sp38 − Pseudemys alabamensis

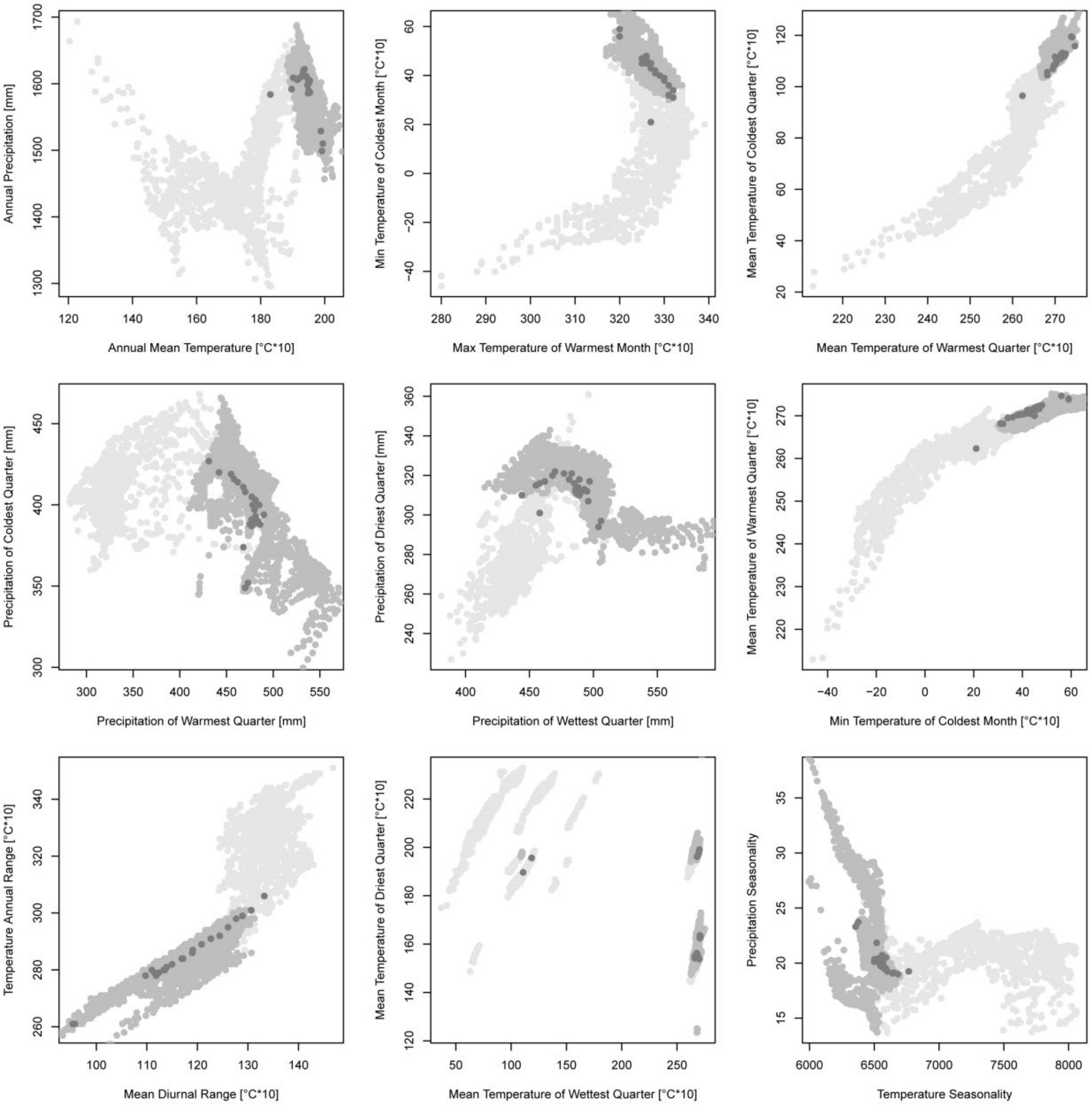

number of samples: 34

number of fossils: 0

sp39 − Pseudemys concinna

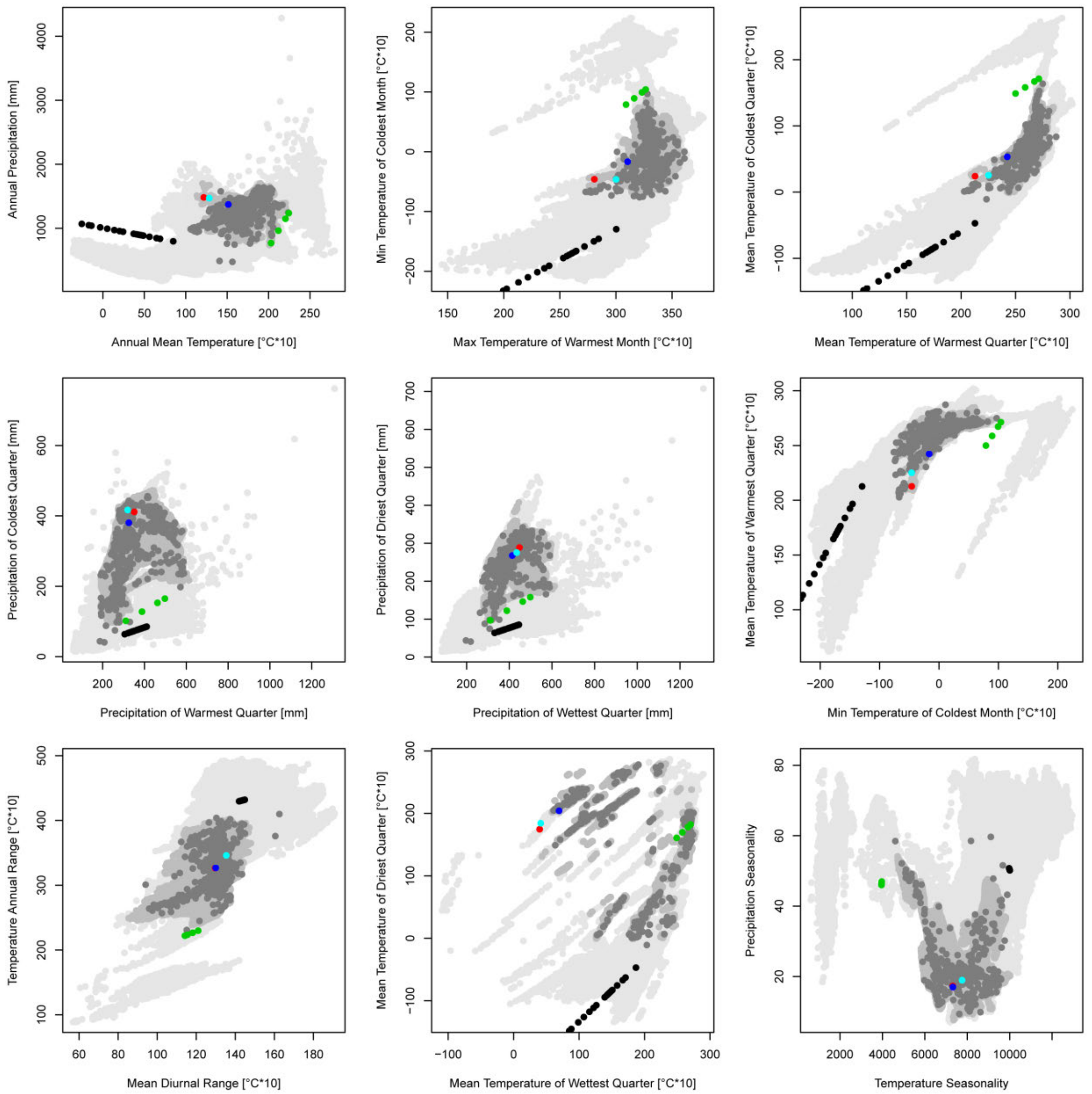

number of samples: 551

number of fossils: 5

sp40 − Pseudemys floridana

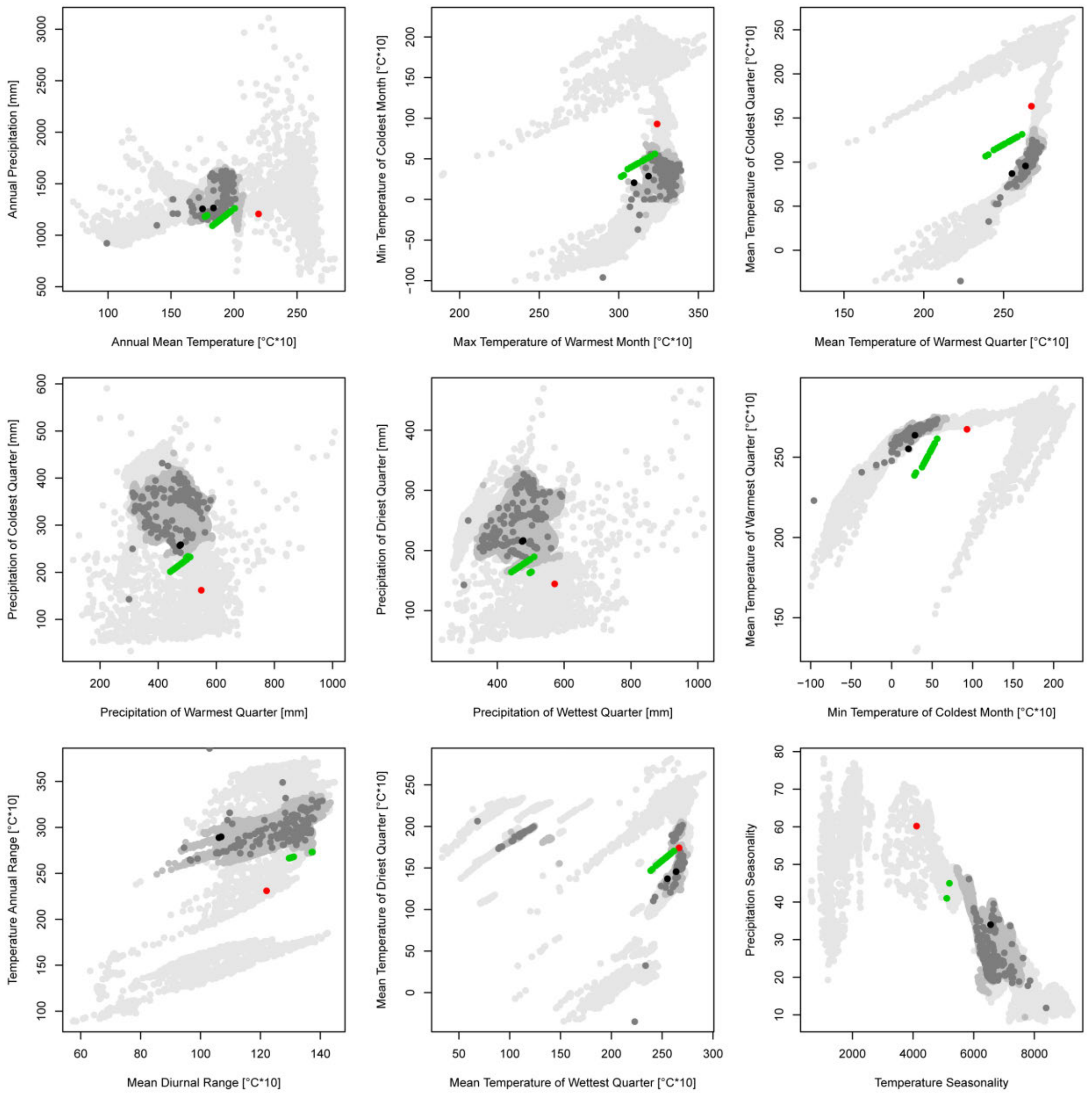

number of samples: 162

number of fossils: 3

sp41 − Pseudemys gorzugi

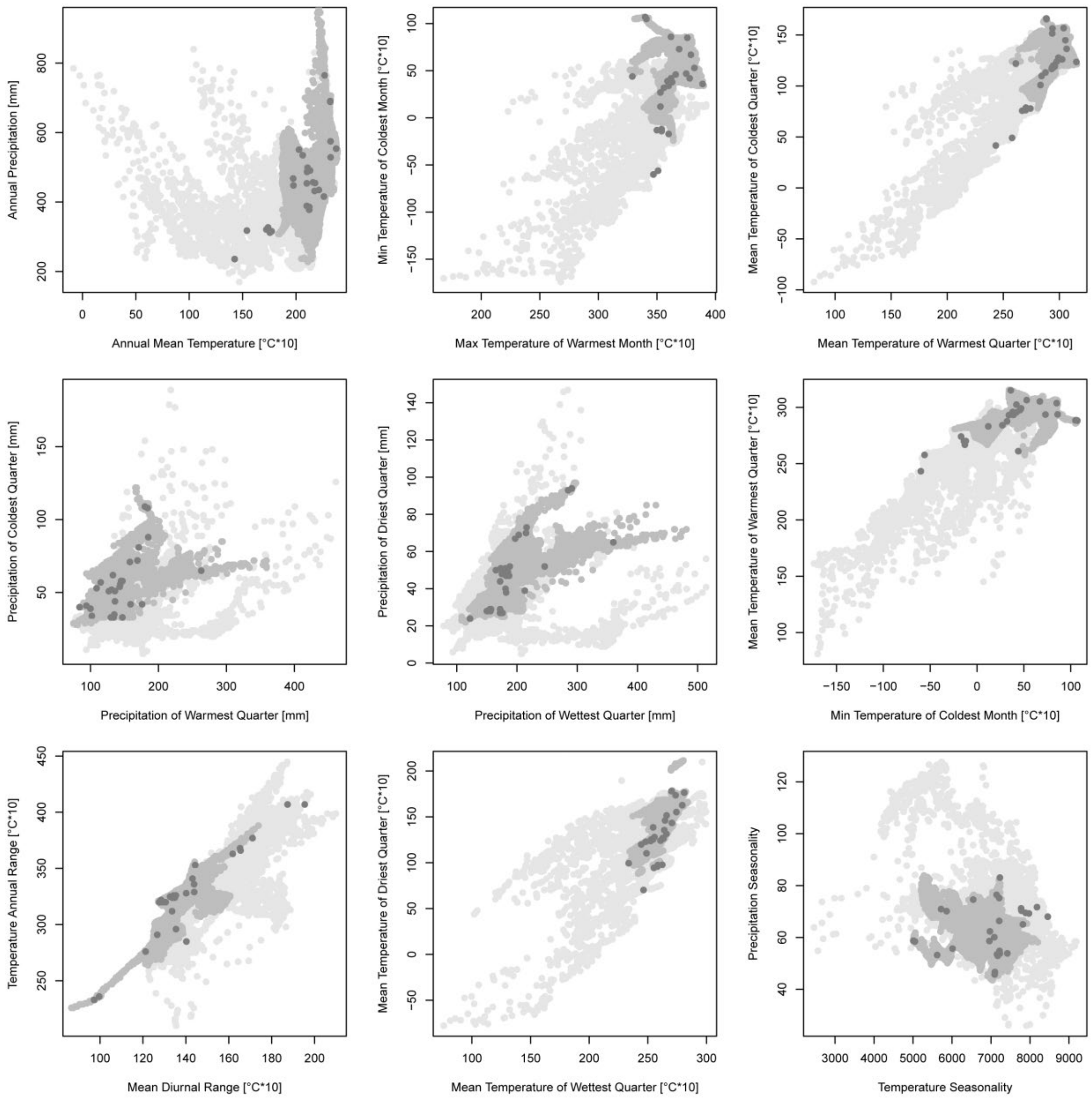

number of samples: 31

number of fossils: 0

sp42 − Pseudemys nelsoni

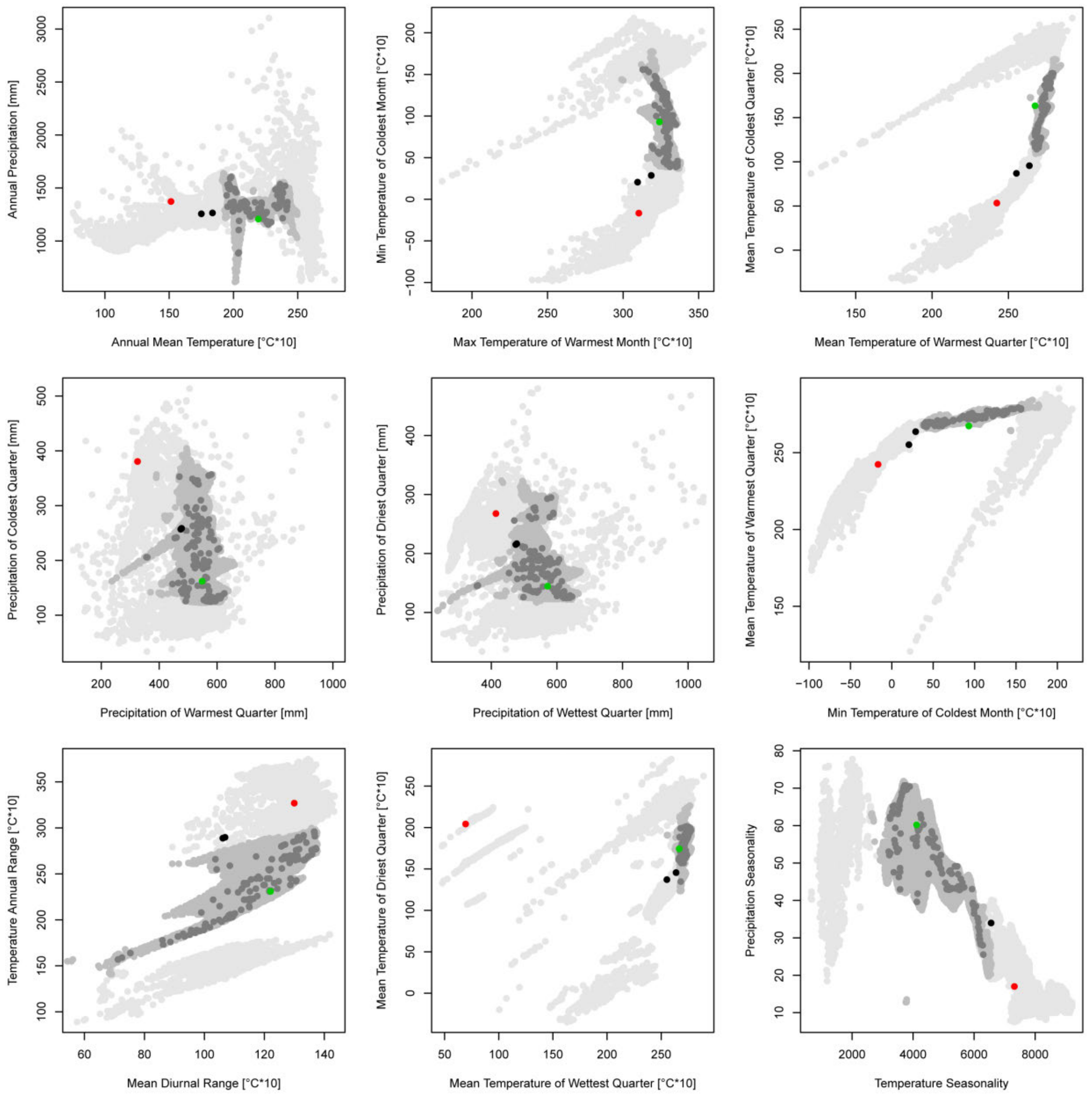

number of samples: 149

number of fossils: 3

sp43 − Pseudemys peninsularis

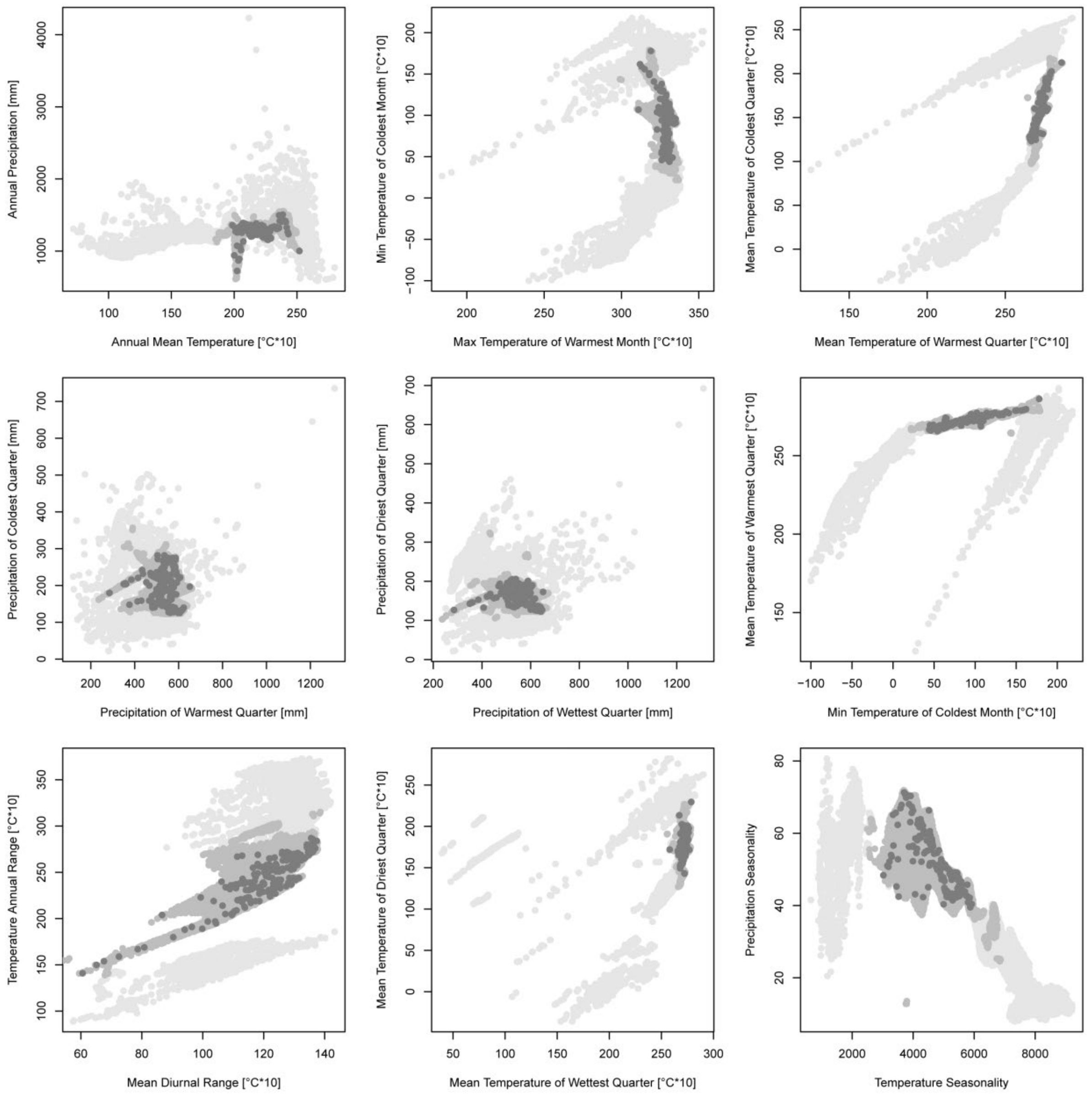

number of samples: 217

number of fossils: 0

sp44 − Pseudemys rubriventris

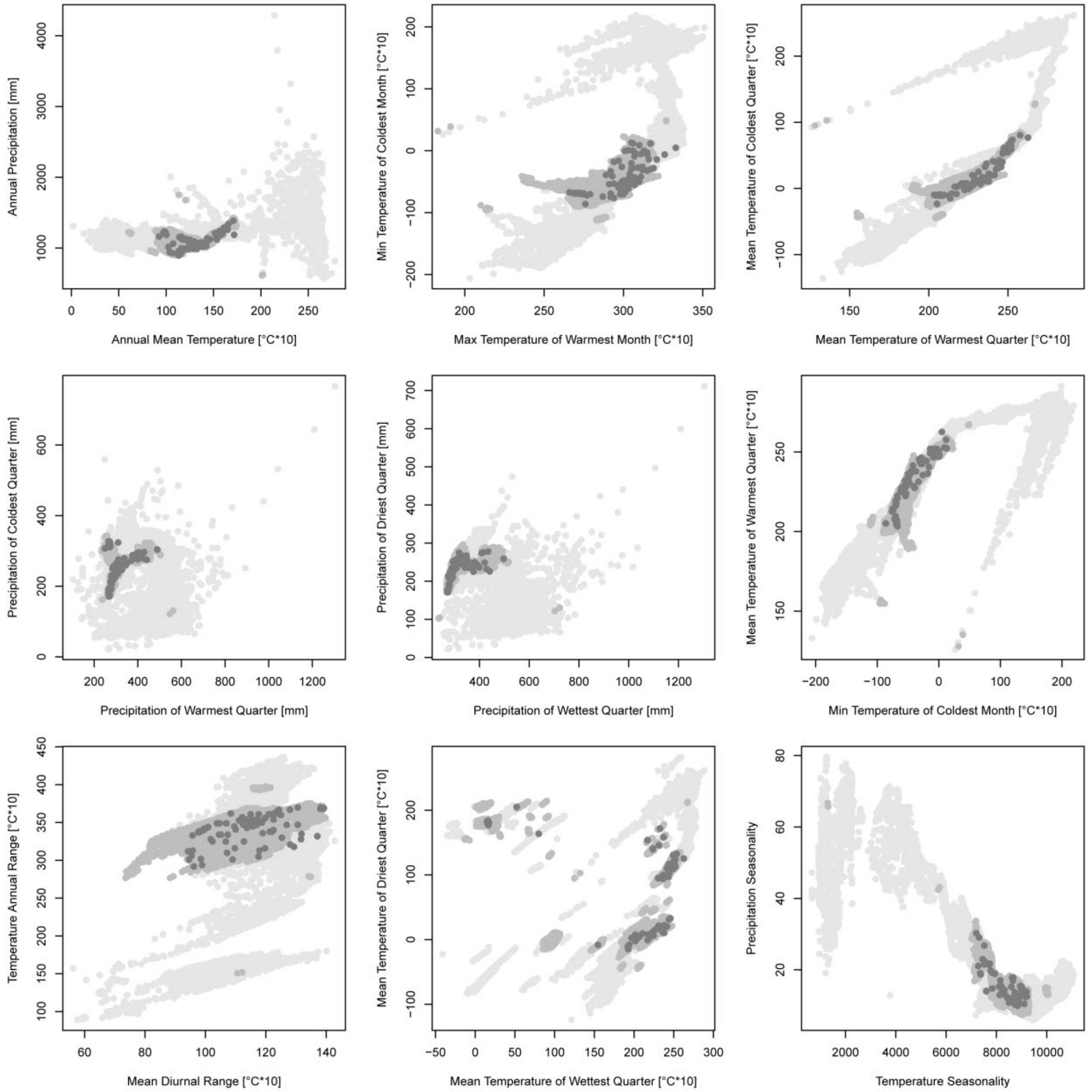

number of samples: 92

number of fossils: 0

sp45 − Pseudemys texana

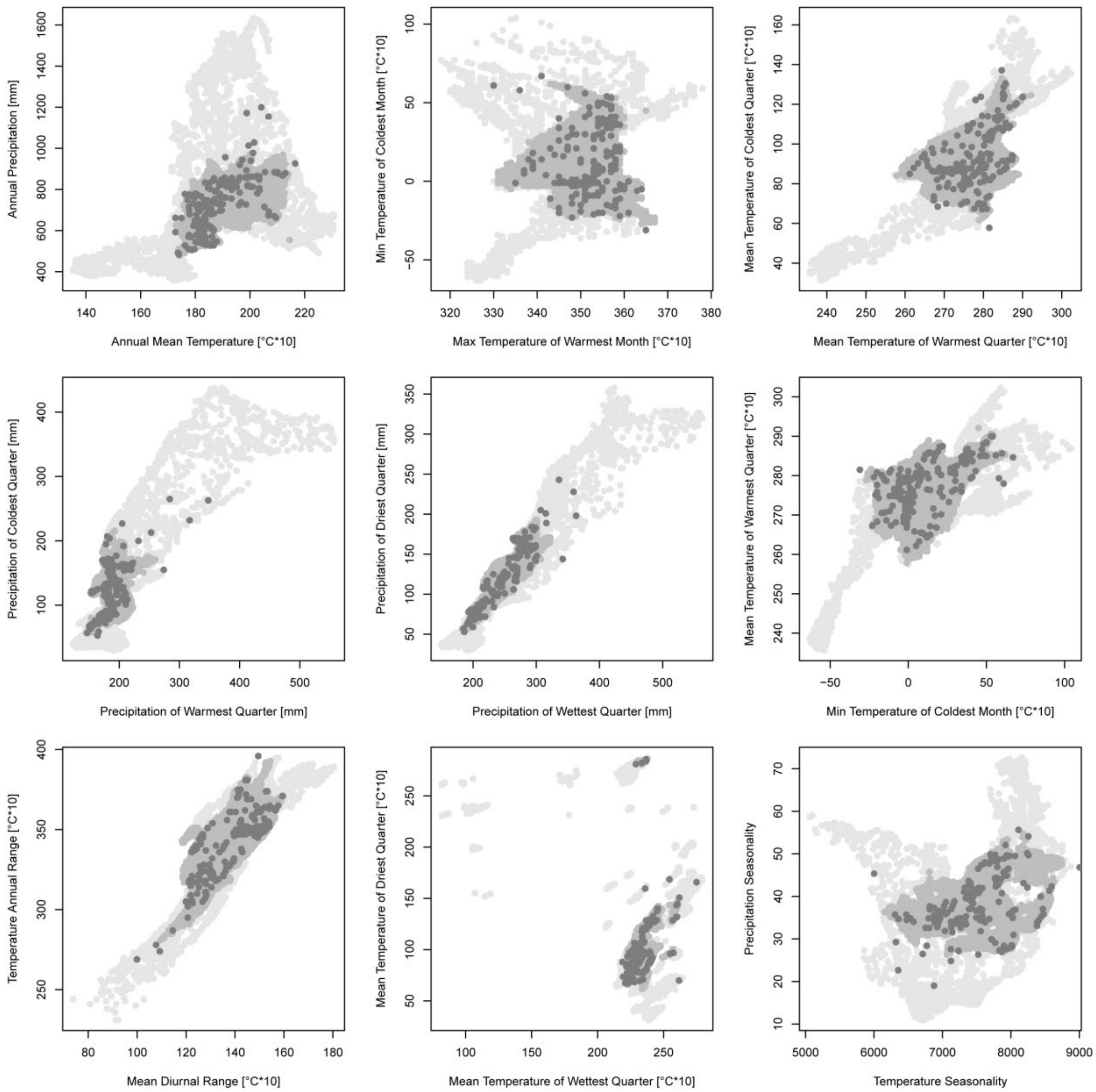

number of samples: 212

number of fossils: 0

sp46 − Rhinoclemmys pulcherrima

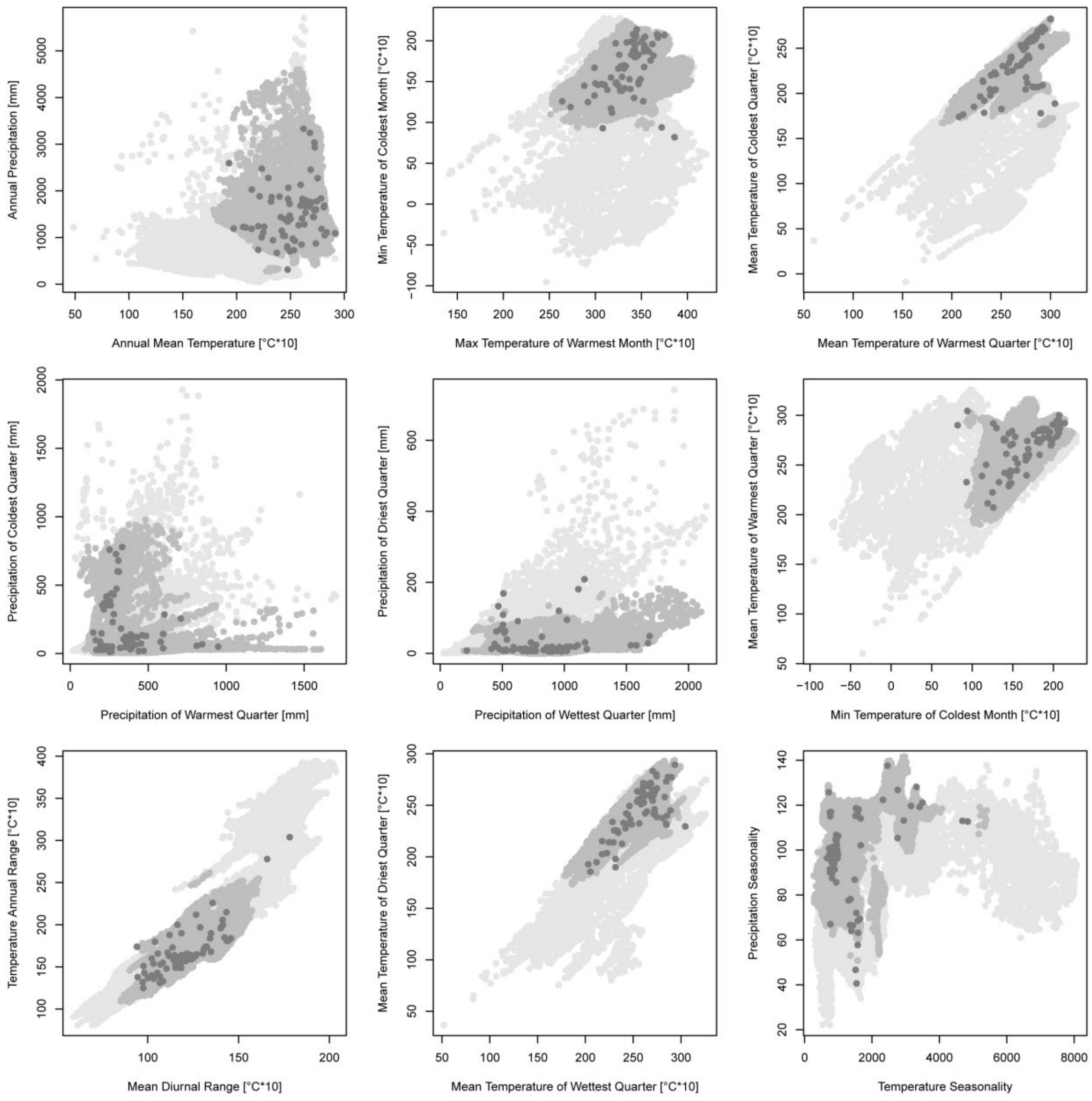

number of samples: 76

number of fossils: 0

sp47 − Sternotherus carinatus

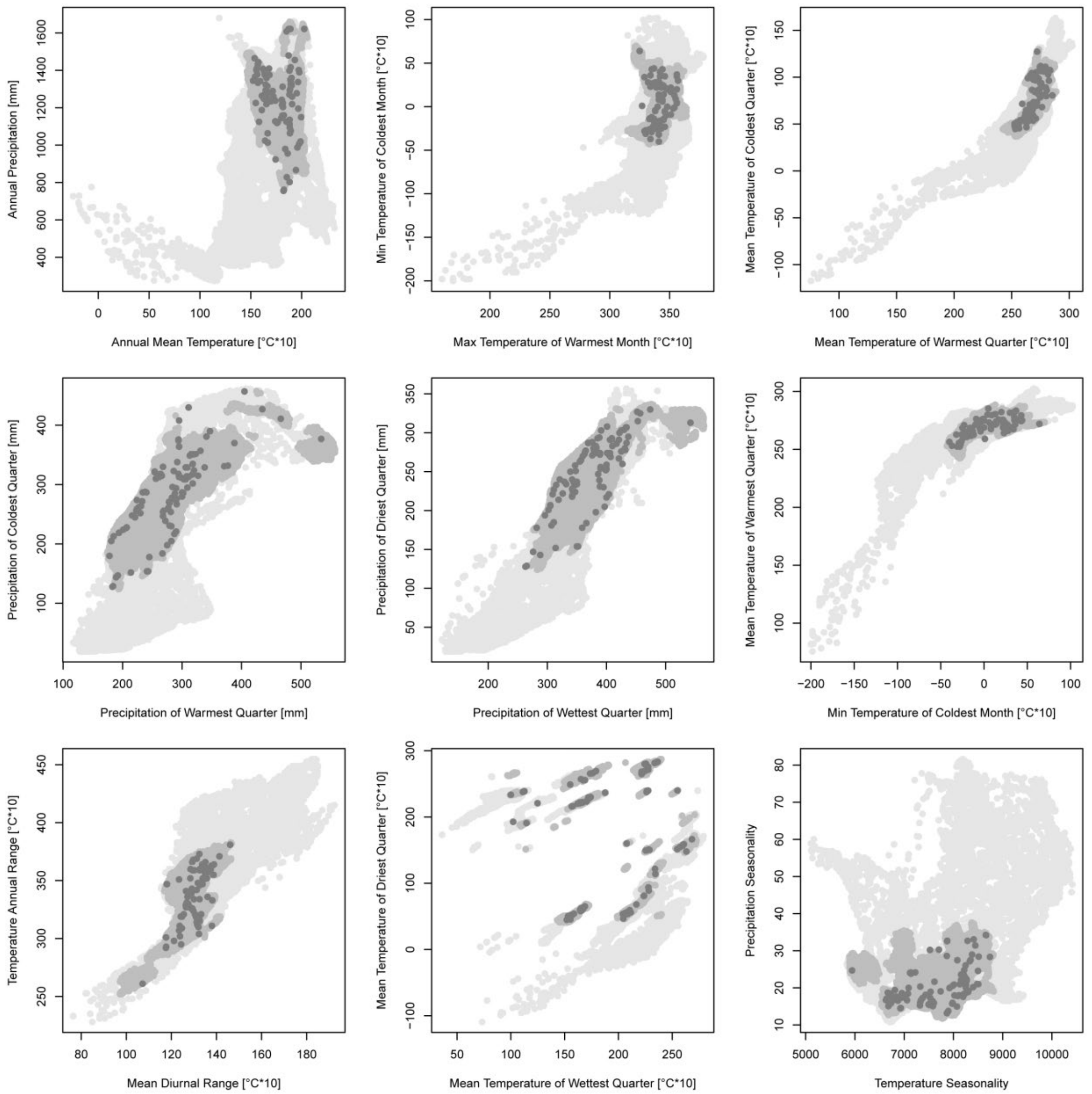

number of samples: 105

number of fossils: 0

sp48 − Sternotherus depressus

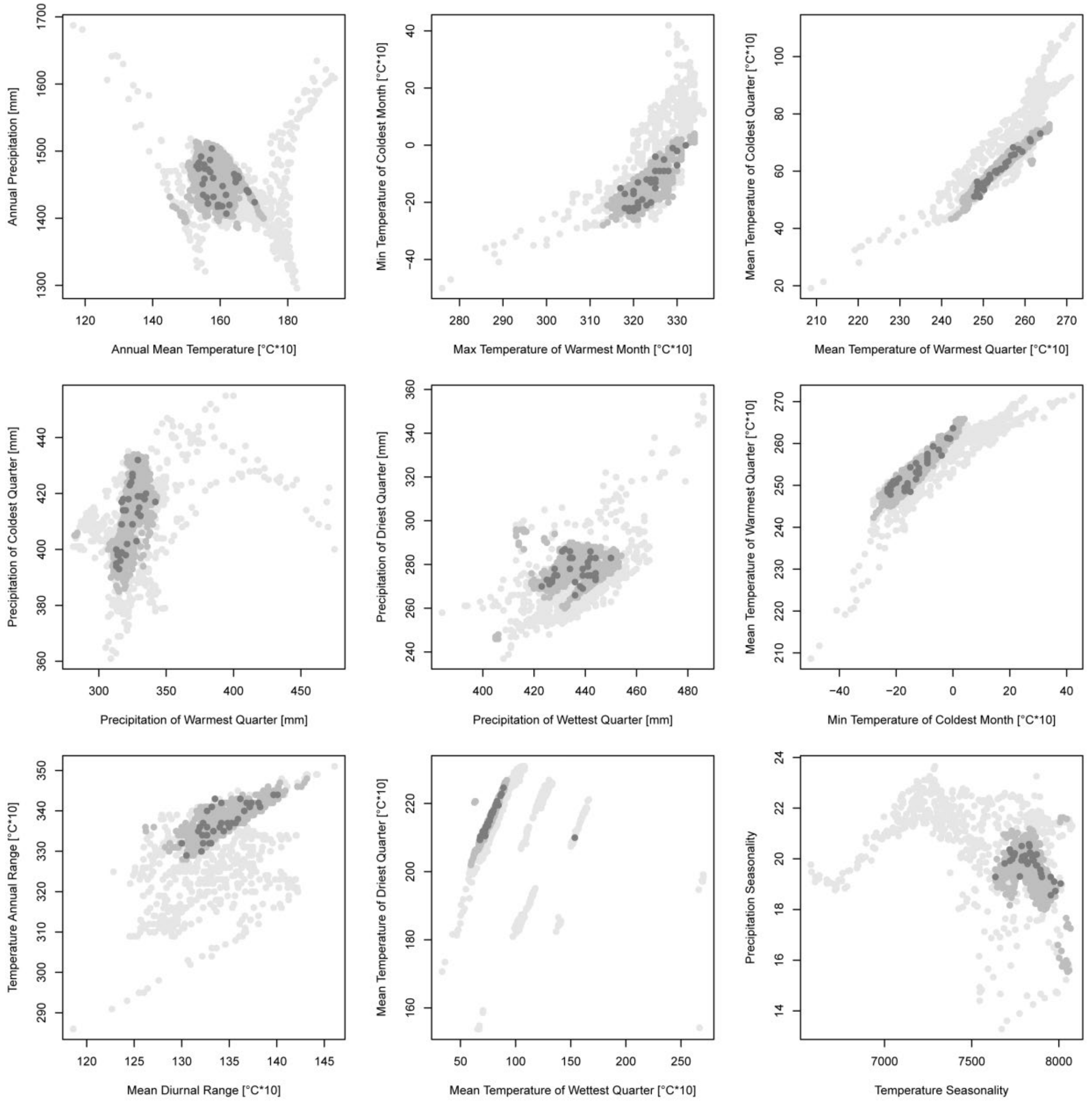

number of samples: 44

number of fossils: 0

sp49 − Sternotherus minor

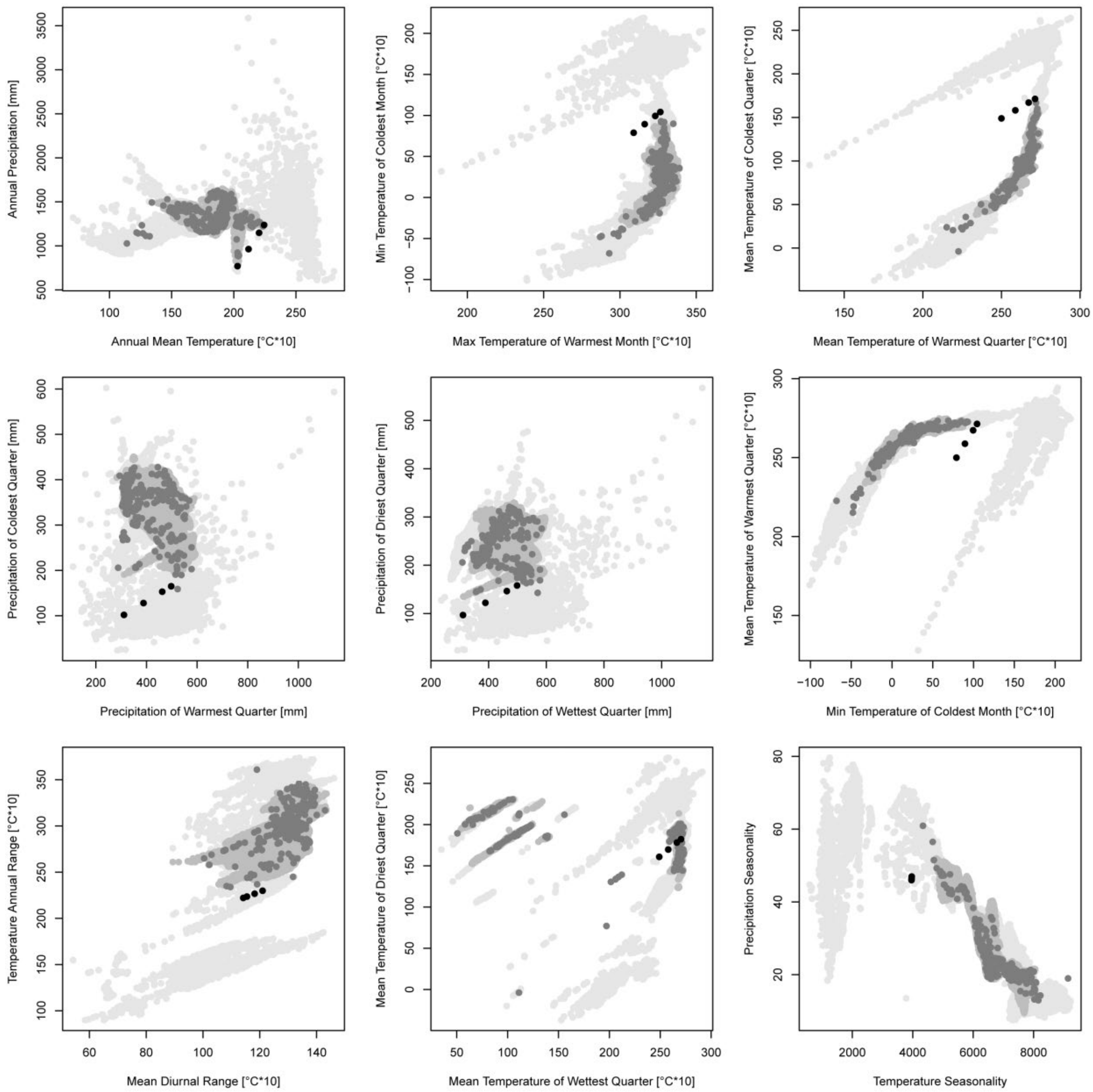

number of samples: 257

number of fossils: 1

sp50 − Sternotherus odoratus

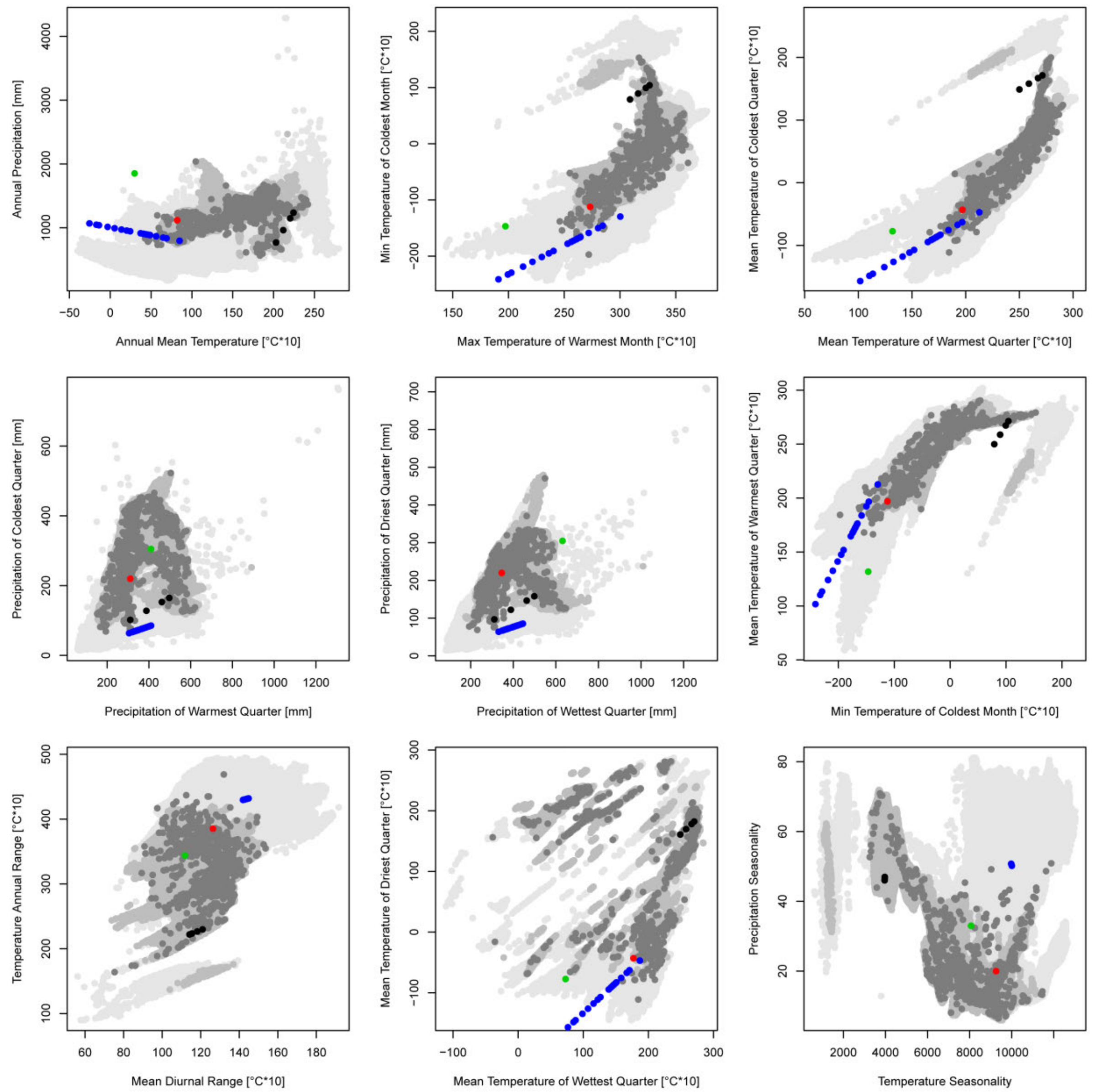

number of samples: 982

number of fossils: 4

sp51 − Terrapene carolina

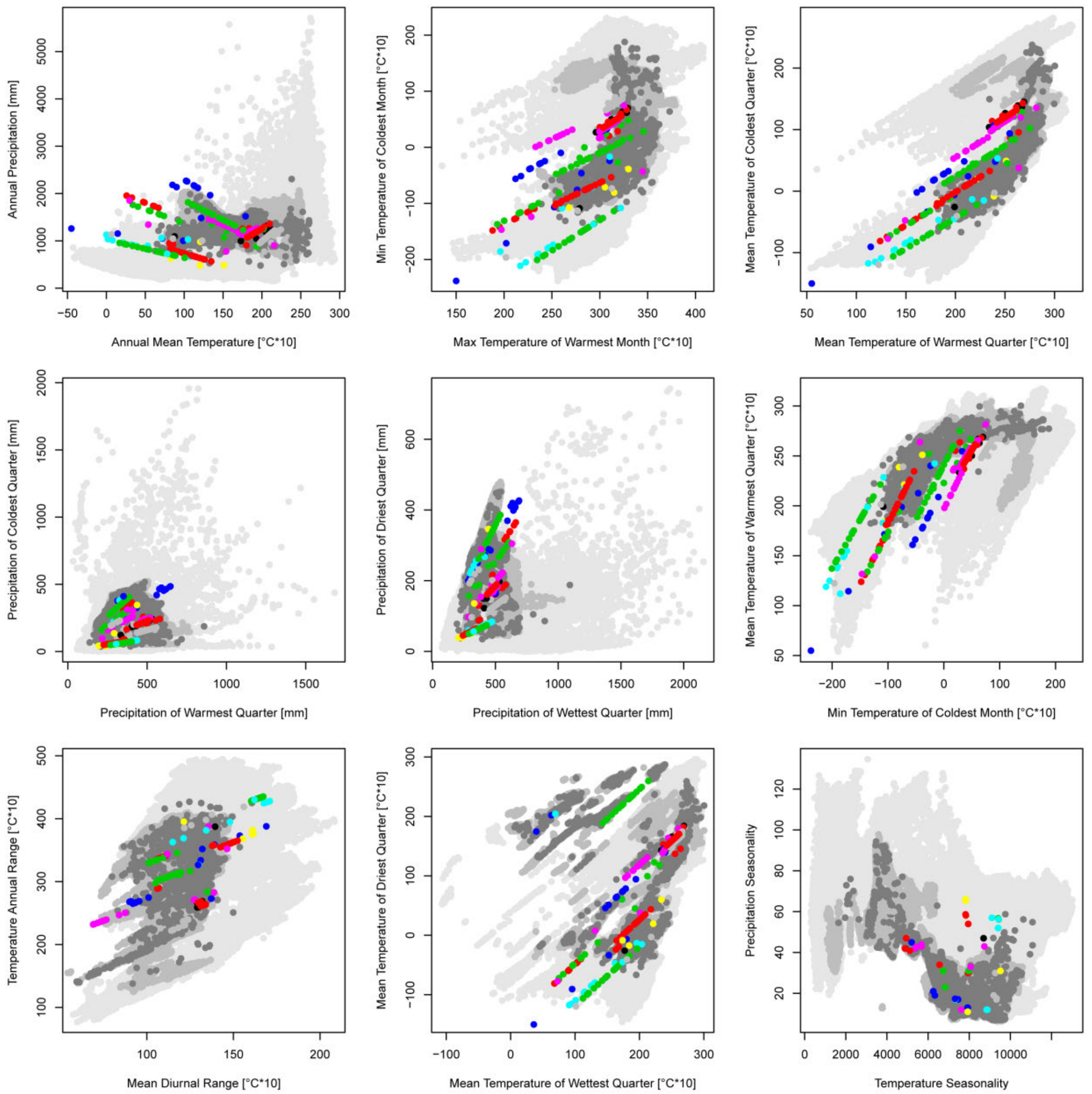

number of samples: 2405

number of fossils: 40

sp52 − Terrapene coahuila

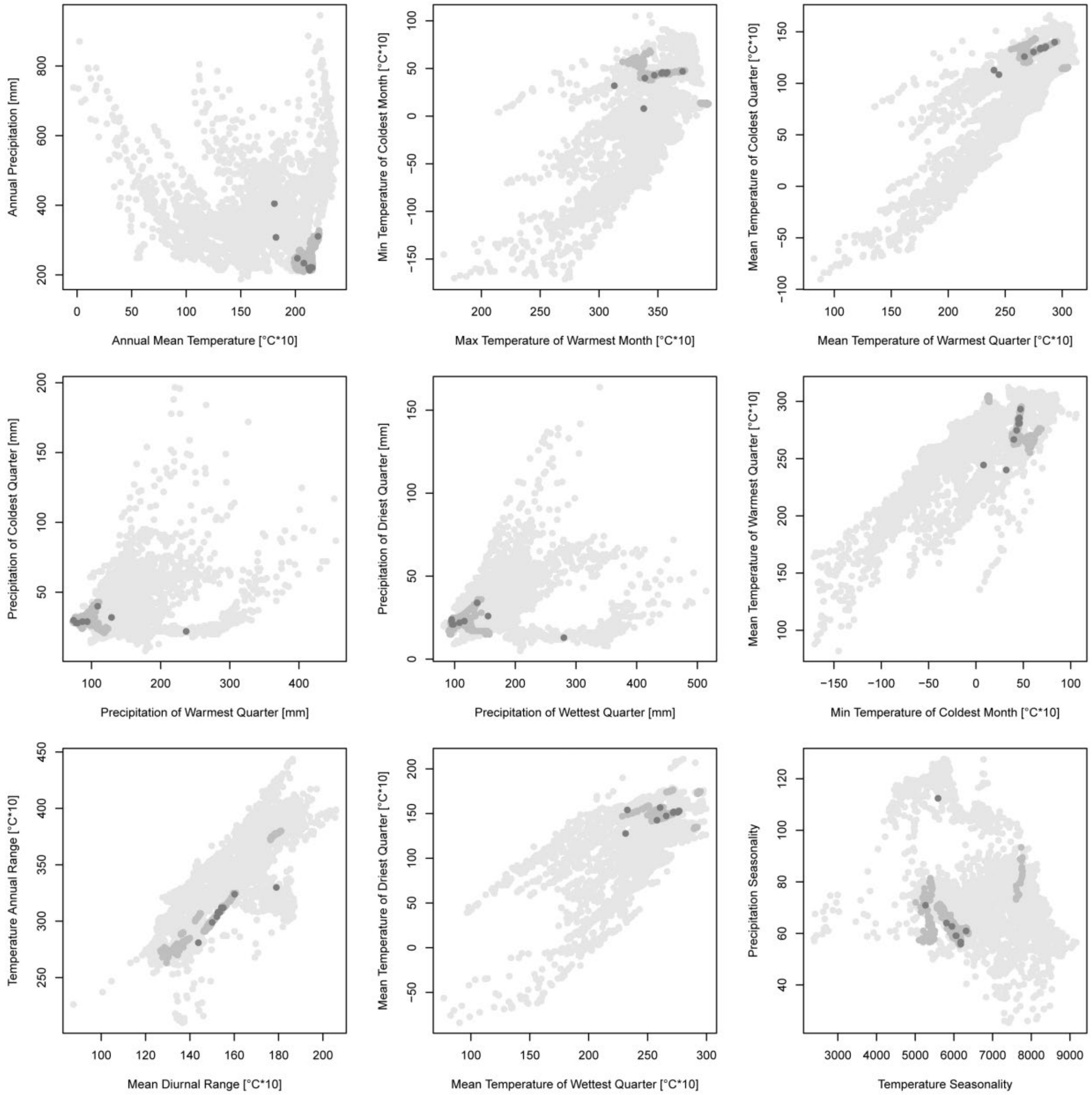

number of samples: 10

number of fossils: 0

sp53 − Terrapene nelsoni

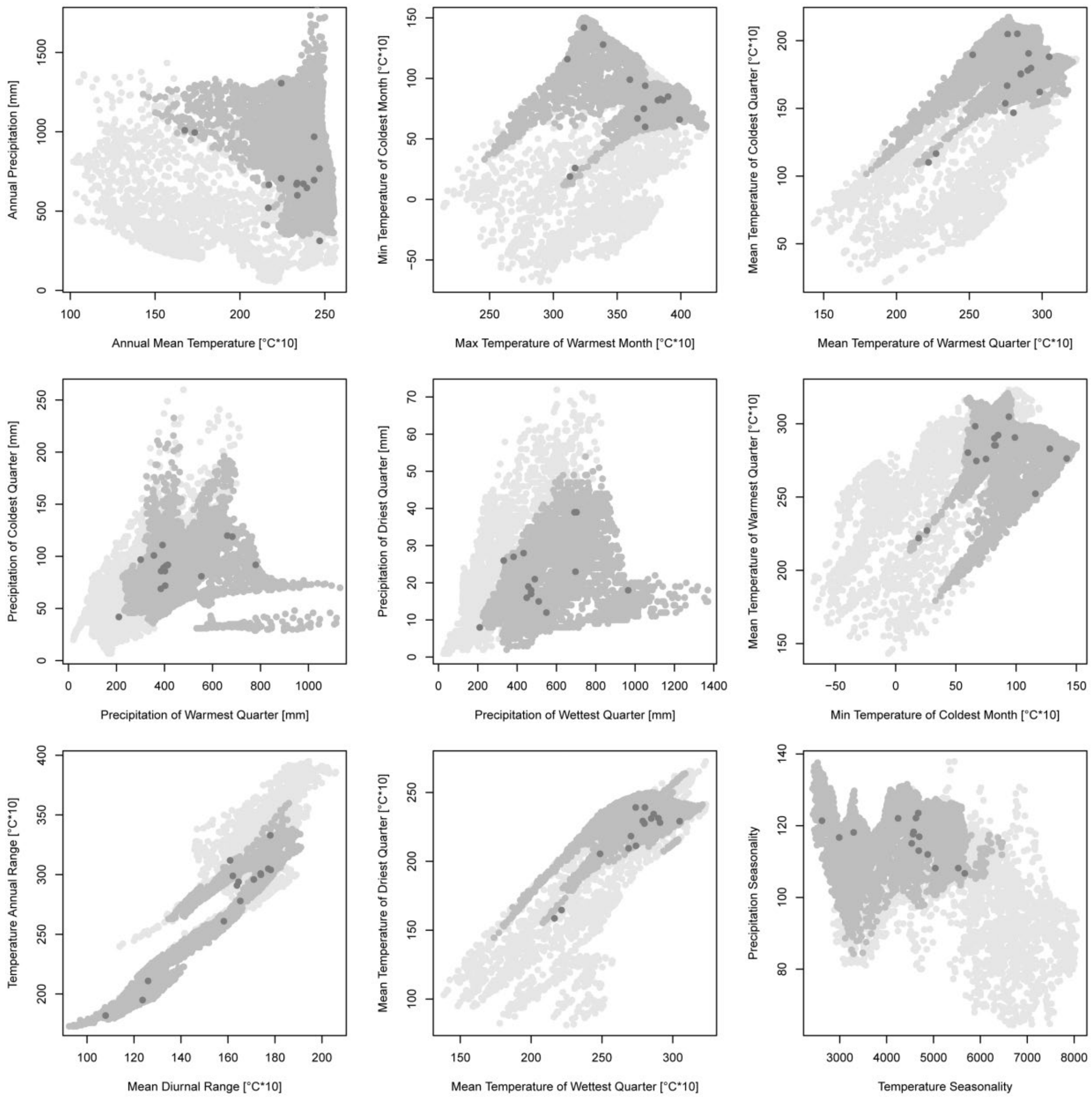

number of samples: 15

number of fossils: 0

sp54 − Terrapene ornata

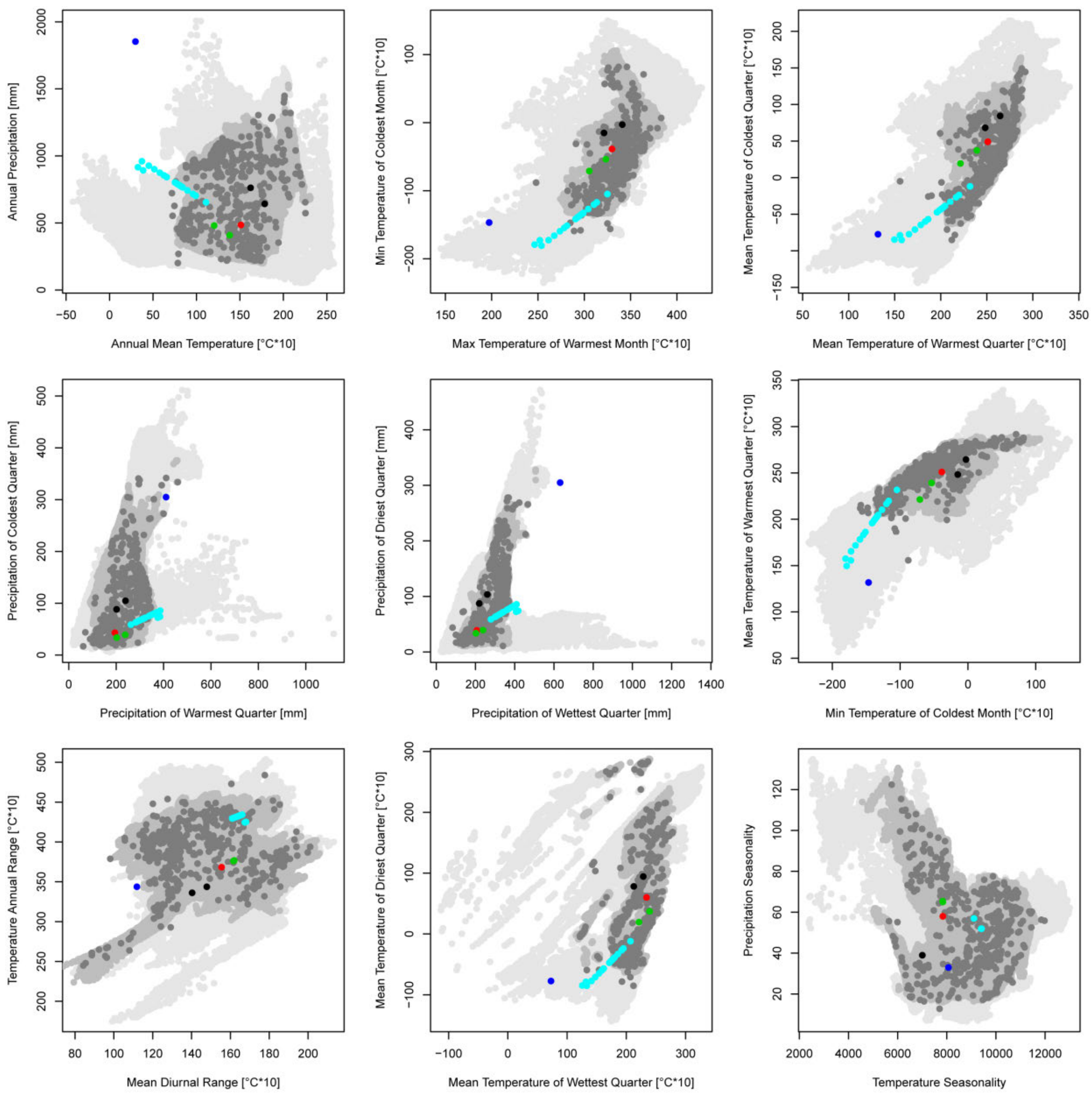

number of samples: 540

number of fossils: 5

sp55 − Trachemys gaigeae

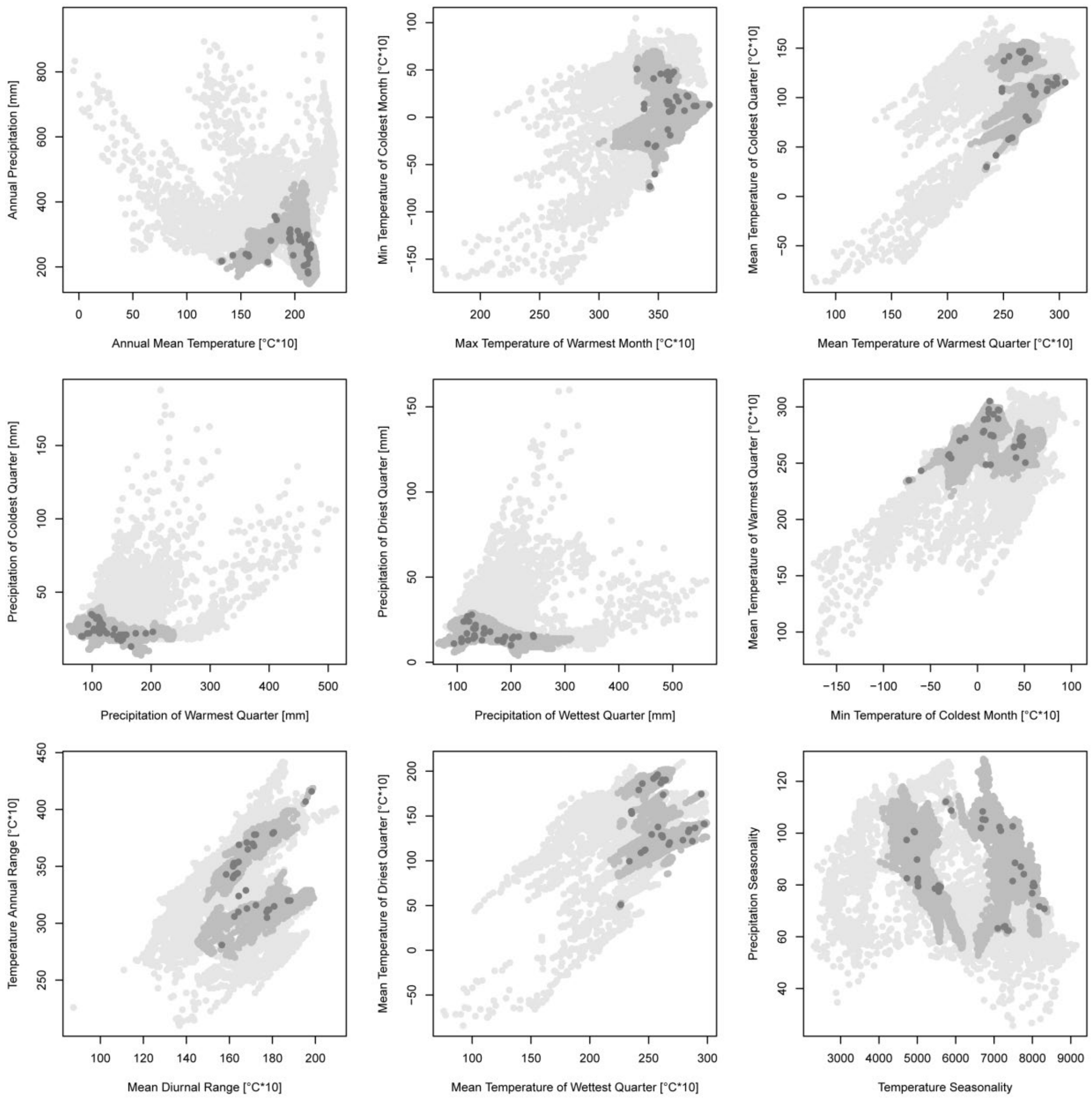

number of samples: 39

number of fossils: 0

sp56 − Trachemys nebulosa

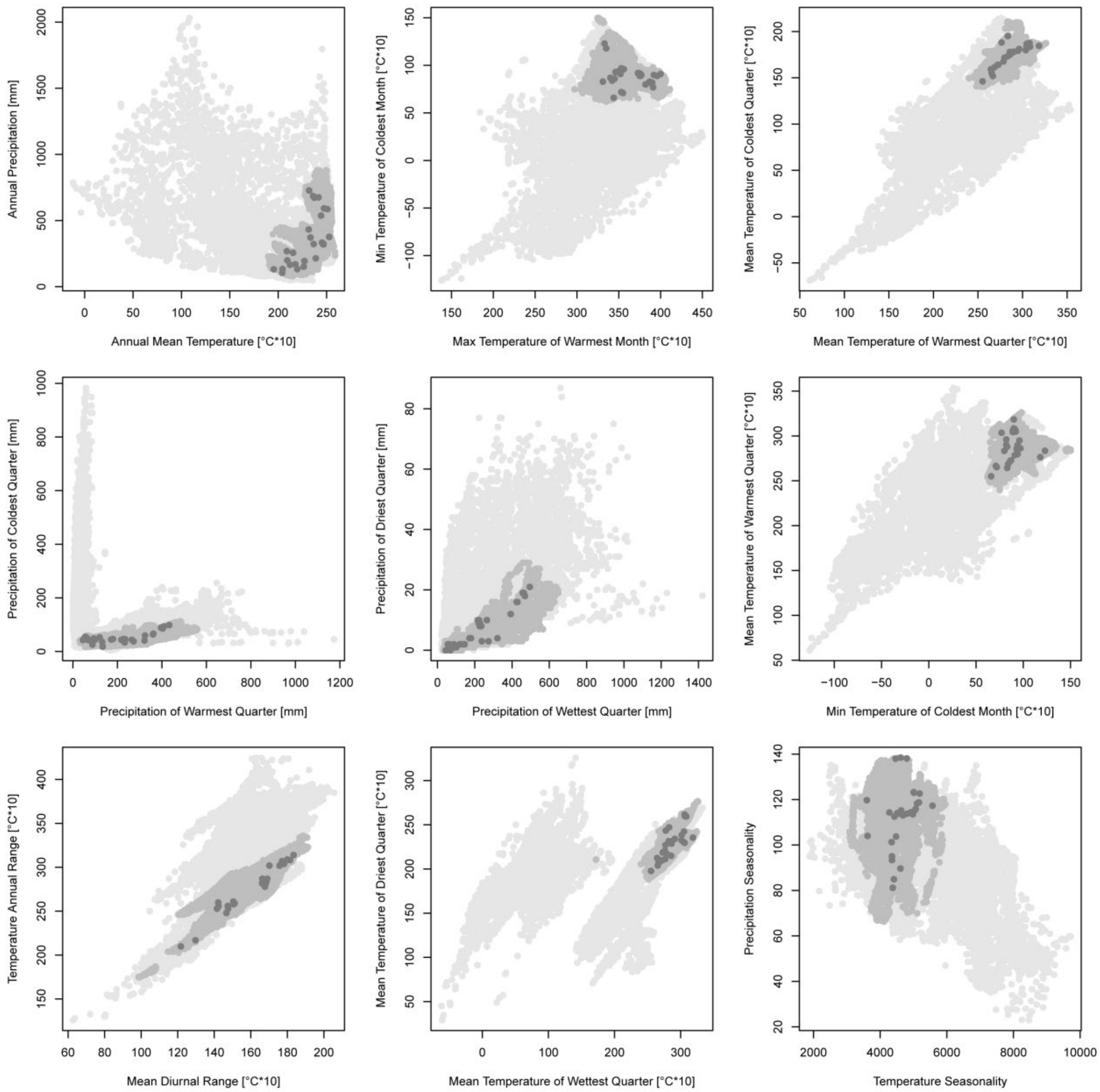

number of samples: 31

number of fossils: 0

sp57 − Trachemys ornata

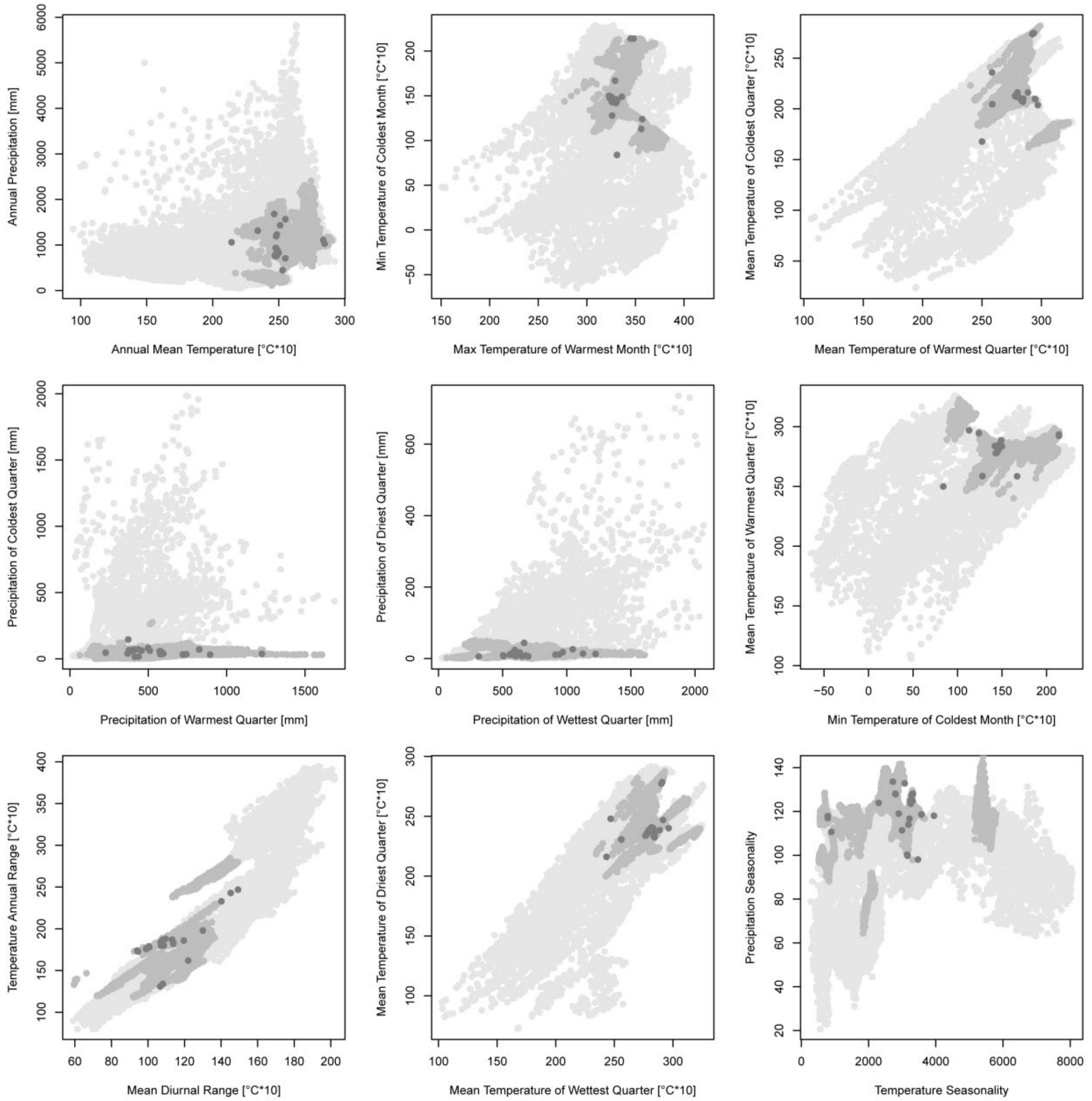

number of samples: 24

number of fossils: 0

sp58 − Trachemys scripta

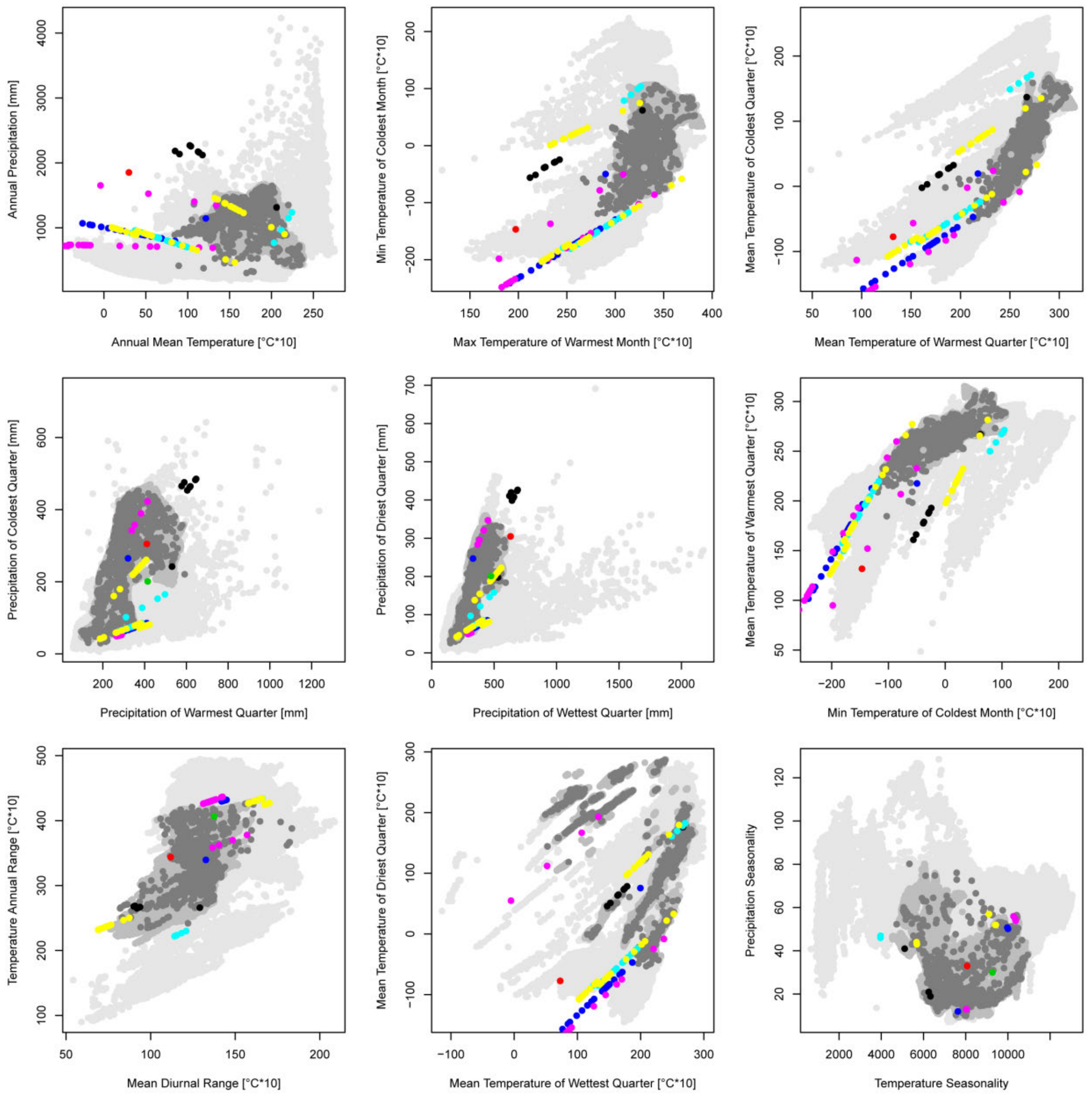

number of samples: 1218

number of fossils: 15

sp59 − Trachemys taylori

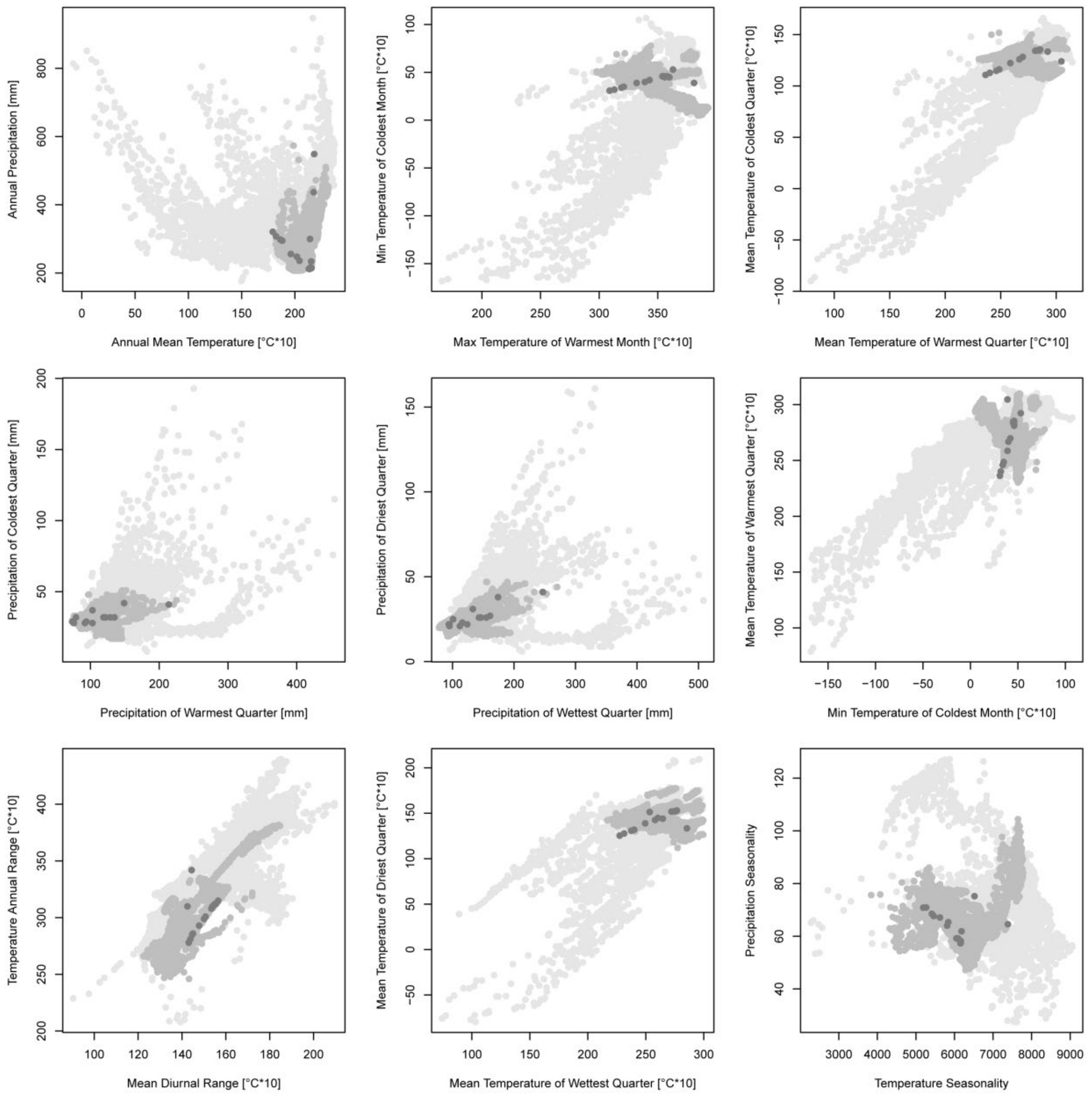

number of samples: 20

number of fossils: 0

sp60 − Trachemys venusta

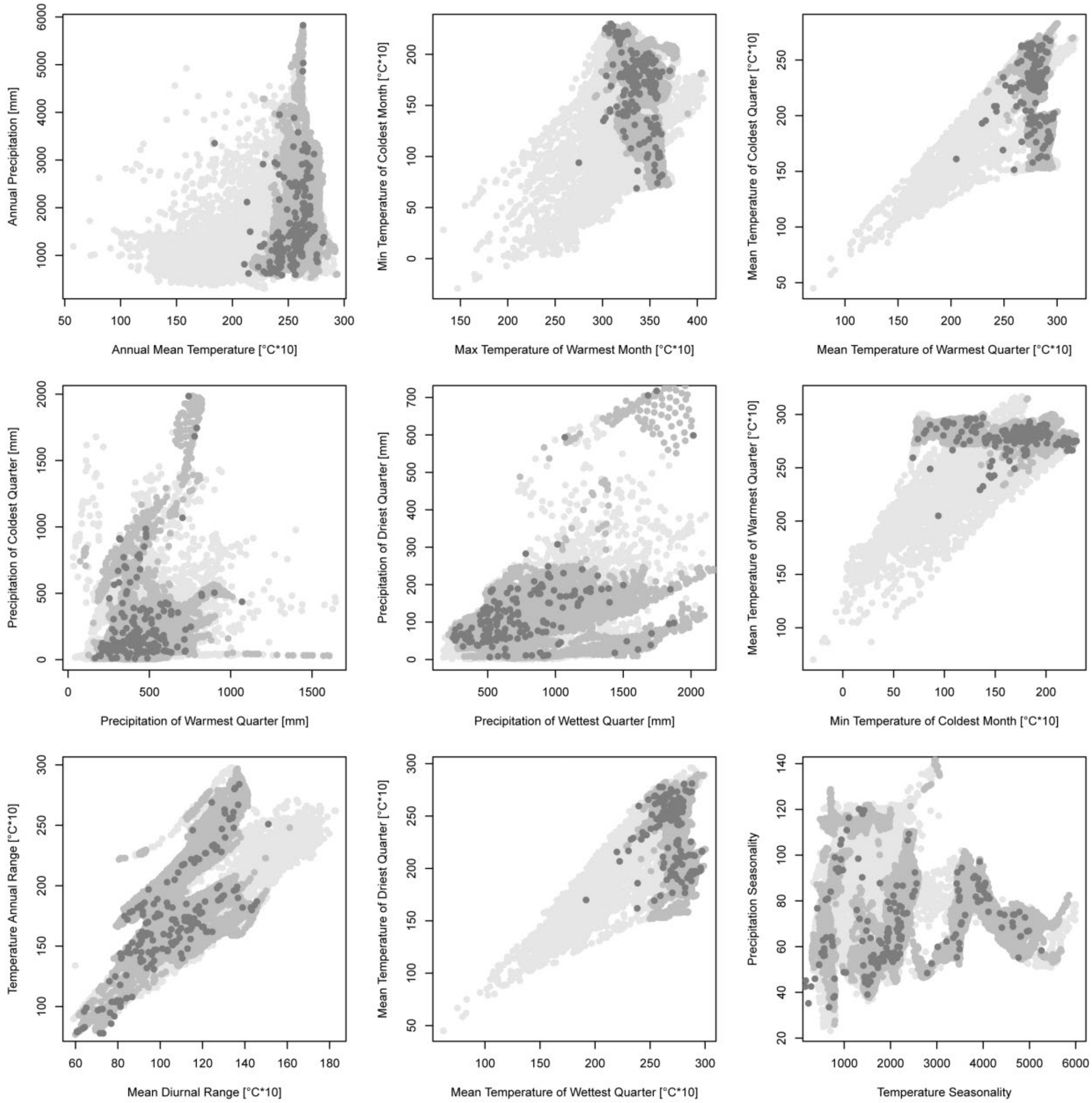

number of samples: 187

number of fossils: 0

sp61 − Trachemys yaquia

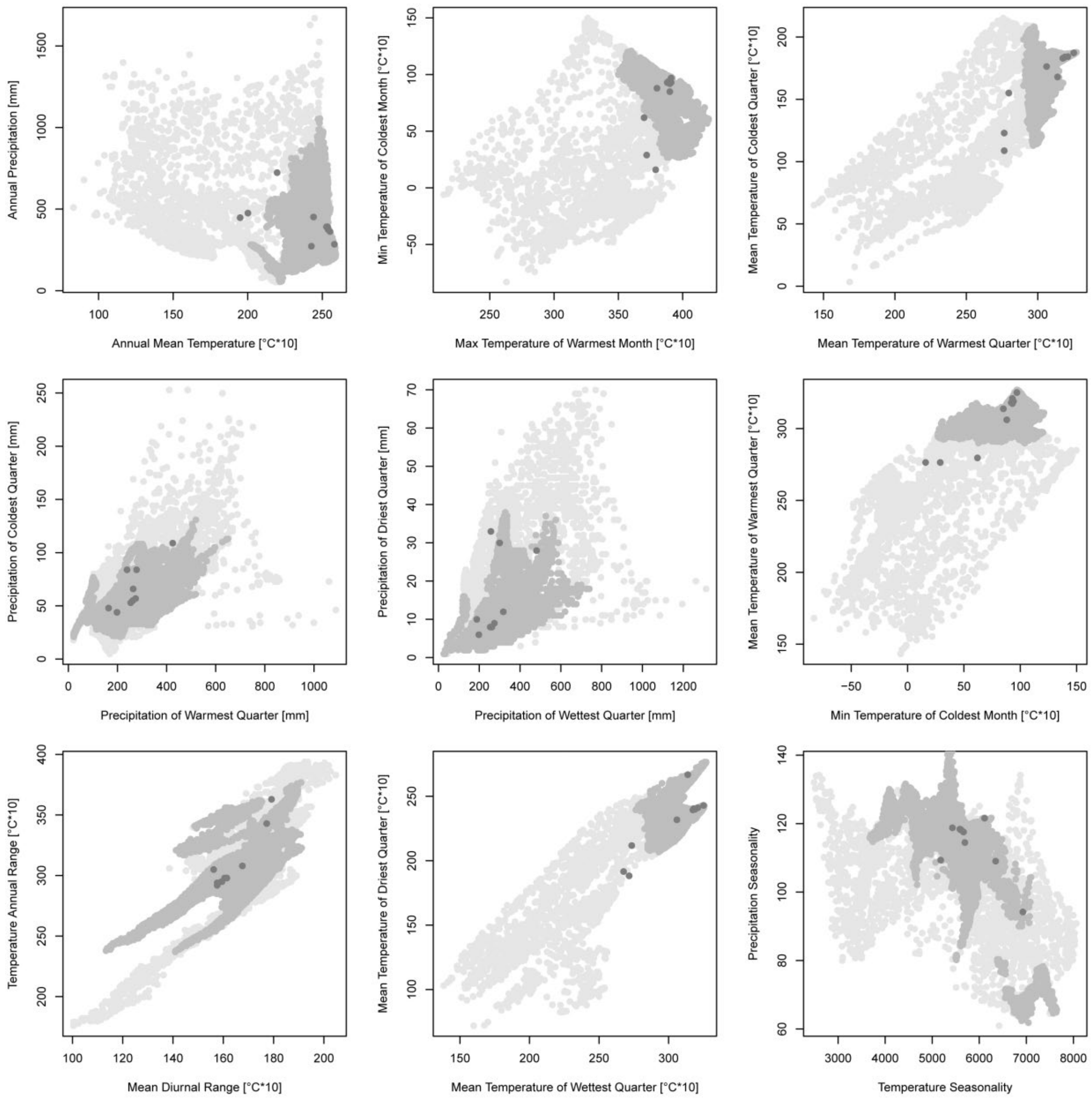

number of samples: 10

number of fossils: 0
