# Supplementary material for: Evaluating the Significance of Paleophylogeographic Species Distribution Models in Reconstructing Quaternary Range-Shifts of Nearctic Chelonians
Source: PLoS One. 2013 Oct 9;8(10):e72855. doi: 10.1371/journal.pone.0072855 (PMC3794015; doi:10.1371/journal.pone.0072855)
Supplement: Material S1 — Zip file containing Appendices S1 through S7. Appendix S1: Genbank accession numbers of sequences of four mitochondrial genes (COI, control region, cytb, ND4) and three nuclear genes (R35, RAG-1, TB29) used for phylogenetic analyses; Appendix S2: Plots showing the bioclimatic conditions within each species' currently available climate space, its potential niche as well as its realized niche both in terms of species' modern and fossil occurrences; Appendix S3: Fossils of Nearctic chelonians compiled from Holman [61] and supplemented with data obtained through the Paleo Biology Database (available through www.paleodb.org). Negative MESS scores indicate conditions within the species' current realized/potential niche and positive MESS scores indicate bioclimatic conditions outside of the current realized/potential niche; Appendix S4: Videos showing the results of the Maxent and PPGM analyses, i.e. distribution changes through time in comparison to current distributions according to Maxent, non-analogous climate and information on subspecies and phylogenetic lineages; Appendix S5: Comprehensive summary of data on thermal biology of 48 Nearctic chelonian species; Appendix S6: Dated molecular phylogeny for all 59 extant species of Nearctic turtles inferred by Bayesian analyses; Appendix S7: Summary statistics of each 100 Maxent models per species. (ZIP) [file pone.0072855.s001.zip › renamed_91f67/Appendix S6 Rödder et al. - PLos ONE.pdf]

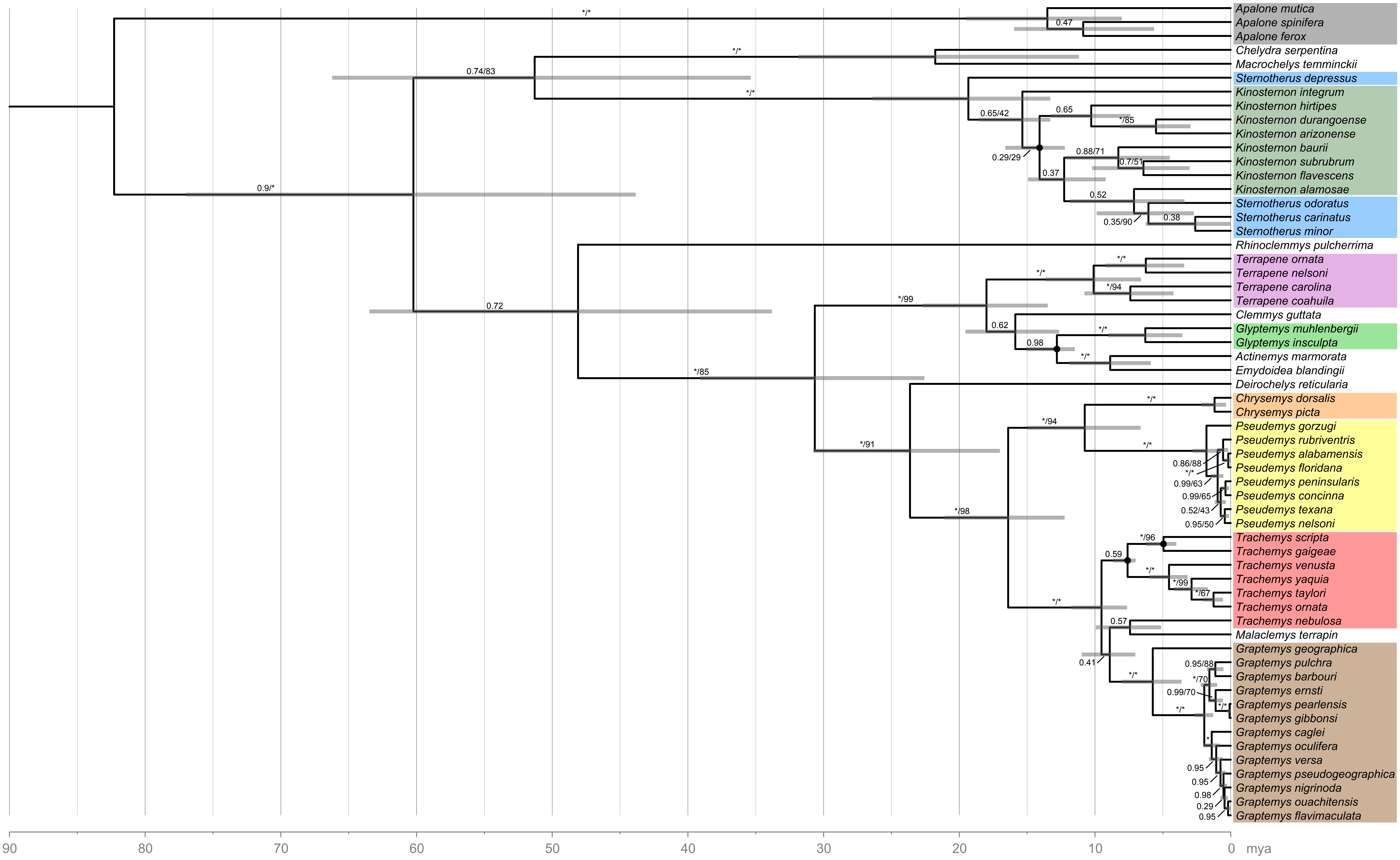

Rödder, D., A. M. Lawing, M. Flecks, F. Ahmadzadeh, J. Dambach, J. O. Engler, J. C. Habel, T. Hartmann, D. Hörnes, F. Ihlow, K. Schidelko, D. Stiels, P. D. Polly. 20XX. Evaluating the significance of palaeophyllographic species distribution models in reconstructing Quaternary range-shifts of Nearctic Chelonians. PLoS ONE X: XX – XX

Supplementary material S6: Dated phylogeny of 59 species of Nearctic turtles. Posterior probabilities (first value above branches) and Maximum Likelihood bootstraps (second value above branches), asterisks code values of 1 and 100, respectively. Grey bars illustrate 95% highest posterior density intervals of node ages. Nodes marked by a black circle were used for calibration.
